# A compendium of validated pain genes

**DOI:** 10.1002/wsbm.1570

**Published:** 2022-06-27

**Authors:** Eric Wistrom, Rebecca Chase, Patrick R. Smith, Zachary T. Campbell

**Affiliations:** ^1^ Department of Biological Sciences University of Texas at Dallas Richardson Texas USA; ^2^ Center for Advanced Pain Studies University of Texas at Dallas Richardson Texas USA

**Keywords:** pain genes, pain genetics, pain genomics

## Abstract

The development of novel pain therapeutics hinges on the identification and rigorous validation of potential targets. Model organisms provide a means to test the involvement of specific genes and regulatory elements in pain. Here we provide a list of genes linked to pain‐associated behaviors. We capitalize on results spanning over three decades to identify a set of 242 genes. They support a remarkable diversity of functions spanning action potential propagation, immune response, GPCR signaling, enzymatic catalysis, nucleic acid regulation, and intercellular signaling. Making use of existing tissue and single‐cell high‐throughput RNA sequencing datasets, we examine their patterns of expression. For each gene class, we discuss archetypal members, with an emphasis on opportunities for additional experimentation. Finally, we discuss how powerful and increasingly ubiquitous forward genetic screening approaches could be used to improve our ability to identify pain genes.

This article is categorized under:

Neurological Diseases > Genetics/Genomics/Epigenetics
Neurological Diseases > Molecular and Cellular Physiology

## Graphical abstract

An overview of pain signaling pathways, with tissue expression patterns of 242 validated pain genes.

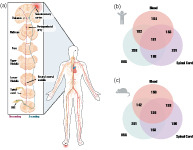

## 1 INTRODUCTION

Pain is pervasive and devastating. In the United States alone, chronic pain affects between 11% and 40% of adults and poses an annual cost of $560 to $635 billion in 2010 dollars (Dahlhamer, 2019; Gaskin & Richard, 2012). While common approaches to treating pain involve corticosteroids, nonsteroidal anti‐inflammatory drugs, and opioids, existing strategies fail to adequately address the unique challenges of chronic pain (Hylands‐White et al., 2016). Opioids are exemplary. Despite their immense analgesic potential in the treatment of acute pain, they activate reward centers in the mesolimbic dopaminergic system, which significantly increases their potential for abuse (le Merrer et al., 2009). Moreover, prolonged administration of opioids may result in sensitization and can paradoxically decrease the patient's nociceptive threshold to subsequent stimulation (Lee et al., 2011). Given the limited options available for treating severe pain, there is a pressing need for the identification of non‐opioid analgesic targets.

**FIGURE 1:**
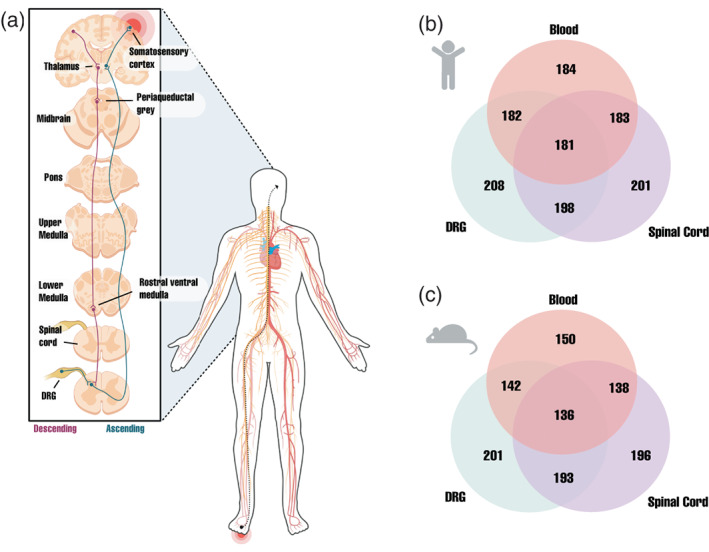
An overview of pain signaling and tissue expression patterns of pain genes. (a) Painful stimuli are detected by peripheral nociceptors. From the DRG, the signal propagates to the dorsal horn of the spinal cord, then to the thalamus, and finally to the somatosensory cortex. The descending pain pathway propagates signals from the somatosensory cortex back to the dorsal horn of the spinal cord via the thalamus, periaqueductal gray of the midbrain, and rostral ventral medulla. (b) Expression of pain genes in the DRG, brain, and blood in humans (top) and mice (bottom; Ray et al., 2018)

Pain generally falls into one of three categories: nociceptive, inflammatory, or neuropathic (Scholz, 2014; Woolf, 2020). Nociceptive pain is important in promoting injury avoidance and generally subsides after the healing process is complete. Noxious cues (e.g., extreme temperatures, mechanical stimulation, chemical irritants, etc.) can activate a specific type of sensory neuron called a nociceptor (Figure 1a; Basbaum et al., 2009; Julius, 2013; Woolf & Ma, 2007). The cell body of the nociceptor resides in a specialized ganglion located in either the dorsal root of the spinal nerve, called the dorsal root ganglion (DRG), or at the base of the trigeminal nerves in the dura mater, called the trigeminal ganglion (TG). Nociceptors are pseudounipolar and have a single process that extends from the soma to bifurcate into two axons. Of these, one axon forms a synapse with an interneuron in the spinal cord while the other innervates the viscera or the skin. When stimulated, nociceptors generate action potentials that transmit signals from the periphery to the spinal cord. This occurs when voltage‐gated sodium ion channels (Na_v_) open to allow an influx of sodium cations into the cell, which depolarizes the membrane and propagates an action potential. As the action potential arrives at the axon terminal, voltage‐gated calcium ion channels (Ca_v_ or VOCC) open to allow an influx of calcium cations into the cell. These ions bind calcium‐sensing proteins in the presynaptic terminal, which subsequently interact with *N*‐ethylmaleimide‐sensitive factor activating protein receptor (SNARE) proteins to promote the fusion of synaptic vesicles to the presynaptic membrane (Yam et al., 2018). Neurotransmitters are then released into the synaptic cleft via exocytosis so that they may bind their cognate ligand‐gated ion channels on the postsynaptic membrane of adjacent neurons. This generates a synaptic potential in the axon hillock domain of the neighboring neuron; the summation of multiple synaptic potentials serves to initiate a new action potential. The nociceptive signal is eventually transmitted to the spinal cord through A‐delta‐ and C‐fiber nociceptive afferents that synapse with the superficial laminae I‐II of the dorsal horn (Peirs & Seal, 2016). Stimulation of interneurons in the dorsal horn further increases intracellular calcium levels and activates presynaptic *N*‐methyl‐d‐aspartate (NMDA)‐type glutamate receptors, which triggers synaptic vesicle exocytosis (Dedek & Hildebrand, 2022; Peirs et al., 2015). This in turn leads to the release of excitatory neurotransmitters (glutamate) and neuropeptides (Substance P and CGRP) at the synaptic cleft, which facilitates transmission of the nociceptive signal and regulates long‐term changes in synaptic plasticity (Banerjee et al., 2016; Park & Luo, 2010; Petrenko et al., 2003). The nociceptive signal is subsequently transmitted from the dorsal horn to the spinal cord so that it may arrive at specific regions of the brain where it undergoes additional processing. Parallel to this ascending process, a descending pathway exists that modulates the transmission of nociceptive messages to higher processing centers (Millan, 2002).

In comparison to nociceptive pain, inflammatory pain results from tissue injury that provokes a local inflammatory response and pain hypersensitivity (Pinho‐Ribeiro et al., 2017; Woolf, 2020). This hypersensitivity can take two forms. In the first, innocuous stimuli are perceived as painful through a phenomenon called allodynia. In the second, noxious cues result in exaggerated pain responses—both in amplitude and duration—through a phenomenon called hyperalgesia. Hyperalgesia may be further classified as either primary or secondary, with primary hyperalgesia referring to hypersensitivity at the site of injury and secondary hyperalgesia extending to the surrounding areas (Sarkar et al., 2000; Treede et al., 1992). Primary hyperalgesia is caused by a process called peripheral sensitization through which inflammatory mediators and local factors trigger intracellular signaling pathways in primary afferents, thereby altering the function or expression of receptor molecules and voltage‐gated ion channels (Barragan‐Iglesias et al., 2021; de la Peña, Kunder, et al., 2021; Gangadharan & Kuner, 2013; Melemedjian et al., 2010; Prescott & Ratté, 2017; Woolf & Thompson, 1991). At the same time, peptidergic C fibers can release pro‐inflammatory neuropeptides to initiate a biochemical signaling cascade known as neurogenic inflammation. Nociceptors can also provoke long‐term changes in the production of factors implicated in nociceptive circuits in the central nervous system through a process known as central sensitization (Latremoliere & Woolf, 2009; Woolf, 1983). While these changes in synaptic plasticity may facilitate the transition from acute to chronic forms of pain, inflammatory hypersensitivity often subsides as local inflammation is reduced.

Neuropathic pain is distinct from nociceptive and inflammatory pain in that it is caused by nerve lesions that alter the activity of nerve fibers. While neuropathic pain most often originates from injury, viral infection, disease, or neurotoxins affecting the peripheral nervous system, it may also originate in the central nervous system (Colloca et al., 2017; Costigan et al., 2009; Woolf, 2020). Similar to inflammatory pain, neuropathic pain is associated with pain hypersensitivity and can lead to sensitization. For instance, peripheral axonal injury can alter gene expression in the affected sensory neurons and in the spinal cord, leading to peripheral sensitization (Cobos et al., 2018; Raithel et al., 2018). Immune cells may also modulate the activity of nociceptors and spinal neurons involved in nociceptive signaling pathways, resulting in central sensitization (Moalem & Tracey, 2006). Given that neuropathic pain results from nerve damage, it may manifest with either loss‐of‐function (i.e., sensory deficits) or gain‐of‐function (i.e., hypersensitivity) behavioral abnormalities (Prescott & Ratté, 2017).

In addition to these categories, a fourth descriptor for chronic pain has been proposed: nociplastic pain (Kosek et al., 2016). While mechanistically distinct from nociceptive, inflammatory, and neuropathic pain given that it is not correlated with the activation of nociceptors nor with the onset of neuropathy, the precise mechanisms that underlie nociplastic pain are not well understood (Fitzcharles et al., 2021). Given the lack of a rigorously established causality, there is ongoing debate as to whether nociplastic pain indeed constitutes a novel pain category (Granan, 2017).

Multiple tissues and cell types are involved in pain signaling. It is thus perhaps unsurprising that hundreds of genes have been linked to pain. While multiple attempts have been made to inventory these genes (Diatchenko et al., 2007; LaCroix‐Fralish et al., 2007; Meloto et al., 2018), we sought to fulfill four objectives in this review. First, we report a list of high confidence genes that have been validated in genetic models. Second, we discuss similarities, expression patterns, and functional categories that are enriched in the data. Third, we outline historical trends in the field to illustrate promising areas of inquiry. Fourth and finally, we provide a much‐needed repository of recent developments in the field that have taken place since 2015, when Jeffrey Mogil's Pain Genes Database was last updated (LaCroix‐Fralish et al., 2007). In so doing, we report a compendium of validated pain genes that may serve as both a resource to the field and a repository for hypothesis generation.

## 2 METHODS

To generate our list of pain genes, we conducted a query on PubMed in August 2021 using the terms “gene” AND “pain” AND “deletion”. These keywords were chosen to limit our query to genes that had been validated in knockdown or knockout animal models. This returned a set of 721 abstracts, which were subsequently vetted for inclusion in our review from August to September 2021. We recognize that the limited search parameters afforded by our query have undoubtedly led to omissions, and we apologize for any oversights. We excluded studies that lacked genetic validation coupled to a behavioral assay, such as genome‐wide association studies (GWAS), genome‐wide linkage studies (GWLS), and single‐nucleotide polymorphisms (SNPs); mutations not linked to a specific gene; and, finally, pharmacology and/or in vivo voltage clamp and electrophysiology studies that were not validated by behavioral assays. Peak expression was not used as a criterion for our list.

All analyses and initial plots were created in R with RStudio (R Core Team, 2017; RStudio Team, 2020). Single cell data was preprocessed as previously described (Usoskin et al., 2014). Clustering and visualization were performed using the Uniform Manifold Approximation and Projection (UMAP) algorithm and R Seurat package 4.0. For the single‐cell analysis of pain gene expression, only those genes that had their maximum expression in either human or mouse DRG or spinal cord were considered (Ray et al., 2018). Expression levels of each pain gene were gathered for each cell‐type‐specific cluster.

## 3 RESULTS

Our literature search identified 242 genes (Table S1). We made use of existing datasets to ask if pain genes were preferentially expressed in either the DRG, the spinal cord, or cells present in the whole blood of humans and mice (Figure 1b; Ray et al., 2018). There was roughly an equal spread of genes expressed in the DRG (201 genes in mice; 208 genes in humans) and spinal cord (196 genes in mice; 201 genes in humans), with a significant overlap in genes that were co‐expressed in both tissue types (193 genes in mice; 198 genes in humans). While fewer genes were expressed in whole blood where immune cells are present (150 genes in mice; 184 genes in humans), a large number of these were co‐expressed in the DRG (142 genes in mice; 182 genes in humans) or the spinal cord (138 genes in mice; 183 genes in humans). Interestingly, many of the pain genes were co‐expressed in all three tissue types (136 genes in mice; 181 genes in humans). We did not analyze the expression of pain genes in the central nervous system as the brain tissues used in the RNA‐seq datasets were not implicated solely in the perception of pain.

**FIGURE 2:**
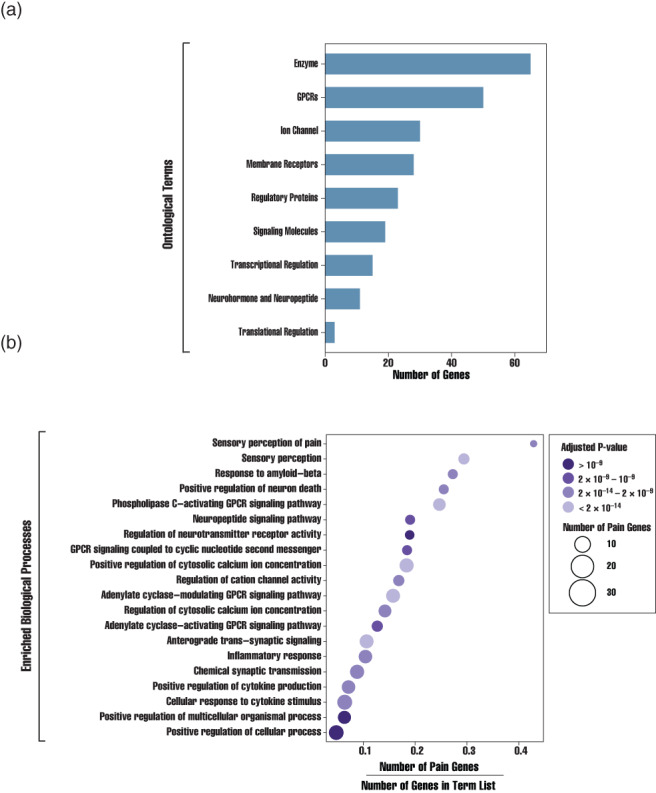
Ontological analysis of pain genes. (a) Histogram depicting the number of pain genes versus functional class. (b) Dot plot of GO term enrichment for pain genes. Color indicates the adjusted *p*‐value and the size of the dot represents the number of pain genes associated with the term (E. Y. Chen et al., 2013; Kuleshov et al., 2016; Z. Xie, Bailey, et al., 2021). The ratio of pain genes linked to the specified term to the total number of genes associated with a term—also known as the gene ratio—is shown along the *x*‐axis.

Next, we examined the ontological feature of pain genes (Figure 2). The majority were associated with enzyme activity or GPCRs (Figure 2a). Certain features were highly enriched across our dataset, including the sensory perception of pain (GO.0019233), sensory perception (GO.0007600), response to amyloid‐beta (GO.1904645), positive regulation of neuron death (GO.1901216), and phospholipase C‐activating G protein‐coupled receptor signaling pathway (GO.0007200; Figure 2b). While some of these terms are intuitive, others are less clear. Response to amyloid‐beta is exemplary. Given that microglial activation has been linked to nerve injury‐induced pain hypersensitivity and that there is some indication that amyloid‐beta results in the activation of microglia, microglial activation might be a convergent effect of both processes (G. Chen et al., 2018; Combs et al., 2001).

**FIGURE 3:**
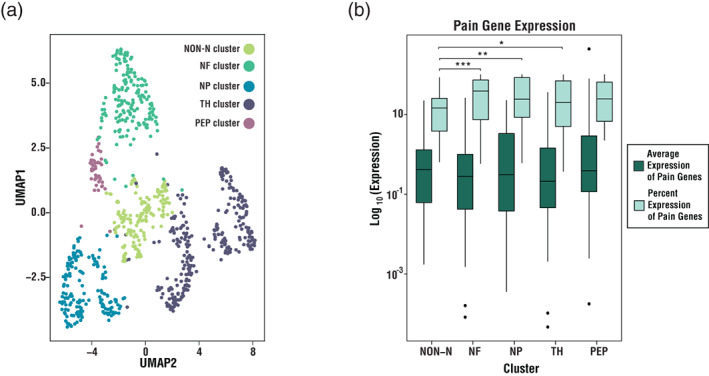
Single‐cell analysis reveals pain genes are expressed in sensory neurons at a higher rate than non‐neuronal cells in the DRG. (a) Uniform Manifold Approximation and Projection (UMAP) plot of murine DRG single‐cell data (Usoskin et al., 2014), where color indicates the specific cluster with which a cell is associated. The five clusters identified include: non‐neuronal cluster (NON‐N, green); neurofilament cluster (NF, teal); nonpeptidergic cluster (NP, blue); tyrosine hydroxylase cluster (TH; purple); and, peptidergic cluster (PEP, pink). (b) Box plot showing the average cell‐type‐specific expression, in log scale (*y*‐axis), of pain genes, with their maximum expression in the murine or human DRG or spinal cord (Ray et al., 2018). The total percent expression of the specified pain genes in a given cluster (*x*‐axis) is displayed in light green, while the total average expression of the genes in a given cluster is displayed in dark green. One‐way ANOVA with Tukey's multiple comparisons test. The average expression is not significant. Percent expression: *F*(4) = 4.761, *p* < 0.001; NON‐N versus NF ***p < 0.001, NON‐N versus NP **p < 0.01, NON‐N versus TH *p < 0.05, and NON‐N versus PEP is not significant at *p* = 0.1.

We also examined the expression of DRG‐ and spinal cord‐enriched genes in murine DRG single cells (Figure 3; Usoskin et al., 2014). Our goal was to understand how neuronal transcripts are expressed in different cell types found in the DRG. We considered five distinct clusters: non‐neuronal cluster (NON‐N), neurofilament cluster (NF), nonpeptidergic cluster (NP), tyrosine hydroxylase cluster (TH), and peptidergic cluster (PEP). The average expression of genes linked to pain remained relatively consistent across all categories. However, we found an increase in the percent of cells within specific neuronal clusters (neurofilament, nonpeptidergic, tyrosine hydroxylase) when compared to the non‐neuronal cluster that expresses pain genes (Figure 3b). This implies that pain genes are more often expressed in neurons than non‐neurons. Yet the expression levels of the genes that are detected are relatively constant. This could stem from the relatively low read depth per cell obtained by single cell approaches. An intriguing exception lies within the peptidergic cluster of cells. The trend in this cell type was similar to the other neuronal cell types but was not significant. While the trend is consistent with the pervasive role of peptidergic neurons in pain, we suspect that the small number and dense clustering of this cell type impact the n value and variance when calculating the statistical tests. Thus, the overall tendency is that pain genes are more often expressed in sensory neurons than non‐neurons, specifically in the DRG.

In the following sections, we describe what is known about specific genes and pathways that are linked to pain. This discussion is intended to provide a useful framework for understanding the tremendous diversity of pain genes, while at the same time establishing a comprehensive overview of the best‐validated models that have arisen over the past three decades. The literature review is organized around six functional categories: voltage‐gated and ligand‐gated ion channels; G protein‐coupled receptors (GPCRs); neuropeptides, neurotransmitters, and neurotrophins; growth factors, hormones, and cytokines; enzymes and enzyme‐linked receptors; and, transcriptional and translational control and mRNA processing. While this categorization is imperfect and has in select cases resulted in some ambiguity—such as the placement of neurotrophins with neuropeptides and neurotransmitters given their similar localization, despite neurotrophins being more precisely understood as a subset of growth factors acting on neural substrates—our system is nonetheless meant to provide a global understanding of the complex relationships that underlie nociceptive signaling, which may in turn highlight opportunities for further study.

### 3.1 Voltage‐gated and ligand‐gated ion channels

Voltage‐gated ion channels are of particular interest in pain given their central role in the propagation of action potentials that underlie intercellular communication. While these channels are normally closed at the cell's resting potential, they rapidly open as the membrane's electrochemical gradient approaches a specifically defined threshold voltage (Sigworth, 1994). This in turn allows for an influx of sodium or calcium cations into the cell, which results in depolarization and the propagation of an action potential along the axon to the synaptic boutons (Catterall, 1995; de Lera Ruiz & Kraus, 2015). Repolarization subsequently occurs as potassium‐gated ion channels open to allow potassium cations to move freely across the membrane until the cell's resting potential is restored (Bean, 2007). This delayed counterflow plays an essential role in shaping the transduced action potential and aids in propagating the cellular signal throughout the peripheral nervous system via a process known as saltatory conduction. Other classes of ion channels—termed ligand‐gated ion channels or ionotropic receptors—generate action potentials following the binding of a chemical messenger (Lemoine et al., 2012; S. Li et al., 2014). While there are several strategies that may be used to modulate cell‐surface receptors and their associated transmembrane channel, most involve the use of small molecules to occlude pores or to prevent interactions with cytosolic protein partners.

Of the three voltage‐gated channels directly involved in regulating membrane potentials—those being voltage‐gated sodium (Na_v_), calcium (Ca_v_ or VOCC), and potassium (K_v_) ion channels—Na_v_ channels have emerged as dominant targets for pain. Of the nine human Na_v_ channels, Na_v_1.7, Na_v_1.8, and Na_v_.9 are the best studied and characterized (Cregg et al., 2010). Research into Na_v_ channels is often traced to two GWAS conducted in 2004 and 2005 on primary erythromelalgia patients that revealed gain‐of‐function mutations in the human *SCN9A* gene encoding Na_v_1.7 (Dib‐Hajj et al., 2005; Yang et al., 2004). This association was validated by a 2004 conditional knockout of the murine *Scn9a* gene in which animals displayed reduced mechanical and thermal sensitivities under baseline and inflammatory conditions (Nassar et al., 2004). Two years later, sequencing of three families with congenital insensitivity to pain confirmed a channelopathy in SCN9A and therein established Na_v_1.7 as a key mediator of nociceptive pain (Cox et al., 2006). Subsequent work in murine knockout models has confirmed that SCN9A channelopathies not only contribute to increased baseline and inflammatory nociceptive thresholds, but also attenuate select chemical sensitivities and mediate itch response to specific pruritogens (Hoffmann et al., 2018; Shields et al., 2018). This last finding is of particular interest given that itch is mediated by a different spinal mechanism than nociception, which suggests expansive roles for SCN9A in sensory neuron function (Han et al., 2012).

SCN9A has been extensively studied as a potential analgesic target. Yet, despite over a decade of research into Na_v_1.7 inhibitors, to the best of our knowledge, none have successfully completed clinical trials. There are multiple challenges that have hindered drug development efforts, including the low affinity with which Na_v_ channels binds pharmaceutical agents, the need for channel subtype selectivity, and the distribution pattern of Na_v_1.7—particularly in the vagal system (Muroi et al., 2011). The need for specificity is particularly well illustrated by the difficulties surrounding the development of Biogen's small‐molecule inhibitor vixotrigine (raxatrigine) whose research into painful lumbosacral radiculopathy was discontinued following phase II failure in 2018. Despite having been identified as a state‐dependent inhibitor of Na_v_1.7 in 2014, a 2016 study showed that raxatrigine was relatively nonspecific and displayed affinity with multiple Na_v_ channels in a florescence loss in photobleaching (FLIP) membrane profile assay (Bagal et al., 2014; Deuis et al., 2016). Attempts to remedy specificity have led to the development of many small‐molecule inhibitors—the majority of which have been derivatives of aryl sulphonamides—that preferentially target voltage‐sensing domain 4 of Na_v_1.7 (McCormack et al., 2013). While this has aided in increasing target specificity, this has not translated into the discovery of more efficient therapeutics with higher binding affinities. Alternative methods of circumventing these limitations are currently under development, including research into tarantula venom peptides that selectively bind voltage‐sensing domain 2 as well as novel methods of indirectly targeting Na_v_1.7 via the protein partner CRMP2 (Kingwell, 2019). These promising new approaches may overcome challenges associated with direct modulation of Na_v_1.7.

Despite the inherent difficulties in developing SCN9A‐targeted compounds, Na_v_ channels remain enticing therapeutic targets. For instance, knockout of *Scn10a*, which encodes for Na_v_1.8, reduces inflammatory sensitivity to mechanical and thermal stimulation, and reduces spontaneous writhing responses to noxious chemical stimuli (Abrahamsen et al., 2008; Akopian et al., 1999; Laird et al., 2002). While two conditional knockout studies indicated that SCN10A deficiency fails to alter basal nociceptive thresholds (Agarwal et al., 2004; Stirling et al., 2005), others have indicated that SCN10A deletion contributes to deficits in basal mechanical, thermal, and cold sensation (Abrahamsen et al., 2008; Akopian et al., 1999; Zimmermann et al., 2007). This discrepancy could be explained by the nature of the deletion given that the negative results were seen in nociceptor‐specific models while the positive results were seen with systemic deletion of SCN10A.

Knockout of *Scn11a*, which encodes Na_v_1.9, similarly reduces mechanical and thermal sensitivities to a variety of inflammatory mediators and cold stimuli (Amaya, Wang, et al., 2006; Lolignier et al., 2011, 2015; Priest et al., 2005). However, SCN11A‐deficient animals fail to show deficits in basal mechanical and thermal nociception (Amaya, Wang, et al., 2006; Lolignier et al., 2011). This contrasts with Na_v_1.7 and Na_v_1.8 phenotypes and raises the possibility that normal sensory functioning could be preserved with pharmacological blockade of Na_v_1.9. Combined with a recent study that showed that SCN11A deletion abrogated symptoms of triptan‐overuse headache (Bonnet et al., 2019), SCN11A might be useful for future pharmaceutical research.

Despite the utility of genetic models in understanding pain‐associated behaviors, there are inherent complications that may arise when attempting to translate results from preclinical models to humans (Floris et al., 2018; Hackam & Redelmeier, 2006; Plenge et al., 2013). For instance, background mutations—including copy number variants and polymorphisms—can give rise to genetic effects not typically associated with inbred mouse strains, while genetic interactions can result in substantial complications. This was recently demonstrated in a joint effect study that investigated the synthetic ablation of *Scn9a* with *Oprd1*, *Oprk1*, and *Oprm1* coding for the three opioid receptors—a project which was at least in part inspired by an earlier knockout study that implicated SCN9A in reversing naloxone‐induced mechanical and thermal analgesia (Minett et al., 2015). While co‐deletion of SCN9A and OPRD1 or OPRM1 decreased thermal analgesia compared to deletion of SCN9A alone, this reduction was smaller than what was seen in SCN9A‐deficient animals who were injected with the nonspecific opioid antagonist naloxone (Pereira et al., 2018). This suggested a cumulative effect of OPRD1 and OPRM1, which was confirmed when the deletion of all three opioid receptors decreased analgesia to a level equivalent to that of SCN9A‐deficient animals injected with naloxone. At the same time, neither genetic nor pharmacological disruption of OPRK1 produced a significant reduction in analgesia in any of the experimental conditions. Thus, OPRK1—unlike OPRD1 and OPRM1—is not involved in the SCN9A opioid‐mediated analgesic pathway (Pereira et al., 2018).

Similar to Na_v_ channels, many members of the transient receptor potential (TRP) family of ion channels have been validated as playing key roles in somatosensation and pain. Of particular note is the nonselective cation channel transient receptor potential ankyrin 1 (TRPA1). TRPA1‐deficient mice display increased mechanical and thermal thresholds when challenged with a variety of inflammatory and chemical stimuli (Bautista et al., 2006; Cattaruzza et al., 2010; de Oliveira et al., 2014; Kwan et al., 2006; Marone et al., 2018; Norões et al., 2019; Puma et al., 2019; Trevisan et al., 2013, 2014). TRPA1 has also been implicated in the development of neuropathic cold sensitivity following both oxaliplatin and cisplatin administration, and reperfusion injury (de Logu et al., 2020; Nassini et al., 2011). Despite the channel's relatively well‐validated role in mediating neuropathic cold sensitivity, its involvement in mediating basal cold sensitivity is currently subject to debate: while a 2006 study showed that *Trpa1*‐null mice exhibited deficiencies in cold somatosensation, two others have indicated that TRPA1 disruption fails to alter basal thresholds (Bautista et al., 2006; Knowlton et al., 2010; Kwan et al., 2006). It is unclear if this difference is due to experimental design or a compensatory mechanism of another unaffected TRP channel such as transient receptor potential melastatin 1 (TRPM1)—which is itself required for neural and behavioral response to noxious cold temperatures and cold mimetics (Knowlton et al., 2010).

Similar to TRPM1, transient receptor potential melastatin 8 (TRPM8) has been validated as a key mediator of cold sensitivity (Bandell et al., 2006). While TRPM8 deficiency results in decreased nociceptive sensitivity to cold and cold mimetics, it does not alter behavioral responses to acute thermal or mechanical stimulation (Bautista et al., 2007; Colburn et al., 2007; Dhaka et al., 2007; Knowlton et al., 2010).

Knockout models into other TRP channels have produced more ambiguous results. For instance, two recent studies into transient receptor potential canonical 5 (TRPC5) produced conflicting behavioral phenotypes. While a 2017 study indicated that both genetic deletion and pharmacological blockade of TRPC5 increased CFA‐induced mechanical and thermal sensitivity, a 2021 study inversely showed that disruption of TRPC5 decreased CFA‐induced mechanical—but not thermal—sensitivity (Alawi et al., 2017; Sadler et al., 2021). A third report found that deletion of TRPC5 increased mechanical sensitivity following partial meniscectomy and intraarticular injection of monoiodoacetate (MIA)—the latter of which was used to induce a model of osteoarthritis (de Sousa Valente et al., 2020). The increased sensitivity following MIA injection was also correlated with increased c‐Fos expression in cultured neurons, which is significant given that c‐Fos has been implicated as playing a key role in the transition to chronic forms of inflammatory and neuropathic pain via the downstream expression of dynorphin (Ahmad & Ismail, 2002; Marvaldi et al., 2020; de la Peña, Kunder, et al., 2021).

While an incredible amount of research has been conducted into transient receptor potential vanilloid 1 (TRPV1) since its successful cloning in 1997 (Caterina et al., 1997), the specificity with which the channel acts in response to local stimuli remains somewhat enigmatic. For instance, a 2005 study indicated that deletion of TRPV1 decreased nociceptive sensitivity to heat‐induced mechanical and thermal stimuli, yet increased neuropathic mechanical sensitivity to diabetic and cisplatin‐evoked toxic polyneuropathy (Bölcskei et al., 2005). Given these results, endogenous TRPV1 receptors appear to play a pronociceptive role in select models of acute tissue injury and an antinociceptive role in chronic polyneuropathic conditions. A similar nuance was seen in four studies focusing on TRPV1‐mediated inflammatory response: while one indicated that genetic disruption of TRPV1 decreased thermal—but not mechanical—sensitivity following intraplantar administration of CFA and mustard oil, three others showed significant decreases in both mechanical and thermal sensitivities following the administration of various inflammatory mediators (Caterina et al., 2000; Flynn et al., 2014; Liao et al., 2017; Szabó et al., 2005).

Given the broad involvement of TRP channels in pain, it is perhaps unsurprising that they have been the subject of intensive drug discovery and development efforts. Yet, similar to what was seen with Na_v_‐related therapeutics, no selective TRP analgesics have been approved by the FDA. Whereas druggability and selectivity served as the principal roadblocks in the development of Na_v_‐targeted pharmaceuticals, the diverse physiological roles of TRP channels have historically served as their primary source of complications (Koivisto et al., 2021). TRPV1 is exemplary. Given that TRPV1 serves both an afferent function in integrating painful stimuli and an efferent function in initiating neurogenic inflammation, there is a heightened risk of the channel's disruption having undesirable side effects. This ultimately contributed to many first‐generation TRPV1 antagonists being withdrawn from clinical trials upon discovery of unacceptable on‐target effects like febrile reactions (AMG517) and burn injuries (MK2295; Gavva et al., 2008; Koivisto et al., 2021; R. Eid, 2011).

While Na_v_ and TRP channels have captivated pharmaceutical research and development over the past two decades, a number of other ion channels have likewise served as recurrent targets of interest. Perhaps unsurprisingly given their central role in regulating membrane potentials, various subunits of voltage‐gated calcium (Ca_v_ or VOCC; Murakami et al., 2002; Neely et al., 2010; Nissenbaum et al., 2010; Patel et al., 2013; Saegusa et al., 2001) and voltage‐gated potassium (K_v_; Alloui et al., 2006; Tsantoulas et al., 2018; X. Zhao et al., 2013) channels have been linked to pain. Ionotropic glutamate receptors have likewise served as recurrent targets given glutamate's central role in neural activation. Of particular note, the *N*‐methyl‐d‐aspartate (NMDA) receptor GRIN1 has been implicated in various forms of inflammatory and neuropathic pain, although full characterization of its phenotype remains tenuous given conflicting behavioral results (Brifault et al., 2020; Cheng et al., 2008; Inquimbert et al., 2018; South et al., 2003; Weyerbacher et al., 2010). Two studies into GRIN2A and GRIN2B have likewise indicated their broad involvement in nociceptive processing, although their characterization is too limited by a lack of replicate studies (Inoue et al., 2000; Wei et al., 2001).

### 3.2 G protein‐coupled receptors

GPCRs are integral to the neuroplasticity and neuropharmacology of pain. Despite GPCRs forming one of the largest and most diverse families of mammalian protein families, their structure is exceedingly well conserved and consists of seven transmembrane domains linked by alternating intracellular and extracellular loops. GPCRs are activated when ligands bind to the extracellular N‐terminus domain or to binding sites within the transmembrane helices, which induces a conformational change of the receptor. This leads to the coupling and activation of intracellular guanine nucleotide‐binding proteins as GDP is exchanged for GTP, which triggers the dissociation of the alpha subunit of the heterotrimeric G protein complex from the beta and gamma subunits. The three subunits subsequently act on their preferred targets. While the beta and gamma subunits form a dimer that acts on enzymes and ion channels via downstream signaling cascades and scaffolding complexes (Neves et al., 2002; Pan et al., 2008; Sadja et al., 2003), the alpha subunit typically serves a modulatory role in mediating receptor coupling specificity through its downstream signaling with GTP (Amaya, Shimosato, et al., 2006; Ivanina et al., 2004; Jeong & Ikeda, 2000; Leaney et al., 2000; Pan et al., 2008). Alternatively, the alpha subunit may directly interact with activating enzymes that control hormone signaling and neurotransmission. The activation of beta‐type phospholipase C (PLC‐beta) by the G_q_ alpha subunit (Dowal et al., 2006; Kamato et al., 2015; D. Wu et al., 1993), as well as the stimulation of the cAMP‐dependent pathway via G_s_ alpha subunit activation of adenylyl cyclase (Sassone‐Corsi, 2012), serve as two particularly well‐known examples.

Opioid receptors are among the best characterized GPCRs. Of the four major subtypes, the majority of knockout models have focused on three: the delta‐opioid receptor (OPRD1), the kappa‐opioid receptor (OPRK1), and the mu‐opioid receptor (OPRM1). As would be expected given their inhibitory function, the disruption of these three targets reduces the analgesic response to a variety of endogenous ligands (Corder et al., 2017; Fuchs et al., 1999; Matthes et al., 1996; McLaughlin et al., 2003; Nozaki et al., 2012; Schepers et al., 2008; Simonin et al., 1998; Sora et al., 1997; Weibel et al., 2013). Moreover, their disruption decreases basal, inflammatory, and neuropathic thresholds to a variety of noxious insults (Martin et al., 2003). Despite this widespread validation of their analgesic effect, some conflicting results have manifested in conditional knockout studies of *Oprm1*. For instance, when morphine was intrathecally administered to mice with OPRM1 conditionally deleted from the DRG, the mice showed significantly reduced analgesia; however, these same mice showed normal antinociceptive behavior when morphine was administered subcutaneously (Corder et al., 2017). This result was recapitulated in wild‐type mice via the administration of the blood–brain barrier (BBB) impermeable mu‐opioid receptor (MOR) antagonist methylnaltrexone bromide prior to morphine infusion. These mice not only showed a dose‐dependent reduction in the onset of analgesic tolerance and reduced opioid‐induced hypersensitivity following the injection of morphine, but also failed to manifest symptoms of physical withdrawal following the cessation of treatment. Taken together, these results point to a local involvement of OPRM1 and suggest that spinal opioid antinociception primarily results from presynaptic MOR signaling in nociceptors. Moreover, they highlight the therapeutic potential of peripherally restricted MOR antagonists that could limit the pronociceptive and addictive side effects of prolonged opioid use while simultaneously preserving their analgesic effect. Nonetheless, a central problem remains: given that opioids predominantly act on the central nervous system, their full therapeutic potential is withheld given their inherent potential for addiction‐related neuroplasticity.

Other GPCRs have likewise been the subject of extensive scrutiny. Metabotropic glutamate receptors (mGlurRs) are a key type of glutamate receptor, which is itself the central nervous system's principal excitatory neurotransmitter and a precursor to gamma‐Aminobutyric acid (GABA). While a multitude of studies has pointed to mGluRs as being intimately involved in nociceptive processing, these studies have primarily focused on pharmacology (Bhave et al., 2001; Chiechio & Nicoletti, 2012; Crupi et al., 2019; Karim, Wang, & Gereau 4th, 2001; Gerber et al., 2000; H.‐J. Hu et al., 2002, 2007; W. Li & Neugebauer, 2004; Maione et al., 2000; Marabese et al., 2007; Palazzo et al., 2008; Ren et al., 2011; Ren & Neugebauer, 2010; Walker et al., 2001). This is not to say that mGluRs have been completely neglected in knockdown and knockout models. For instance, a 2010 study that selectively deleted GRM5 in the central nucleus of the amygdala decreased formalin‐induced mechanical sensitivity, while two antisense oligonucleotide knockdowns of GRM1 decreased basal thermal sensitivity and neuropathic mechanical, thermal, and cold sensitivities following chronic constriction injury‐induced neuropathy (Fundytus et al., 2001; Kolber et al., 2010; Young et al., 1998).

Similar to their ionotropic counterpart GABA_A_, various metabotropic GABA_B_ receptor subtypes have been linked to pain (Benke, 2020; Enna & McCarson, 2006; Fischer, 2017; Y. Luo, Kusay, et al., 2021). Nonetheless, their characterization remains somewhat tenuous due to conflicting behavioral results, whereas Schwann's cell‐specific deletion of GABBR1 increased acute mechanical and thermal sensitivities, neuron‐specific deletion of GABRG2 decreased acute thermal sensitivity, while leaving basal mechanonociception unaffected (Faroni et al., 2014; Leppä et al., 2011). Given GABA's inhibitory role, this latter result is intriguing as the disruption of GABRG2 would be expected to either increase or not affect baseline sensitivities. While this divergence could be explained by differences in the identity and specificity of each ablation, another plausible explanation lies with confounding developmental effects that might have affected GABRG2‐deficient mice given that the GABRG2‐deficient mice showed profound deficits in motor skills and spatial learning. This raises the possibility that their deficits were not necessarily linked to a true analgesic effect, but rather to a nonspecific sensory impairment.

This lack of consensus certainty raises important questions regarding GABA_B_'s functional role given the widespread pharmacological validation of GABA in pain. For instance, administration of the selective GABA_B_ agonist baclofen (beta *p*‐chlorophenyl‐GABA) has long been known to hold significant analgesic and anti‐hyperalgesic properties; however, its therapeutic benefit has been severely limited by the drug's sedative side effects and by the fact that it requires active transport across the BBB (Malcangio, 2018; Shaye et al., 2021). While attempts have been made to circumvent these limitations by developing more bioavailable compounds for the central nervous system and by developing compounds that might specifically target peripheral GABA_B_ receptors, baclofen remains the sole selective GABA_B_ agonist on the market (Durant et al., 2018). Alternative avenues of research have thus turned to allosteric modulators that can modulate GPCR signaling without replacing their endogenous ligands, which has led to the recent discovery of a number of positive allosteric modulators (PAMs) of GABA_B_. PAMs provide a promising alternative to traditional GABA_B_ agonists or antagonists given that they can selectively potentiate response to the receptor's endogenous agonist while binding distal sites—thereby allowing for the positive regulation of GABA_B_ activity at the neuronal synapses where GABA is released. The analgesic potential of PAMs has been validated in knockout models, such as in a 2015 study in which GABA_B1_ formed a complex with TRPV1 in sensory neurons that counteracted inflammatory pain via a noncanonical, GABA_B2_‐independent pathway (Hanack et al., 2015).

Similar to the neurotransmitters glutamate and GABA, many other neuropeptides play integral roles in pain. The tachykinin family of peptides is one such example that has been repeatedly validated as playing a key role in the transmission of nociceptive signaling. Tachykinins interact with the three GPCRs NK_1_ (TACR1), NK_2_ (TACR2), and NK_3_ (TACR3), which selectively bind Substance P, neurokinin A, and neurokinin B, respectively. *Tacr1* has served as a persistent target in knockout models given that its ligand Substance P—an excitatory neurotransmitter involved in neurogenic inflammation and immune response (Donkin et al., 2007; Freidin & Kessler, 1991; Garza et al., 2008; Palma & Manzini, 1998; Rameshwar et al., 1992)—colocalizes with glutamate in primary afferents that respond to painful stimuli (de Felipe et al., 1998). To that end, TACR1‐deficient mice display reduced mechanical and thermal sensitivities to multiple inflammatory agents, including protease‐activated receptor 2 (PAR2) agonists and the TRPV1 agonist resiniferatoxin (de Felipe et al., 1998; Hunyady et al., 2019; Vergnolle et al., 2001). Perhaps unsurprisingly given the interplay between TACR1 and TRPV1, genetic disruption of TACR1 also reduces neuropathic sensitivity and reduces chemically‐induced visceral pain following the administration of capsaicin and acetic acid—the former being a well‐established agonist of TRPV1 (Hunyady et al., 2019; Laird et al., 2000; Mansikka et al., 2000).

GPCRs specific to many non‐neuronal ligands—such as the pro‐inflammatory peptide bradykinin—have also been targeted in murine knockdown models. Genetic disruption of the bradykinin receptors B1 (BDKRB1) and B2 (BDKRB2) decreases inflammatory sensitivity in a variety of disease‐specific models, including the multiple sclerosis model experimental autoimmune encephalomyelitides and a gout model induced by monosodium urate injection (Dutra et al., 2013; Silva et al., 2016). Both receptors have also been validated as playing key mediatory roles in more general inflammatory conditions induced by the local injection of formalin (mechanical and thermal), capsaicin (mechanical and thermal), carrageenan (mechanical only), CFA (thermal only), and the protein kinase C agonist phorbol myristate acetate (spontaneous pain‐associated behaviors; Ferreira et al., 2001, 2008; Pesquero et al., 2000; Rupniak et al., 1997). BDKRB1 has additionally been studied in relation to neuropathic pain, with a 2005 study has demonstrated that its deletion decreases mechanical and thermal sensitivity following partial nerve ligation (Ferreira et al., 2005). Taken together, these results lend credence to the possibility that BDKRB1 plays a role in the development of neuropathy and indicate that selective B1 receptor antagonists may hold broad therapeutic potential in the management of chronic pain.

Disruption of the adenosine receptors ADORA2A and ADORA3—both purinergic GPCRs involved in the downstream modulation of cyclic adenosine monophosphate (cAMP) levels—similarly decreases carrageenan‐induced thermal sensitivity and spontaneous formalin‐induced pain‐associated behaviors (Hussey et al., 2007; Ledent et al., 1997; W. P. Wu et al., 2002). While these results are not entirely unsurprising given that adenosine—like bradykinin—is a potent vasodilator, it is interesting that the administration of selective ADORA3 agonists alleviates neuropathic sensitivity following chronic constriction injury, spared nerve injury, and spinal nerve ligation as well as in a model of cancer‐induced bone pain (Durante et al., 2021; Little et al., 2015). In addition to suggesting that ADORA3 plays an anti‐nociceptive role in endogenous neuropathic pathways and a pro‐nociceptive role in inflammatory pathways, these results raise the possibility that the channels true potential may lie in an as‐of‐yet unrealized clinical application: given that ADORA3 agonists are being investigated as possible cancer therapeutics following from ADORA3's overexpression in cancer and inflammatory cell types, ADORA3's involvement in certain forms of cancer‐induced neuropathic pain may offer promising clinical advantages over currently available treatments for select inflammatory, ophthalmic, and liver diseases (Fishman et al., 2012; Little et al., 2015).

Despite being broadly implicated in signaling factors, there is much to be determined about specific GPCRs linked to pain. For instance, knockout studies into the adrenergic receptors ADRA2A and ADRB2—both of which bind catecholamines such as norepinephrine and epinephrine—have been mostly dedicated to one pain modality to the exclusion of broader analgesic considerations. While one study indicated that ADRA2A plays a pro‐nociceptive role in neuropathic thermal sensitivity, another showed a bifurcated chemical response to capsaicin depending on the method of delivery (Kingery et al., 2000; Mansikka et al., 2004). Other knockdown studies have sought to validate ADRA2A's role in mediating spinal analgesia. As might be expected, ADRA2A‐deficient mice display decreased analgesic response to the nonselective ADRA2 agonist UK 14304 and display increased analgesic response to morphine and to the partial opioid agonists buprenorphine and tramadol (Özdoǧan et al., 2006; Stone et al., 1997). While informative, the specificity of these studies has complicated efforts to draw global inferences on the relationships between specific genes and larger, more systemic nociceptive pathways.

### 3.3 Neuropeptides, neurotransmitters, and neurotrophins

Despite the prominence of membrane receptors in pain, their disruption represents only one method of attenuating the intercellular pathways involved in pain signaling. Disruption of endogenous ligands or cytosolic protein partners is a powerful alternative approach. To that end, neuropeptides and neurotransmitters have long served as recurrent targets of interest given their integral role as chemical messengers in the central and peripheral nervous system. Neuropeptides are synthesized from large, inactive precursor proteins called prepropeptides that are proteolytically cleaved and processed in the Golgi apparatus. Following the processing of the prepropeptide, the mature neuropeptide is packaged into dense‐core vesicles and transported throughout the neuron so that it may be released at the synaptic cleft, cell body, or axon. Upon their release, neuropeptides fulfill many functions, including posttranslational processing, the activation of transmembrane receptors such as ionotropic and metabotropic receptors, and the modulation of neural substrates. Together with low‐molecular‐weight neurotransmitters, they also facilitate the precise modulation of synaptic strength and neuronal plasticity (Russo, 2017). Research into their regulatory function thus holds an immense therapeutic potential given that neuropeptides can act as both transmitters and trophic factors. This broad analgesic promise is further substantiated by the fact that neuropeptides represent the largest and most diverse class of signaling molecules in the nervous system—with over 100 neuropeptides having been identified in the human genome alone and another 1000 having been predicted from genomic and transcriptomic data (Russo, 2017).

Whereas most neuropeptide receptors are GPCRs, neurotransmitters can bind either ionotropic or metabotropic receptors depending on their molecular affinity. Such is the case with the tachykinin peptides, which form one of the largest families of neuropeptides. Principally characterized by a common C‐terminal sequence, all tachykinins are derived from just three precursor proteins—preprotachykinin‐1 (TAC1), preprotachykinin‐2 (TAC2—TAC3 in humans), and preprotachykinin‐4 (TAC4)—that are post‐translationally processed to produce Substance P, neurokinin A, neurokinin K and neurokinin gamma (encoded by Tac1); neurokinin B (encoded by Tac2); and, hemokinin, endokinin A, and endokinin B (encoded by Tac4). Of the three genes responsible for coding the preprotachykinins, Tac1, in particular, has been subject to extensive study over the past two decades, with knockout models having validated TAC1 as playing a key role in mediating baseline sensitivity to acute mechanical and thermal stimulation (Cao et al., 1998; Zimmer et al., 1998). Multiple studies have also correlated TAC1 deficiency with decreased mechanical and thermal sensitivity following the administration of a wide range of chemical and inflammatory irritants (Cao et al., 1998; Hunyady et al., 2019; Sahbaie et al., 2009; Vergnolle et al., 2001; Zimmer et al., 1998). In addition to these phenotypes, TAC1‐deficient mice display decreased neuropathic mechanical and cold sensitivity following partial sciatic nerve ligation and decreased mechanical sensitivity following incision of the right hind paw (Hunyady et al., 2019; Sahbaie et al., 2009). These results show a certain continuity with Tacr1 knockout mice, which were previously discussed (de Felipe et al., 1998; Hunyady et al., 2019; Laird et al., 2000; Mansikka et al., 2000; Vergnolle et al., 2001). Nonetheless, some work conflicts with these findings. For instance, one study suggests that deletion of Tac1 fails to elicit a behavioral change following the injection of select chemical irritants, while another suggests that TAC1 deficiency increases visceral sensitivity to pH 4 saline (Lin et al., 2012; Zimmer et al., 1998). This latter result may be explained at least in part by the loss of Substance P's inhibitory effect given that Substance P has been linked to acid‐sensing ion channel 3 (ASIC3)‐positive neurons, which might cause TAC1‐deficient animals to manifest with a longer‐lasting hyperalgesia rather than with the transient hyperalgesia seen in control animals that possess intact ASIC3 signaling (Lin et al., 2012).

While TAC2 remains relatively understudied similar to what was seen with its receptor TACR2, TAC4 has been targeted in knockdown models. For instance, a 2019 study indicated that TAC4‐deficient mice display decreased somatic responses to both formalin and acetic acid injection, as well as decreased mechanical and thermal sensitivities following the injection of the pro‐inflammatory TRPV1 agonist resiniferatoxin (Hunyady et al., 2019).

Calcitonin gene‐related peptide (CGRP) has likewise garnered a great deal of clinical interest. CGRP exists in two isoforms derived from the alternate splicing of the calcitonin gene on chromosome 11, with Calca encoding alpha‐CGRP (CGRP I) and Calcb encoding beta‐CGRP (CGRP II). While alpha‐CGRP is predominantly expressed in the central and peripheral nervous system and has been extensively studied in relation to a variety of diseases—most notably migraine and cluster headache (Belin et al., 2020; Edvinsson, 2019)—beta‐CGRP is produced mainly in the enteric nervous system and remains comparatively understudied in pain (Muddhrry et al., 1988). Conditional knockout models have validated CALCA‐deficient animals as displaying decreased basal and inflammatory‐induced thermal sensitivity (McCoy et al., 2013; L. Zhang et al., 2001). However, *Calca* mutants developed normal neuropathic thresholds subsequent to spared nerve injury (McCoy et al., 2013). These results suggest that CALCA is involved in select nociceptive—but not neuropathic—signaling pathways. In line with this notion, deletion of *Calca* from TRPV1‐positive neurons reduced voluntary movement following the intraperitoneal injection of acetic acid, yet failed to provoke significant changes in spontaneous behaviors such as licking, grooming, and writhing compared to wild‐type controls (Spencer et al., 2018). Combined with the fact that other CALCA‐deficient animals showed reduced spontaneous responses to local capsaicin injection—a known agonist of TRPV1—it would appear that a molecular pathway exists that links expression of CALCA, Substance P, and TRPV1 in response to select nociceptive stimuli (McCoy et al., 2013). This hypothesis is consistent with the observation that alpha‐CGRP is needed for ATP‐induced thermal hyperalgesia following the proalgesic sensitization of TRPV1 in peptidergic nociceptors (Devesa et al., 2014).

Neurotrophic peptides—more commonly known as neurotrophins—are an important class of molecules that are implicated in the development and function of the nervous system. They comprise a subset of neurotrophic growth factors that activate the Trk family of receptor tyrosine kinases and p75NTR to initiate the mitogen‐activated protein kinase, phosphoinositide 3‐kinase, and c‐Jun N‐terminal kinase signaling cascades (Huang & Reichardt, 2001). Neurotrophins also affect neurotransmitter release at central and peripheral nervous system synapses through presynaptic mechanisms and facilitate the retrieval of synaptic vesicles after exocytosis (Tyler et al., 2002). While there are only four canonical neurotrophins in the human genome, approximately 50 growth factors have been identified in the mammalian nervous system as a whole.

Of the four mammalian neurotrophins, nerve growth factor (NGF) was the first to be characterized following its discovery in 1956 (Cohen & Levi‐Montalcini, 1956). In a foundational 1994 study, heterozygous NGF‐deficient animals generated from homologous recombination displayed marked deficits in baseline mechanosensation (Crowley et al., 1994). While homozygous mice exhibited an even stronger pain‐resistant phenotype, they also showed significant cell loss in the sympathetic ganglion as well as selective cell loss in sensory ganglia—specifically of small peptidergic neurons expressing CGRP and/or Substance P—and were not viable beyond the first few weeks of life. More recent studies that have capitalized on the increased specificity afforded by CRISPR systems have been more successful in bringing adult mice to viability. To that end, targeted deletion of NGF in the knee joint of adult mice alleviated mechanical hypersensitivity following partial meniscectomy (L. Zhao et al., 2019). However, these mice also showed worsened joint damage following surgical intervention. In an effort to counter this degeneration and preserve joint morphology, the researchers selectively deleted the cytokine interleukin 1 beta (IL‐1B) and the enzyme matrix metalloproteinase 13 (MMP13) using the same recombinant system, given that IL‐1B is upregulated in osteoarthritis and MMP13 is a dominant collagenase expressed in osteoarthritic cartilage. While individual deletion of either MMP13 or IL‐1B reduced expression of cartilage‐degrading enzymes and attenuated structural deterioration, joint deletion of both MMP13 and IL‐1B significantly mitigated the structural damage caused by localized NGF deficiency. Taken together, these results point to future avenues of osteoarthritic pharmaceutical development that could jointly address pain management and joint morphology through combined targeting of NGF, MMP13, and IL‐1B. In addition to these genetic models, local injection of NGF has been used as a key inflammatory mediator in behavioral models given that NGF serves as a potentiator of protein synthesis (Barragan‐Iglesias et al., 2021; Melemedjian et al., 2010; de la Peña, Barragan‐Iglesias, et al., 2021; de la Peña, Kunder, et al., 2021).

While conditional deletion of brain‐derived neurotropic factor (BDNF) from sensory neurons resulted in only minor changes in acute and chronic pain sensitivity, this response was sexually dimorphic—with only male mice having displayed decreased thermal sensitivity and decreased spontaneous formalin‐induced pain‐associated behaviors (Dembo et al., 2018). While female mice failed to show changes in nociceptive response, they interestingly displayed decreased histamine‐induced scratching. A subsequent study indicated that BDNF‐deficient heterozygous rats showed impairment of both A‐delta‐ and C‐fiber‐mediated thermal sensitivity as well as of C‐fiber‐mediated cold sensitivity (Sapio et al., 2019). This knockdown component was combined with a genome‐wide association study of BDNF haploinsufficiency in humans, which likewise correlated BDNF deficiency with decreased pain sensitivity. The fact that these results contradict those of the previous 2018 study in which conditional deletion of BDNF largely failed to elicit changes in basal nociceptive response—apart from a sexual dimorphic decrease in male thermal sensitivity—points to a critical need for further research into BDNF‐mediated nociceptive pathways.

### 3.4 Growth factors, hormones, and cytokines

While many of the genes that have been discussed up to this point in the review are predominantly found in neurons, the next series of factors is more broadly distributed. Growth factors, hormones, and cytokines often act on neurons in profound ways but are generated by non‐neuronal cell types—particularly immune cells. Despite their distinct biochemical and physiological characteristics, many overlap in function, which is why we have elected to discuss them in the same section. It is worth starting with a provisional disambiguation. In the broadest sense of the term, growth factors are small signaling glycoproteins that are involved in the regulation of cellular growth. While often thought of as inducing proliferation during the initial stages of neurogenesis and development, they also play key roles in cellular differentiation and intercellular signaling. In so doing, they can impact neuronal plasticity. While growth factors with a glandular origin may in all cases be considered hormones, not all hormones are peptide‐derived and thus not all hormones can be considered growth factors. A striking example is provided by steroid hormones, which are themselves derived from cholesterol. The more lax definition of hormones as signaling molecules that act distally from their site of production has contributed to a blurring of categorical distinction between hormones and cytokines—the latter of which constitute signaling molecules associated with immune response. Nonetheless, the fact that hormones are typically secreted by specific cell types whereas cytokines are produced by a wide range of cell populations—most notably helper T cells and macrophages—serves as one primary point of differentiation. The fact that hormones typically circulate in concentrations that vary by less than one order of magnitude while the expressed concentration of cytokines can increase dramatically following peripheral trauma or local infection serves as another major difference. Despite these categorical ambiguities, all three classes show a conserved importance in intercellular signaling as well as in the potentiation of neuronal responses to various forms of chronic pain.

We have already touched on the manners in which growth factors may be broadly implicated in neurogenesis and synaptic plasticity. To that end, our prior discussion of the neurotrophins NGF and BDNF may be applied to our molecular understanding of growth factors as a whole since neurotrophic factors are by their very definition a subclass of growth factors acting on neural substrates. Given their essential role in potentiating cellular development, the manipulation of growth factors serves as a conserved regulatory mechanism of nociceptive response across a wide variety of pain conditions. As stated before, this results in significant functional overlap with cytokines, which likewise regulate proliferation and differentiation in hematopoietic and immune cell types. For instance, colony‐stimulating factors 1 (CSF1) and 2 (CSF2) are commonly classified as cytokines, but also fit the broad taxonomic classification of growth factors. Both exemplify how disrupting the endogenous expression of growth factors precludes their ability to bind cell surface receptors and initiate downstream signaling cascades. Two recent studies showed that CSF1 deletion completely prevents the onset of mechanical hypersensitivity following spared nerve injury (Guan et al., 2015; Yu et al., 2020). Moreover, injection of CSF1 into wild‐type animals produces a characteristic form of mechanical hypersensitivity that is associated with microglia activation—thereby demonstrating that the disruption of CSF1 signaling plays an antinociceptive role in select models of neuropathic pain. This proalgesic effect of the CSF family was further substantiated in studies of CSF2 in which targeted disruption of the gene's locus increased inflammatory pain thresholds in the experimental autoimmune prostatitis model and increased neuropathic mechanical thresholds following partial sciatic nerve ligation (Liu et al., 2019; Nicol et al., 2018).

Genetic models have similarly validated hormones as holding a vast therapeutical potential given their involvement in a wide range of signaling pathways. Hormones can be divided into four main classes: endocrine hormones, which are released into the bloodstream to regulate distant targets; paracrine hormones, which are diffused locally to act on a proximal target; autocrine hormones, which bind receptors on the same cell from which they are produced; and, intracrine hormones, which are involved in intracellular signaling cascades in the cell in which they are synthesized. This provisional classification nonetheless poses a taxonomic problem as certain hormones can act through multiple pathways. For instance, the cytokine interleukin 6 (IL‐6), which may alternatively be classified as a hormone, has been shown to participate in all four types of chemical signaling (Alberti et al., 2004; Chiu et al., 1996; T. H. Jones, 1994; Sreenivasan et al., 2020). Perhaps a more useful means of distinction is thus based on molecular structure, which delineates hormones between amino acid‐derived hormones, peptide hormones, and lipid‐derived hormones. Such a perspective is illuminating in that it reveals a significant overlap between peptide hormones and neuropeptides, with the sole defining characteristic that differentiates neuropeptides from peptide hormones being that the former is synthesized by neurons to bind neural substrates.

Prominent polypeptide hormones that have been targeted in murine knockdown models include preproenkephalin 1 (PENK1) and prodynorphin (PDYN). Disruption of PENK1 increases anxiety‐related behaviors, decreases nicotine‐induced analgesia, and increases baseline thermal sensitivity (Berrendero et al., 2005; Bilkei‐Gorzo et al., 2004; König et al., 1996). These results suggest a broad analgesic effect of PENK1 while at the same time pointing to enkephalins as modulating supraspinal pain thresholds. PDYN has similarly been implicated in the chemical transduction of analgesic signaling, with its deletion having reduced mechanical and thermal sensitivities following nerve injury (Z. Wang et al., 2001). PDYN has also been linked to tetrahydrocannabinol‐induced analgesia, although conflicting behavioral results in spinal antinociceptive assays necessitate further experimentation (Gardell et al., 2002; Zimmer et al., 2001).

Perhaps the greatest potential for targeted hormone therapies lies in sexually dimorphic pain conditions given that an increasing number of studies suggest sexually dimorphic responses to select analgesic agents as well as differentiated mechanisms of pain sensitization and hyperalgesic priming (Ferrari et al., 2016; Gensel et al., 2019; Loyd & Murphy, 2014; Navratilova et al., 2021). For instance, surgical and pharmacological disruption of nuclear receptors/transcription factors estrogen receptor alpha (ESR1) and androgen receptor (AR) has helped elucidate the sexually dimorphic signaling pathways involved in IL‐23‐induced mechanical allodynia. Ovariectomy decreased IL‐23‐ and IL‐17A‐induced mechanical sensitivity, as did injection of female mice with the ESR1 antagonist MPP (X. Luo, Chen, et al., 2021). While male mice failed to show a similar inflammatory response to IL‐23 alone, the joint administration of the ESR1 agonist PPT with IL‐23 was successful in inducing a characteristic form of mechanical allodynia that resembled that which was seen in female littermates. To better understand the molecular mechanism behind this sexually dimorphic response, additional behavioral assays were performed in which orchiectomy increased IL‐23‐ and IL‐17A‐induced mechanical pain, as did the administration of the AR antagonist ailanthone in wild‐type male mice. Taken together, the authors surmised that estrogen promotes IL‐23‐mediated mechanical pain via IL‐17, while androgen suppresses IL‐23‐mediated pain.

As stated earlier, cytokines are globally defined as small secreted proteins that affect intercellular immune responses. They comprise a broad molecular class that includes lymphokines produced by lymphocytes, monokines produced by monocytes, chemokines having chemotactic activities, and interleukins that are produced by one leukocyte to act on others. Of these, interleukins have been of particular interest in pain. The widespread therapeutic potential of cytokines not only arises from their key role in immune response, but also from their particular mechanisms of action. As an example, cytokines are redundant and are often produced in cascades through which they can act synergistically or antagonistically (Ozaktay et al., 2006; J.‐M. Zhang & An, 2007). They can also be transported from the periphery via both axonal and nonaxonal mechanisms.

A great deal of research has coalesced around proinflammatory cytokines given their potentiating effects in chronic pain conditions. For instance, interleukin 1 (IL‐1) has been validated as a key regulator of inflammatory and neuropathic pain. Double knockout of interleukin 1 alpha (IL‐1A) and interleukin 1 beta (IL‐1B) decreased CFA‐ and formalin‐induced mechanical and thermal sensitivities, as well as neuropathic mechanical sensitivity following nerve injury (Honore et al., 2006). These results were recapitulated by studies into interleukin 1 receptor type 1 (Il1r1)—the transmembrane receptor for IL‐1A, IL‐1B, and interleukin 1 receptor antagonist (IL‐1RA)—in which Il1r1‐deficient animals displayed decreased knee bending allodynia following administration of recombinant IL‐1B into the sciatic nerve, and decreased mechanical hypersensitivity following spared nerve injury (Gui et al., 2016; Mailhot et al., 2020).

Interleukin 6 (Il6) is another prominent pain gene. IL‐6‐deficient mice display decreased mechanical and thermal sensitivity following partial sciatic nerve ligation, increased autonomy (self‐mutilation), and delayed recovery from crushed nerve injury as seen through behavioral footprint analysis, and delayed onset of mechanical allodynia following sciatic nerve ligation (Imai et al., 2013; Murphy et al., 1999; Ramer et al., 1998; Xu et al., 1997; Zhong et al., 1999). IL‐6 deficiency has also been correlated with decreased carrageenan‐induced mechanical sensitivity (Xu et al., 1997; Zhong et al., 1999). IL‐6's deletion has also been associated with decreased thermal analgesia following the administration of morphine, which suggests that IL‐6‐signaling is necessary for the development of neuronal mechanisms involved in endogenous and exogenous opiate response (Bianchi et al., 1999).

Similar to hormones, certain cytokines have exhibited sexually dimorphic phenotypes. For instance, deletion of interleukin 23 (IL‐23) decreased early and late phase paclitaxel‐induced mechanical sensitivity in female mice despite male mice failing to develop a statistically significant change in neuropathic response (X. Luo, Chen, et al., 2021). Administration of IL‐23 similarly increased inflammatory‐induced mechanical sensitivity in female mice, yet failed to elicit a similar response in males. These results were broadly reproduced by the deletion of interleukin 23 receptor (IL‐23R) in both male and female mice; however, only females showed decreased levels of paclitaxel‐induced and IL‐23‐induced mechanical sensitivity. Administration of the IL‐23R antagonist P2305 similarly decreased IL‐23‐induced mechanical sensitivity and decreased mechanical allodynia arising from three separate forms of neuropathy. IL‐23R blockade also decreased spontaneous formalin‐induced responses in a sexually dimorphic manner, with only female mice showing significant changes in pain‐associated behaviors. Taken together, these results indicate that IL‐23 drives mechanical pain through IL‐23R in female mice and point to the hypothesis that IL‐23 and IL‐23R are required for female‐specific neuropathic mechanical allodynia and spontaneous inflammatory pain.

### 3.5 Enzymes and enzyme‐linked receptors

Given their central involvement in biochemical signaling pathways, enzymes and enzyme‐linked receptors play prominent roles in pain. They serve as particularly tempting analgesic targets for several reasons. As catalysts that accelerate biochemical reactions, they exhibit a high level of specificity with their associated substrate and therein allow for the precise manipulation of biological processes. For example, the disruption of protein kinases and phosphatases provides a powerful means of post‐translationally regulating gene expression, while the manipulation of secondary messengers targeted by activated G proteins allows for the attenuation of intracellular signaling cascades. The targeting of ubiquitin‐activating and ubiquitin‐conjugating enzymes similarly allows for the control of protein degradation. Enzymes represent an exceedingly large and diverse functional class, with recent supplements to the Enzyme Commission Report inventorying over 5000 different enzymes across six classes based on reaction type (Robinson, 2015). Accordingly, nearly a quarter of the genes inventoried in our analyses have a catalytic function.

The three subfamilies of the mitogen‐activated protein kinases (MAPKs)—the extracellular signal‐regulated kinases (ERKs), p38 mitogen‐activated protein kinases, and c‐Jun N‐terminal kinases (JNKs)—have all been targeted in knockdown models over the past two decades. Starting with the ERKs, deletion of MAPK3 delays the onset of long‐term formalin‐induced thermal sensitivity, yet fails to alter mechanosensation (Alter et al., 2010). MAPK3‐deficient animals additionally show no change in CFA‐induced mechanical and thermal sensitivities, nor are their neuropathic thresholds altered following spared nerve injury. While these negative results contradict other studies that have validated Erk1/2 signaling as being necessary for nociceptive sensitization (Ji et al., 1999, 2002; Karim et al., 2006; Karim, Wang, & Gereau 4th, 2001; Seino et al., 2006; Song et al., 2005), this difference may be due to a differential involvement of Erk1/2 isoforms in pain processing. To that end, MAPK1‐deficient animals display decreased formalin‐induced nocifensive behaviors and decreased allodynic—but not hyperalgesic—mechanical sensitivity following partial sciatic nerve ligation (Otsubo et al., 2012). This is in contrast to the lack of phenotypes observed in MAPK3‐deficient mice. While MAPK1‐deficient mice show a transient increase in spontaneous reactions during the first phase of formalin injection, these animals show an overall decrease in nocifensive behaviors over the course of the experiment, which would seem to suggest a more complex role of Erk2 in formalin‐induced peripheral sensitization (O'Brien et al., 2015). This hypothesis is supported by the fact that MAPK1‐ and MAPK3‐deficient mice showed divergent responses to NGF‐induced thermal sensitivity—with MAPK3 disruption playing a pro‐nociceptive role and MAPK1‐deficiency attenuating nociceptive responses. Given that MAPK3 deletion increases basal Erk2 (encoded by Mapk1) phosphorylation while largely failing to elicit a significant nociceptive response, it would thus appear that Erk1 (encoded by Mapk3) plays only a limited role in nociceptive processing compared to Erk2 (Alter et al., 2010).

In an effort to better understand the interplay between Erk1 and Erk2 expression in pain, a double knockout strain was generated in which MAPK1 and MAPK3 were selectively deleted from Na_v_1.8‐positive sensory neurons (O'Brien et al., 2015). While these mice showed vast sensory deficits, they also developed a marked increase in autonomy (self‐mutilation) and—as a result—further behavioral tests were aborted. The authors thus turned to cell culture experiments in an attempt to better understand the Erk1/2 signaling axis. These experiments demonstrated that at least one functional Erk isoform was necessary for successful target innervation and for the survival of Na_v_1.8‐positive sensory neurons—thereby substantiating that Erk1 and Erk2 exhibit functionally distinct, yet redundant, roles in the function and maintenance of sensory neurons.

While substantial research has been conducted into p38 involvement in stressor‐related signaling in non‐neuronal cells, p38 function in neurons and cognitive processes remains poorly understood. As such, while there exist four p38 isoforms—encoded by Mapk11 (p38‐beta), Mapk12 (p38‐gamma), Mapk13 (p38‐delta), and Mapk14 (p38‐alpha)—characterization of p38 isoforms in pain remains far more tenuous than with ERKs. Nonetheless, selective deletion of MAPK14 in CD90‐expressing neurons altered anxiety responses and led to activation of JNK, which suggests that p38‐alpha might be involved in nociceptive processing (Stefanoska et al., 2018). Given that p38‐alpha signaling has also been linked to astrocyte immune activation across multiple cell types, MAPK14 regulation could possibly be exploited in various models of inflammatory pain (Lo et al., 2014). Despite these preliminary findings, the development of p38 inhibitors has been hindered by their significant toxicity, which has led to the exploration of alternative strategies for p38 regulation. One approach has been to target upstream activators of p38, such as mitogen‐activated kinase kinase 3 (MKK3) and 6 (MKK6; Sorkin et al., 2009). While neither MKK3‐ nor MKK6‐deficient mice develop significant changes in acute formalin‐induced behaviors, MKK3 mice show delayed onset of formalin‐induced mechanical allodynia compared to MKK6 mice and wild‐type controls. When taken together with the fact that MKK3‐deficient mice display both delayed and reduced phosphorylation of spinal p38, this suggests that MKK3 is necessary for the development of chronic pain.

Contrary to p38 kinases, the last of the three MAPK subfamilies—JNKs—have been explicitly validated as playing key roles in the onset of multiple forms of chronic pain. A 2010 study into Jnk1 (encoded by Mapk8) and Jnk2 (encoded by Mapk9) showed a differential involvement of the two isoforms in CFA‐induced pain (Gao et al., 2010). While MAPK8‐deficient mice display attenuated CFA‐induced mechanical—but not thermal—sensitivity, MAPK9‐deficient mice fail to develop behavioral changes compared to wild‐type controls. Despite this divergent inflammatory response, both genotypes show no difference in basal nociceptive thresholds—thereby suggesting that neither Jnk1 nor Jnk2 plays a significant role in pain sensitivity under normal conditions.

JNK involvement in endogenous pain pathways was further substantiated by a joint study into Jnk1, Jnk2, and Jnk3 (encoded by Mapk10), which sought to understand how the three isoforms contributed to neuropathic pain and autonomy following nerve injury (Manassero et al., 2012). While individual knockouts of all three enzymes decreased neuropathic mechanical sensitivity following sciatic nerve transection, only MAPK8‐ and MAPK10‐deficient animals showed reduced autonomy compared to wild‐type controls. In an effort to mimic a triple knockout mouse model of all three isoforms, the authors injected wild‐type mice with the highly selective JNK inhibitor D‐JNKI‐1 prior to inflicting nerve injury. While this decreased neuropathic mechanical sensitivity as would be expected, the mice showed divergent responses in autonomy according to dosage protocol: while a single injection of D‐JNKI‐1 resulted in high autonomy, as was the case with both wild‐type controls and individual deletion of MAPK9, multiple injections resulted in animals displaying no autonomy, as was the case with individual deletions of MAPK8 and MAPK10. This implied a cumulative effect of JNK inhibition and reproduced the results of previous pharmacological studies that similarly indicated that neuropathic pain develops once peptide infusion is terminated (Zhuang et al., 2006). Taken together, these results suggest that all three JNK isoforms collectively contribute to maintain neuropathy, albeit through different mechanisms: while inhibition of all three isoforms prevents the onset of neuropathic pain, deletion of single splice variants appears to mitigate gross sensory abnormalities resulting from peripheral nerve injury. As such, JNK‐targeted pharmaceuticals serve as a promising avenue of future research into the treatment of neuropathic pain resulting from surgical nerve damage.

Other kinases are known to interact with the MAPKs—such as the mitogen‐activated protein kinase‐interacting kinases 1 (MNK1) and 2 (MNK2)—have also been validated in murine knockdown models. Deletion of MNK1 and MNK2, as well as the administration of the MNK1/2 inhibitor cercosporamide, resulted in significantly faster recovery from CFA‐induced mechanical and thermal hypersensitivity, decreased mechanical priming following injection of prostaglandin E_2_, and decreased neuropathic mechanical and cold sensitivity following spared nerve injury (Moy et al., 2017). This validation serves to broadly characterize the MNK‐eIF4E signaling axis as an important contributing factor to nociceptive plasticity and points to its central role in the development of chronic pain states.

While our review has largely focused on kinases given their prominent role in post‐translational regulation, many other enzymes have been validated as playing key roles in nociceptive signaling. Of particular note is the cytoplasmic ribonuclease Dicer that facilitates activation of the RNA‐induced silencing complex (RISC), which is itself the effector nuclease for RNA interference (Hammond et al., 2000). While selective deletion of DICER1 from adult sensory neurons reduced sensitivity to noxious cold stimulation, this may have been due to lower levels of SCN10A expression, given that the conditional knockout model used a Na_v_1.8‐Cre recombinant system (J. Zhao et al., 2010). Behavioral assays into inflammatory response were arguably more informative. DICER1 deletion decreased formalin‐ (spontaneous), carrageenan‐ (thermal), and CFA‐induced (mechanical and thermal) inflammatory response; however, these mice showed no change in neuropathic sensitivity following partial sciatic nerve ligation. Of note, these results reproduced those of previous studies into Na_v_1.8‐mediated inflammatory sensitivity and validated that the potentiation of neuropathic pain does not require Na_v_1.8‐positive sensory neurons (Abrahamsen et al., 2008; Akopian et al., 1999; Stirling et al., 2005). In an effort to better understand the mechanism by way of which DICER1 disruption affects peripheral sensitization, immunohistochemical assays were performed subsequent to formalin injection. These studies demonstrated that the number of c‐Fos‐immunoreactive neurons was reduced by over 50% compared to wild‐type controls. Moreover, cultured neurons showed normal activation thresholds following the addition of inflammatory mediators, despite wild‐type neurons showing a significant increase in postsynaptic firing.

While specific enzymes have long served as recurrent targets in pain, so too have their associated receptors. Enzyme‐linked receptors transduce signals through kinases and can be broadly divided into three categories. In the first, the enzyme forms an intrinsic part of the receptor, such as is the case with tyrosine and serine/threonine receptor kinases. In the latter two, the enzyme is bound directly or indirectly to the receptor via adaptor proteins, such as is the case with cytokine receptors. In addition to IL‐1R and IL‐23R which were discussed in the previous section, tropomyosin receptor kinase A (NTRK1) and toll‐like receptor 4 (TLR4) have been implicated in pain signaling. NTRK1 deletion decreases mechanical and thermal baseline sensitivities, decreases CFA‐induced inflammatory sensitivity, and increases neuropathic autonomy following nerve injury (Smeyne et al., 1994). TLR4 similarly mediates select forms of inflammatory and neuropathic sensitivity (T. T. Hu et al., 2018; Wardill et al., 2016). It also shows a sexually dimorphic involvement in mast cell‐mediated migraine, with only male mice showing reduced 40/80‐induced light sensitivity (Ramachandran et al., 2019).

### 3.6 Transcriptional and translational control and mRNA processing

Transcriptional control is an important mechanism that regulates the amount of pre‐mRNA synthesized at a given moment in time. Once generated, the pre‐mRNA must be processed to form a fully mature mRNA. These changes can affect downstream translation by altering transcript stability or the composition of the polypeptide that results from translation. While these changes can indirectly affect nascent translation, translation itself may also be directly targeted through various initiation factors and translational repressors. Pharmacology directed against the translational apparatus and factors that control mRNA has grown considerably over the past two decades, with the FDA having approved multiple nucleic acid‐based therapeutics (e.g., Macugen, Nusinersen, Formivirsen, Inotersen, Eteplirsen, Golodirsen, Viltolarsen, Casimersen, mRNA‐1273, etc.; de la Peña & Campbell, 2018; Shukla et al., 2020).

Numerous transcription factors have been examined in pain signaling. Of these, PR homology domain‐containing member 12 (PRDM12) is of particular interest given that its disruption has been linked to human congenital insensitivity to pain (CIP; Y.‐C. Chen et al., 2015). This association was genetically validated in Drosophila when RNAi knockdown of the Drosophila PR homolog Hamlet reduced nociceptive responses to thermal stimuli (Nagy et al., 2015). PRDM12 function was subsequently assayed in mammalian model systems. While germline deletion of PRDM12 and conditional knockout of PRDM12 in the embryonic neural crest was neonatal lethal, conditional knockout of PRDM12 in embryonic DRGs produced viable offspring (Kokotović et al., 2021; Landy et al., 2021). These mice displayed increased mortality and exhibited corneal abrasions and facial scarring similar to what is seen in human CIP patients; they also displayed decreased baseline sensitivity to mechanical and cold stimulation, decreased chemical sensitivity to capsaicin injection, and decreased itch response to chloroquine and histamine. While baseline thermal thresholds were unaffected in the embryonic conditional knockouts, the authors attributed this to a compensatory elevation of TRPM3 levels that were observed in qRT‐PCR. Despite the embryonic knockout model producing a significant pain phenotype, adult knockout models largely fail to show changes in pain sensation. This indicates that PRDM12's function changes over development—playing a key role in embryonic nociceptor neurogenesis and acting as a transcriptional activator in adults.

Runt‐related transcription factor 1 (RUNX1) is a key mediator of nociception that targets multiple ion channels and GPCRs. While expressed in a wide variety of nociceptors during embryonic development, RUNX1 is largely restricted to cells that co‐express the neurotrophin receptor RET in the mature nervous system. Given that RUNX1 is necessary for normal development, homozygous null mutations show lethality at 1.5 weeks following the development of fetal liver hematopoiesis or hemorrhaging in the central nervous system (Okuda et al., 1996; Q. Wang et al., 1996). Despite the failure of whole‐body knockout models to display a clinically relevant phenotype, conditional knockout of RUNX1 produces viable offspring and increases baseline nociceptive thresholds to noxious thermal stimuli and the cold mimetic acetone (C.‐L. Chen et al., 2006). In addition, RUNX1‐deficient animals display decreased spontaneous reactions to capsaicin injection, reduced CFA‐induced mechanical sensitivity, and reduced neuropathic mechanical sensitivity following spared nerve injury.

Activating transcription factor 4 (ATF4)—which facilitates response to extracellular signals to maintain homeostasis and plays a key role in the integrated stress response—mediates thermal sensitivity in a variety of inflammatory and neuropathic models (Hai & Hartman, 2001; Pakos‐Zebrucka et al., 2016; Vasudevan et al., 2020). A joint siRNA knockdown and knockout study indicated that ATF4‐deficient animals display reduced baseline, inflammatory, and neuropathic thermal sensitivities, yet fail to show changes in mechanical thresholds (M.‐X. Xie, Cao, et al., 2021). These results suggest that ATF4‐mediated transcription might affect transcripts involved in thermosensation, but not mechanosensation. Interestingly, null‐mutants manifested with decreased mechanical and thermal responses to TRPM3 agonists, which the authors interpreted as suggesting molecular cross‐talk between ATF4 and TRPM3. This hypothesis was substantiated when immunohistochemistry validated ATF4 as contributing to kinesis‐mediated TRPM3 trafficking in DRG neurons.

While the above two factors play key roles in transcription initiation, transcriptional repressors have also been validated as playing key roles in nociceptive sensitization. For instance, conditional knockout of repressor element 1‐silencing transcription factor (REST)—which is involved in the repression of neuronal genes in non‐neuronal cells (Chong et al., 1995; Coulson, 2005)—completely prevents the onset of CFA‐induced mechanical, thermal, and cold hypersensitivity (F. Zhang et al., 2019). Moreover, REST‐deficient animals display decreased neuropathic mechanical sensitivity following spared nerve injury and partial sciatic nerve ligation—thereby substantiating that REST drives the development of multiple pain states.

While transcription serves as one method of capitalizing on RNA expression to regulate the perception of pain, factors affecting translation provide an alternative approach. One prominent example is eukaryotic translation initiation factor 2‐alpha (eIF2S1), which promotes the binding of the initiator tRNA to 40S ribosomal subunits to initiate translation. Given that systemic deletion of eIF2S1 is embryonic lethal, a 2016 study selectively mutated serine‐51 to a non‐phosphorylatable alanine residue in one allele of the gene (Khoutorsky et al., 2015). This functionally served as a knockout model given that basal eIF2‐alpha phosphorylation was reduced by approximately 50% compared to wild‐type control. While this disruption decreased basal thermal sensitivity and spontaneous nocifensive responses in the late/tonic phase of formalin injection, no changes were seen in basal mechanical or cold thresholds. Similarly, injection of the eIF2‐alpha phosphatase complex inhibitor Sal003 (a functional agonist of eIF2‐alpha) increased basal thermal sensitivity, while injection of the eIF2‐alpha kinase inhibitor PKRi (a functional antagonist of eIF2‐alpha) decreased basal thermal sensitivity.

Other eukaryotic translation initiation factors have been targeted in knockout models. For instance, genetic disruption of eukaryotic translation initiation factor 4E (eIF4E) decreases mechanical and thermal sensitivities to a wide range of inflammatory mediators (Moy et al., 2017). These animals also show reduced sensitivity following the injection of prostaglandin E_2_ to induce a model of hyperalgesic priming. This suggests that phosphorylation of the 5′ cap‐binding protein eIF4E by MNK1/2 plays an important contributing role in peripheral sensitization and the neuroplasticity of pain. Given that activation of mechanistic/mammalian target of rapamycin (mTOR) kinase has been validated as mediating both translation and hyperalgesia, other studies have sought to define the nociceptive role of eukaryotic translation initiation factor 4E‐binding protein 1 (eIF4EBP1)—itself a translational repressor and downstream mTOR effector. While eIF4EBP1 disruption increases basal mechanical sensitivity and spontaneous formalin‐induced pain‐associated behaviors, basal thermal thresholds remain unchanged (Khoutorsky et al., 2015). However, deletion of eIF4EBP1 enhances spinal cord expression of neuroligin 1 (NLGN1)—a postsynaptic protein involved in regulating excitatory synapse function—and results in cells showing lowered activation thresholds for inducing synaptic potentiation. Given that pharmacological inhibition of eIF4E and genetic ablation of NLGN1 normalizes this excitatory synaptic activity and reverses the observed mechanical hypersensitivity, the authors concluded that translational control by eIF4EBP1 affects expression of Nlgn1 and acts to modulate the excitatory synaptic transmission and nociceptive mechanosensation.

## 4 CONCLUSION

Advances in pain neurobiology have led to tremendous advances in our understanding of the genetic contributions to pain. Yet, the precision of these tools remains a critical consideration. In many cases, the use of whole‐body mutants results in ambiguity regarding the relevant cellular source of a given factor. The use of genetic tools that enable selective deletion of genes is ideal in this respect and has played an increasingly common role in behavioral and molecular research. As the ease of genome modification with tools such as CRISPR increases, it is likely that our understanding of disease mutants, functional portions of genes, and regulatory elements will continue to grow. The use of model organisms is essential to this endeavor given that pain requires a complete circuit that is not easily recapitulated in vitro. Moreover, there is tremendous value in comparing pain phenotypes across a range of organisms as conservation is often indicative of important biological functions.

Large datasets have emerged in a broad array of pain models. This includes genome‐wide measurements of mRNA levels in tissues and individual cells (K. D. Jones et al., 2016; Kupari et al., 2021; Ray et al., 2018; Usoskin et al., 2014; Uttam et al., 2018). Given the discordance between mRNA levels and translational output, there is growing interest in the use of methods that analyze translational efficiency as a means of precisely quantifying protein synthesis rates in response to painful stimuli (Barragan‐Iglesias et al., 2021; Megat, Ray, Moy, et al., 2019; Megat, Ray, Tavares‐Ferreira, et al., 2019; de la Peña, Barragan‐Iglesias, et al., 2021). Gene disruption provides an essential means of testing hypotheses that result from these powerful reverse genetic approaches. Understanding which genes matter in which sex and under what conditions may result in improved biomarkers and more efficient identification of analgesic targets. This information is invaluable for understanding the complex array of interactions required for pain‐associated behaviors.

Future research into the genetics of pain should incorporate powerful new screening approaches. For example, numerous strategies have been reported for the generation of sensory neurons with properties similar to nociceptors from human‐induced pluripotent stem cells (Blanchard et al., 2015; Chambers et al., 2012; Wainger et al., 2015). Similarly, CRISPR screens that make use of single defined guides have been tremendously useful in the identification of regulatory factors in a broad assortment of biological contexts (Replogle et al., 2020). The union of high‐throughput electrophysiology in the form of multi‐electrode arrays could likewise enable tremendous advances in our understanding of sensory neurons. Nonetheless, a critical limitation of this approach is that pain requires the interaction of numerous cell types spread across multiple tissues. The fact that behavioral responses require an intact circuit suggests that validation in model systems with relevant behavioral outputs is likely to remain the gold standard for the foreseeable future.

## AUTHOR CONTRIBUTIONS

**Eric Wistrom:** Conceptualization (equal); data curation (equal); investigation (lead); methodology (equal); visualization (supporting); writing – original draft (lead); writing – review and editing (equal). **Rebecca Chase:** Data curation (supporting); formal analysis (lead); investigation (supporting); methodology (supporting); validation (lead); visualization (equal). **Patrick Smith:** Visualization (equal). **Zachary Campbell:** Conceptualization (equal); data curation (equal); funding acquisition (lead); methodology (equal); supervision (lead); writing – review and editing (equal).

## FUNDING INFORMATION

This work was supported by NIH grants R01NS100788 (ZTC) and R01NS114018 (ZTC).

## CONFLICT OF INTEREST

The authors declare no competing interests.

## RELATED WIREs ARTICLE

RNA control in pain: Blame it on the messenger

## Supporting information


**Table S1** A compendium of validated pain genes.

## Data Availability

Data sharing is not applicable to this article as no new data were created or analyzed in this study

## References

[wsbm1570-bib-0001] Abrahamsen, B. , Zhao, J. , Asante, C. O. , Cendan, C. M. , Marsh, S. , Martinez‐Barbera, J. P. , Nassar, M. A. , Dickenson, A. H. , & Wood, J. N. (2008). The cell and molecular basis of mechanical, cold, and inflammatory pain. Science, 321(5889), 702–705. 10.1126/SCIENCE.1156916 18669863

[wsbm1570-bib-0002] Agarwal, N. , Offermanns, S. , & Kuner, R. (2004). Conditional gene deletion in primary nociceptive neurons of trigeminal ganglia and dorsal root ganglia. Genesis, 38(3), 122–129. 10.1002/GENE.20010 15048809

[wsbm1570-bib-0003] Ahmad, A. H. , & Ismail, Z. (2002). C‐fos and its consequences in pain. The Malaysian Journal of Medical Sciences, 9(1), 3. 22969311 PMC3436108

[wsbm1570-bib-0004] Akopian, A. N. , Souslova, V. , England, S. , Okuse, K. , Ogata, N. , Ure, J. , Smith, A. , Kerr, B. J. , McMahon, S. B. , Boyce, S. , Hill, R. , Stanfa, L. C. , Dickenson, A. H. , & Wood, J. N. (1999). The tetrodotoxin‐resistant sodium channel SNS has a specialized function in pain pathways. Nature Neuroscience, 2(6), 541–548. 10448219 10.1038/9195

[wsbm1570-bib-0005] Alawi, K. M. , Russell, F. A. , Aubdool, A. A. , Srivastava, S. , Riffo‐Vasquez, Y. , Baldissera, L. , Thakore, P. , Saleque, N. , Fernandes, E. S. , Walsh, D. A. , & Brain, S. D. (2017). Transient receptor potential canonical 5 (TRPC5) protects against pain and vascular inflammation in arthritis and joint inflammation. Annals of the Rheumatic Diseases, 76(1), 252–260. 10.1136/ANNRHEUMDIS-2015-208886 27165180 PMC5264234

[wsbm1570-bib-0006] Alberti, L. , Thomachot, M. C. , Bachelot, T. , Menetrier‐Caux, C. , Puisieux, I. , & Blay, J. Y. (2004). IL‐6 as an intracrine growth factor for renal carcinoma cell lines. International Journal of Cancer, 111(5), 653–661. 10.1002/IJC.20287 15252833

[wsbm1570-bib-0007] Alloui, A. , Zimmermann, K. , Mamet, J. , Duprat, F. , Noël, J. , Chemin, J. , Guy, N. , Blondeau, N. , Voilley, N. , Rubat‐Coudert, C. , Borsotto, M. , Romey, G. , Heurteaux, C. , Reeh, P. , Eschalier, A. , & Lazdunski, M. (2006). TREK‐1, a K^+^ channel involved in polymodal pain perception. The EMBO Journal, 25(11), 2368–2376. 10.1038/SJ.EMBOJ.7601116 16675954 PMC1478167

[wsbm1570-bib-0008] Alter, B. J. , Zhao, C. , Karim, F. , Landreth, G. E. , & Gereau, R. W. (2010). Genetic targeting of ERK1 suggests a predominant role for ERK2 in murine pain models. Journal of Neuroscience, 30(34), 11537–11547. 10.1523/JNEUROSCI.6103-09.2010 20739576 PMC2932641

[wsbm1570-bib-0009] Amaya, F. , Shimosato, G. , Kawasaki, Y. , Hashimoto, S. , Tanaka, Y. , Ji, R. R. , & Tanaka, M. (2006). Induction of CB1 cannabinoid receptor by inflammation in primary afferent neurons facilitates antihyperalgesic effect of peripheral CB1 agonist. Pain, 124(1–2), 175–183. 10.1016/J.PAIN.2006.04.001 16709443

[wsbm1570-bib-0010] Amaya, F. , Wang, H. , Costigan, M. , Allchorne, A. J. , Hatcher, J. P. , Egerton, J. , Stean, T. , Morisset, V. , Grose, D. , Gunthorpe, M. J. , Chessell, I. P. , Tate, S. , Green, P. J. , & Woolf, C. J. (2006). The voltage‐gated Sodium Channel Nav1.9 is an effector of peripheral inflammatory pain hypersensitivity. Journal of Neuroscience, 26(50), 12852–12860. 10.1523/JNEUROSCI.4015-06.2006 17167076 PMC6674969

[wsbm1570-bib-0011] Bagal, S. K. , Chapman, M. L. , Marron, B. E. , Prime, R. , Storer, R. I. , & Swain, N. A. (2014). Recent progress in sodium channel modulators for pain. Bioorganic & Medicinal Chemistry Letters, 24(16), 3690–3699. 10.1016/J.BMCL.2014.06.038 25060923

[wsbm1570-bib-0012] Bandell, M. , Dubin, A. E. , Petrus, M. J. , Orth, A. , Mathur, J. , Hwang, S. W. , & Patapoutian, A. (2006). High‐throughput random mutagenesis screen reveals TRPM8 residues specifically required for activation by menthol. Nature Neuroscience, 9(4), 493–500. 10.1038/nn1665 16520735

[wsbm1570-bib-0013] Banerjee, A. , Larsen, R. S. , Philpot, B. D. , & Paulsen, O. (2016). Roles of presynaptic NMDA receptors in neurotransmission and plasticity. Trends in Neurosciences, 39(1), 26. 10.1016/J.TINS.2015.11.001 26726120 PMC4716805

[wsbm1570-bib-0014] Barragan‐Iglesias, P. , Kunder, N. , Wanghzou, A. , Black, B. , Ray, P. R. , Lou, T. F. , Bryan de la Peña, J. , Atmaramani, R. , Shukla, T. , Pancrazio, J. J. , Price, T. J. , & Campbell, Z. T. (2021). A peptide encoded within a 5′ untranslated region promotes pain sensitization in mice. Pain, 162(6), 1864–1875. 10.1097/J.PAIN.0000000000002191 33449506 PMC8119312

[wsbm1570-bib-0015] Basbaum, A. I. , Bautista, D. M. , Scherrer, G. , & Julius, D. (2009). Cellular and molecular mechanisms of pain. Cell, 139(2), 267. 10.1016/J.CELL.2009.09.028 19837031 PMC2852643

[wsbm1570-bib-0016] Bautista, D. M. , Jordt, S. E. , Nikai, T. , Tsuruda, P. R. , Read, A. J. , Poblete, J. , Yamoah, E. N. , Basbaum, A. I. , & Julius, D. (2006). TRPA1 mediates the inflammatory actions of environmental irritants and proalgesic agents. Cell, 124(6), 1269–1282. 10.1016/J.CELL.2006.02.023 16564016

[wsbm1570-bib-0017] Bautista, D. M. , Siemens, J. , Glazer, J. M. , Tsuruda, P. R. , Basbaum, A. I. , Stucky, C. L. , Jordt, S.‐E. , & Julius, D. (2007). The menthol receptor TRPM8 is the principal detector of environmental cold. Nature, 448(7150), 204–208. 10.1038/nature05910 17538622

[wsbm1570-bib-0018] Bean, B. P. (2007). The action potential in mammalian central neurons. Nature Reviews Neuroscience, 8(6), 451–465. 10.1038/nrn2148 17514198

[wsbm1570-bib-0019] Belin, A. C. , Ran, C. , & Edvinsson, L. (2020). Calcitonin gene‐related peptide (CGRP) and cluster headache. Brain Sciences, 10(1), 1–16. 10.3390/BRAINSCI10010030 PMC7016902 31935868

[wsbm1570-bib-0020] Benke, D. (2020). GABA B receptors and pain. Current Topics in Behavioral Neurosciences, 52, 213–240. 10.1007/7854_2020_130 32812203

[wsbm1570-bib-0021] Berrendero, F. , Mendizábal, V. , Robledo, P. , Galeote, L. , Bilkei‐Gorzo, A. , Zimmer, A. , & Maldonado, R. (2005). Nicotine‐induced Antinociception, rewarding effects, and physical dependence are decreased in mice lacking the Preproenkephalin gene. Journal of Neuroscience, 25(5), 1103–1112. 10.1523/JNEUROSCI.3008-04.2005 15689546 PMC6725961

[wsbm1570-bib-0022] Bhave, G. , Karim, F. , Carlton, S. M. , & Gereau, R. W., IV . (2001). Peripheral group I metabotropic glutamate receptors modulate nociception in mice. Nature Neuroscience, 4(4), 417–423. 10.1038/86075 11276233

[wsbm1570-bib-0023] Bianchi, M. , Maggi, R. , Pimpinelli, F. , Rubino, T. , Parolaro, D. , Poli, V. , Ciliberto, G. , Panerai, A. E. , & Sacerdote, P. (1999). Presence of a reduced opioid response in interleukin‐6 knock out mice. European Journal of Neuroscience, 11(5), 1501–1507. 10.1046/J.1460-9568.1999.00563.X 10215902

[wsbm1570-bib-0024] Bilkei‐Gorzo, A. , Racz, I. , Michel, K. , Zimmer, A. , Klingmüller, D. , & Zimmer, A. (2004). Behavioral phenotype of pre‐proenkephalin‐deficient mice on diverse congenic backgrounds. Psychopharmacology, 176(3), 343–352. 10.1007/S00213-004-1904-9 15197532

[wsbm1570-bib-0025] Blanchard, J. W. , Eade, K. T. , Szűcs, A. , Sardo, V. l. , Tsunemoto, R. K. , Williams, D. , Sanna, P. P. , & Baldwin, K. K. (2015). Selective conversion of fibroblasts into peripheral sensory neurons. Nature Neuroscience, 18(1), 25. 10.1038/NN.3887 25420069 PMC4466122

[wsbm1570-bib-0026] Bölcskei, K. , Helyes, Z. , Szabó, Á. , Sándor, K. , Elekes, K. , Németh, J. , Almási, R. , Pintér, E. , Petho, G. , & Szolcsányi, J. (2005). Investigation of the role of TRPV1 receptors in acute and chronic nociceptive processes using gene‐deficient mice. Pain, 117(3), 368–376. 10.1016/J.PAIN.2005.06.024 16150543

[wsbm1570-bib-0027] Bonnet, C. , Hao, J. , Osorio, N. , Donnet, A. , Penalba, V. , Ruel, J. , & Delmas, P. (2019). Maladaptive activation of Nav1.9 channels by nitric oxide causes triptan‐induced medication overuse headache. Nature Communications, 10(1), 1–13. 10.1038/s41467-019-12197-3 PMC6751217 31534133

[wsbm1570-bib-0028] Brifault, C. , Romero, H. , Van‐Enoo, A. , Pizzo, D. , Azmoon, P. , Kwon, H. , Nasamran, C. , Gonias, S. L. , & Campana, W. M. (2020). Deletion of the gene encoding the NMDA receptor GluN1 subunit in Schwann cells causes ultrastructural changes in Remak bundles and hypersensitivity in pain processing. Journal of Neuroscience, 40(47), 9121–9136. 10.1523/JNEUROSCI.0663-20.2020 33051351 PMC7672997

[wsbm1570-bib-0029] Cao, Y. Q. , Mantyh, P. W. , Carlson, E. J. , Gillespie, A.‐M. , Epstein, C. J. , & Basbaum, A. I. (1998). Primary afferent tachykinins are required to experience moderate to intense pain. Nature, 392(6674), 390–394. 10.1038/32897 9537322

[wsbm1570-bib-0030] Caterina, M. J. , Leffler, A. , Malmberg, A. B. , Martin, W. J. , Trafton, J. , Petersen‐Zeitz, K. R. , Koltzenburg, M. , Basbaum, A. I. , & Julius, D. (2000). Impaired nociception and pain sensation in mice lacking the capsaicin receptor. Science, 288(5464), 306–313. 10.1126/SCIENCE.288.5464.306 10764638

[wsbm1570-bib-0031] Caterina, M. J. , Schumacher, M. A. , Tominaga, M. , Rosen, T. A. , Levine, J. D. , & Julius, D. (1997). The capsaicin receptor: A heat‐activated ion channel in the pain pathway. Nature, 389(6653), 816–824. 10.1038/39807 9349813

[wsbm1570-bib-0032] Cattaruzza, F. , Spreadbury, I. , Miranda‐Morales, M. , Grady, E. F. , Vanner, S. , & Bunnett, N. W. (2010). Transient receptor potential ankyrin‐1 has a major role in mediating visceral pain in mice. American Journal of Physiology. Gastrointestinal and Liver Physiology, 298(1), 81–91. 10.1152/Ajpgi.00221.2009 PMC2806099 19875705

[wsbm1570-bib-0033] Catterall, W. A. (1995). Structure and function of voltage‐gated ion channels. Annual Review of Biochemistry, 64, 493–531. 10.1146/ANNUREV.BI.64.070195.002425 7574491

[wsbm1570-bib-0034] Chambers, S. M. , Qi, Y. , Mica, Y. , Lee, G. , Zhang, X.‐J. , Niu, L. , Bilsland, J. , Cao, L. , Stevens, E. , Whiting, P. , Shi, S.‐H. , & Studer, L. (2012). Combined small‐molecule inhibition accelerates developmental timing and converts human pluripotent stem cells into nociceptors. Nature Biotechnology, 30(7), 715–720. 10.1038/nbt.2249 PMC3516136 22750882

[wsbm1570-bib-0035] Chen, C.‐L. , Broom, D. C. , Liu, Y. , de Nooij, J. C. , Li, Z. , Cen, C. , Samad, O. A. , Jessell, T. M. , Woolf, C. J. , & Ma, Q. (2006). Runx1 determines nociceptive sensory neuron phenotype and is required for thermal and neuropathic pain. Neuron, 49(3), 365–377. 10.1016/J.NEURON.2005.10.036 16446141

[wsbm1570-bib-0036] Chen, E. Y. , Tan, C. M. , Kou, Y. , Duan, Q. , Wang, Z. , Meirelles, G. V. , Clark, N. R. , & Ma'ayan, A. (2013). Enrichr: Interactive and collaborative HTML5 gene list enrichment analysis tool. BMC Bioinformatics, 14(1), 1–14. 10.1186/1471-2105-14-128/FIGURES/3 23586463 PMC3637064

[wsbm1570-bib-0037] Chen, G. , Zhang, Y.‐Q. , Qadri, Y. J. , Serhan, C. N. , & Ji, R.‐R. (2018). Microglia in pain: Detrimental and protective roles in pathogenesis and resolution of pain. Neuron, 100(6), 1292. 10.1016/J.NEURON.2018.11.009 30571942 PMC6312407

[wsbm1570-bib-0038] Chen, Y.‐C. , Auer‐Grumbach, M. , Matsukawa, S. , Zitzelsberger, M. , Themistocleous, A. C. , Strom, T. M. , Samara, C. , Moore, A. W. , Cho, L. T.‐Y. , Young, G. T. , Weiss, C. , Schabhüttl, M. , Stucka, R. , Schmid, A. B. , Parman, Y. , Graul‐Neumann, L. , Heinritz, W. , Passarge, E. , Watson, R. M. , … Senderek, J. (2015). Transcriptional regulator PRDM12 is essential for human pain perception. Nature Genetics, 47(7), 803–808. 10.1038/ng.3308 26005867 PMC7212047

[wsbm1570-bib-0039] Cheng, H. T. , Suzuki, M. , Hegarty, D. M. , Xu, Q. , Weyerbacher, A. R. , South, S. M. , Ohata, M. , & Inturrisi, C. E. (2008). Inflammatory pain‐induced signaling events following a conditional deletion of the N‐methyl‐d‐aspartate receptor in spinal cord dorsal horn. Neuroscience, 155(3), 948–958. 10.1016/J.NEUROSCIENCE.2008.06.024 18621103 PMC2556960

[wsbm1570-bib-0040] Chiechio, S. , & Nicoletti, F. (2012). Metabotropic glutamate receptors and the control of chronic pain. Current Opinion in Pharmacology, 12(1), 28–34. 10.1016/J.COPH.2011.10.010 22040745

[wsbm1570-bib-0041] Chiu, J. J. , Sgagias, M. K. , & Cowan, K. H. (1996). Interleukin 6 acts as a paracrine growth factor in human mammary carcinoma cell lines. Clinical Cancer Research, 2(1), 215–221. 9816109

[wsbm1570-bib-0042] Chong, J. A. , Tapia‐Ramirez, J. , Kim, S. , Toledo‐Aral, J. J. , Zheng, Y. , Boutros, M. C. , Altshuller, Y. M. , Frohman, M. A. , Kraner, S. D. , & Mandel, G. (1995). REST: A mammalian silencer protein that restricts Sodium Channel gene expression to neurons. Cell, 80, 949–957. 7697725 10.1016/0092-8674(95)90298-8

[wsbm1570-bib-0043] Cobos, E. J. , Nickerson, C. A. , Gao, F. , Chandran, V. , Bravo‐Caparrós, I. , González‐Cano, R. , Riva, P. , Andrews, N. A. , Latremoliere, A. , Seehus, C. R. , Perazzoli, G. , Nieto, F. R. , Joller, N. , Painter, M. W. , Ma, C. H. E. , Omura, T. , Chesler, E. J. , Geschwind, D. H. , Coppola, G. , … Costigan, M. (2018). Mechanistic differences in neuropathic pain modalities revealed by correlating behavior with global expression profiling. Cell Reports, 22(5), 1301. 10.1016/J.CELREP.2018.01.006 29386116 PMC5908229

[wsbm1570-bib-0044] Cohen, S. , & Levi‐Montalcini, R. (1956). A nerve growth‐stimulating factor isolated from snake venom. Proceedings of the National Academy of Sciences of the United States of America, 42(9), 571–574. 10.1073/PNAS.42.9.571 16589907 PMC534252

[wsbm1570-bib-0045] Colburn, R. W. , Lubin, M. L. , Stone, D. J. , Wang, Y. , Lawrence, D. , D'Andrea, M. R. R. , Brandt, M. R. , Liu, Y. , Flores, C. M. , & Qin, N. (2007). Attenuated cold sensitivity in TRPM8 null mice. Neuron, 54(3), 379–386. 10.1016/J.NEURON.2007.04.017 17481392

[wsbm1570-bib-0046] Colloca, L. , Ludman, T. , Bouhassira, D. , Baron, R. , Dickenson, A. H. , Yarnitsky, D. , Freeman, R. , Truini, A. , Attal, N. , Finnerup, N. B. , Eccleston, C. , Kalso, E. , Bennett, D. L. , Dworkin, R. H. , & Raja, S. N. (2017). Neuropathic pain. Nature Reviews Disease Primers, 3(1), 1–19. 10.1038/nrdp.2017.2 PMC5371025 28205574

[wsbm1570-bib-0047] Combs, C. K. , Karlo, J. C. , Kao, S.‐C. , & Landreth, G. E. (2001). β‐Amyloid stimulation of microglia and monocytes results in TNFα‐dependent expression of inducible nitric oxide synthase and neuronal apoptosis. Journal of Neuroscience, 21(4), 1179–1188. 10.1523/JNEUROSCI.21-04-01179.2001 11160388 PMC6762255

[wsbm1570-bib-0048] Corder, G. , Tawfik, V. L. , Wang, D. , Sypek, E. I. , Low, S. A. , Dickinson, J. R. , Sotoudeh, C. , Clark, J. D. , Barres, B. A. , Bohlen, C. J. , & Scherrer, G. (2017). Loss of μ opioid receptor signaling in nociceptors, but not microglia, abrogates morphine tolerance without disrupting analgesia. Nature Medicine, 23(2), 164–173. 10.1038/nm.4262 PMC5296291 28092666

[wsbm1570-bib-0049] Costigan, M. , Scholz, J. , & Woolf, C. J. (2009). Neuropathic pain: A maladaptive response of the nervous system to damage. Annual Review of Neuroscience, 32, 1–32. 10.1146/ANNUREV.NEURO.051508.135531 PMC2768555 19400724

[wsbm1570-bib-0050] Coulson, J. M. (2005). Transcriptional regulation: Cancer, neurons and the REST. Current Biology, 15(17), R665–R668. 10.1016/J.CUB.2005.08.032 16139198

[wsbm1570-bib-0051] Cox, J. J. , Reimann, F. , Nicholas, A. K. , Thornton, G. , Roberts, E. , Springell, K. , Karbani, G. , Jafri, H. , Mannan, J. , Raashid, Y. , Al‐Gazali, L. , Hamamy, H. , Valente, E. M. , Gorman, S. , Williams, R. , McHale, D. P. , Wood, J. N. , Gribble, F. M. , & Woods, C. G. (2006). An SCN9A channelopathy causes congenital inability to experience pain. Nature, 444(7121), 894–898. 10.1038/nature05413 17167479 PMC7212082

[wsbm1570-bib-0052] Cregg, R. , Momin, A. , Rugiero, F. , Wood, J. N. , & Zhao, J. (2010). Pain channelopathies. The Journal of Physiology, 588(Pt 11), 1897. 10.1113/JPHYSIOL.2010.187807 20142270 PMC2901978

[wsbm1570-bib-0053] Crowley, C. , Spencer, S. D. , Nishimura, M. C. , Chen, K. S. , Pitts‐Meek, S. , Armaninl, M. P. , Ling, L. H. , McMahon, S. B. , Shelton, D. L. , Levinson, A. D. , & Phillips, H. S. (1994). Mice lacking nerve growth factor display perinatal loss of sensory and sympathetic neurons yet develop basal forebrain cholinergic neurons. Cell, 76(6), 1001–1011. 10.1016/0092-8674(94)90378-6 8137419

[wsbm1570-bib-0054] Crupi, R. , Impellizzeri, D. , & Cuzzocrea, S. (2019). Role of metabotropic glutamate receptors in neurological disorders. Frontiers in Molecular Neuroscience, 12, 20. 10.3389/FNMOL.2019.00020 30800054 PMC6375857

[wsbm1570-bib-0055] Dahlhamer, J. (2019). Prevalence of chronic pain and high‐impact chronic pain among adults—United States, 2016. MMWR. Morbidity and Mortality Weekly Report, 67(36), 1001–1006. 10.15585/MMWR.MM6736A2 PMC6146950 30212442

[wsbm1570-bib-0056] de Felipe, C. , Herrero, J. F. , O'Brien, J. A. , Palmer, J. A. , Doyle, C. A. , Smith, A. J. H. , Laird, J. M. A. , Belmonte, C. , Cervero, F. , & Hunt, S. P. (1998). Altered nociception, analgesia and aggression in mice lacking the receptor for substance P. Nature, 392(6674), 394–397. 10.1038/32904 9537323

[wsbm1570-bib-0057] de la Peña, J. B. , Barragan‐Iglesias, P. , Lou, T.‐F. , Kunder, N. , Loerch, S. , Shukla, T. , Basavarajappa, L. , Song, J. , James, D. N. , Megat, S. , Moy, J. K. , Wanghzou, A. , Ray, P. R. , Hoyt, K. , Steward, O. , Price, T. J. , Shepherd, J. , & Campbell, Z. T. (2021). Intercellular arc signaling regulates vasodilation. Journal of Neuroscience, 41(37), 7712–7726. 10.1523/JNEUROSCI.0440-21.2021 34326146 PMC8445061

[wsbm1570-bib-0058] de la Peña, J. B. , & Campbell, Z. T. (2018). RNA‐binding proteins as targets for pain therapeutics. Neurobiology of Pain, 4, 2–7. 10.1016/J.YNPAI.2018.01.003 30370343 PMC6201239

[wsbm1570-bib-0059] de la Peña, J. B. , Kunder, N. , Lou, T.‐F. , Chase, R. , Stanowick, A. , Barragan‐Iglesias, P. , Pancrazio, J. J. , & Campbell, Z. T. (2021). A role for translational regulation by S6 kinase and a downstream target in inflammatory pain. British Journal of Pharmacology, 178(23), 4675–4690. 10.1111/BPH.15646 34355805 PMC9169231

[wsbm1570-bib-0060] de Lera Ruiz, M. , & Kraus, R. L. (2015). Voltage‐gated sodium channels: Structure, function, pharmacology, and clinical indications. Journal of Medicinal Chemistry, 58(18), 7093–7118. 10.1021/JM501981G/ASSET/IMAGES/LARGE/JM-2014-01981G_0013.JPEG 25927480

[wsbm1570-bib-0061] de Logu, F. , de Prá, S. D. T. , de David Antoniazzi, C. T. , Kudsi, S. Q. , Ferro, P. R. , Landini, L. , Rigo, F. K. , de Bem Silveira, G. , Silveira, P. C. L. , Oliveira, S. M. , Marini, M. , Mattei, G. , Ferreira, J. , Geppetti, P. , Nassini, R. , & Trevisan, G. (2020). Macrophages and Schwann cell TRPA1 mediate chronic allodynia in a mouse model of complex regional pain syndrome type I. Brain, Behavior, and Immunity, 88, 535–546. 10.1016/J.BBI.2020.04.037 32315759

[wsbm1570-bib-0062] de Oliveira, C. , Garami, A. , Lehto, S. G. , Pakai, E. , Tekus, V. , Pohoczky, K. , Youngblood, B. D. , Wang, W. , Kort, M. E. , Kym, P. R. , Pinter, E. , Gavva, N. R. , & Romanovsky, A. A. (2014). Transient receptor potential channel Ankyrin‐1 is not a cold sensor for autonomic thermoregulation in rodents. Journal of Neuroscience, 34(13), 4445–4452. 10.1523/JNEUROSCI.5387-13.2014 24671991 PMC3965775

[wsbm1570-bib-0063] de Sousa Valente, J. , Alawi, K. M. , Keringer, P. , Bharde, S. , Ayaz, F. , Saleque, N. , Kodji, X. , Thapa, D. , Argunhan, F. , & Brain, S. D. (2020). Examining the role of transient receptor potential canonical 5 (TRPC5) in osteoarthritis. Osteoarthritis and Cartilage Open, 2(4), 100119. 10.1016/J.OCARTO.2020.100119 33381767 PMC7762818

[wsbm1570-bib-0064] Dedek, A. , & Hildebrand, M. E. (2022). Advances and barriers in understanding presynaptic N‐methyl‐D‐aspartate receptors in spinal pain processing. Frontiers in Molecular Neuroscience, 127, 1–10. 10.3389/FNMOL.2022.864502 PMC9008455 35431805

[wsbm1570-bib-0065] Dembo, T. , Braz, J. M. , Hamel, K. A. , Kuhn, J. A. , & Basbaum, A. I. (2018). Primary afferent‐derived BDNF contributes minimally to the processing of pain and itch. ENeuro, 5(6), 1–14. 10.1523/ENEURO.0402-18.2018 PMC6325548 30627644

[wsbm1570-bib-0066] Deuis, J. R. , Wingerd, J. S. , Winter, Z. , Durek, T. , Dekan, Z. , Sousa, S. R. , Zimmermann, K. , Hoffmann, T. , Weidner, C. , Nassar, M. A. , Alewood, P. F. , Lewis, R. J. , & Vetter, I. (2016). Analgesic effects of GpTx‐1, PF‐04856264 and CNV1014802 in a mouse model of NaV1.7‐mediated pain. Toxins, 8(3), 78. 10.3390/TOXINS8030078 26999206 PMC4810223

[wsbm1570-bib-0067] Devesa, I. , Ferrándiz‐Huertas, C. , Mathivanan, S. , Wolf, C. , Luján, R. , Changeux, J.‐P. , & Ferrer‐Montiel, A. (2014). αCGRP is essential for algesic exocytotic mobilization of TRPV1 channels in peptidergic nociceptors. Proceedings of the National Academy of Sciences, 111(51), 18345–18350. 10.1073/PNAS.1420252111 PMC4280602 25489075

[wsbm1570-bib-0068] Dhaka, A. , Murray, A. N. , Mathur, J. , Earley, T. J. , Petrus, M. J. , & Patapoutian, A. (2007). TRPM8 is required for cold sensation in mice. Neuron, 54(3), 371–378. 10.1016/J.NEURON.2007.02.024 17481391

[wsbm1570-bib-0069] Diatchenko, L. , Nackley, A. G. , Tchivileva, I. E. , Shabalina, S. A. , & Maixner, W. (2007). Genetic architecture of human pain perception. Trends in Genetics, 23(12), 605–613. 10.1016/J.TIG.2007.09.004 18023497

[wsbm1570-bib-0070] Dib‐Hajj, S. D. , Rush, A. M. , Cummins, T. R. , Hisama, F. M. , Novella, S. , Tyrrell, L. , Marshall, L. , & Waxman, S. G. (2005). Gain‐of‐function mutation in Nav1.7 in familial erythromelalgia induces bursting of sensory neurons. Brain, 128(8), 1847–1854. 10.1093/brain/awh514 15958509

[wsbm1570-bib-0071] Donkin, J. J. , Turner, R. J. , Hassan, I. , & Vink, R. (2007). Substance P in traumatic brain injury. Progress in Brain Research, 161, 97–109. 10.1016/S0079-6123(06)61007-8 17618972

[wsbm1570-bib-0072] Dowal, L. , Provitera, P. , & Scarlata, S. (2006). Stable association between Gαq and phospholipase Cβ1 in living cells. Journal of Biological Chemistry, 281(33), 23999–24014. 10.1074/JBC.M512330200/ATTACHMENT/B638401F-B5B7-446B-8DD0-1DAA72FEB42C/MMC1.PDF 16754659

[wsbm1570-bib-0073] Durant, C. F. , Paterson, L. M. , Turton, S. , Wilson, S. J. , Myers, J. F. M. , Muthukumaraswamy, S. , Venkataraman, A. , Mick, I. , Paterson, S. , Jones, T. , Nahar, L. K. , Cordero, R. E. , Nutt, D. J. , & Lingford‐Hughes, A. (2018). Using baclofen to explore GABA‐B receptor function in alcohol dependence: Insights from pharmacokinetic and Pharmacodynamic measures. Frontiers in Psychiatry, 0, 664. 10.3389/FPSYT.2018.00664 PMC6302106 30618857

[wsbm1570-bib-0074] Durante, M. , Squillace, S. , Lauro, F. , Giancotti, L. A. , Coppi, E. , Cherchi, F. , Mannelli, L. D. C. , Ghelardini, C. , Kolar, G. , Wahlman, C. , Opejin, A. , Xiao, C. , Reitman, M. L. , Tosh, D. K. , Hawiger, D. , Jacobson, K. A. , & Salvemini, D. (2021). Adenosine A3 agonists reverse neuropathic pain via T cell–mediated production of IL‐10. The Journal of Clinical Investigation, 131(7), 1–6. 10.1172/JCI139299 PMC8011899 33621215

[wsbm1570-bib-0075] Dutra, R. C. , Bento, A. F. , Leite, D. F. P. , Manjavachi, M. N. , Marcon, R. , Bicca, M. A. , Pesquero, J. B. , & Calixto, J. B. (2013). The role of kinin B1 and B2 receptors in the persistent pain induced by experimental autoimmune encephalomyelitis (EAE) in mice: Evidence for the involvement of astrocytes. Neurobiology of Disease, 54, 82–93. 10.1016/J.NBD.2013.02.007 23454198

[wsbm1570-bib-0076] Edvinsson, L. (2019). Role of CGRP in migraine. Handbook of Experimental Pharmacology, 255, 121–130. 10.1007/164_2018_201 30725283

[wsbm1570-bib-0077] Enna, S. J. , & McCarson, K. E. (2006). The role of GABA in the mediation and perception of pain. Advances in Pharmacology, 54, 1–27. 10.1016/S1054-3589(06)54001-3 17175808

[wsbm1570-bib-0078] Faroni, A. , Castelnovo, L. F. , Procacci, P. , Caffino, L. , Fumagalli, F. , Melfi, S. , Gambarotta, G. , Bettler, B. , Wrabetz, L. , & Magnaghi, V. (2014). Deletion of GABA‐B receptor in Schwann cells regulates Remak bundles and small nociceptive C‐fibers. Glia, 62(4), 548–565. 10.1002/GLIA.22625 24474699

[wsbm1570-bib-0079] Ferrari, L. F. , Khomula, E. v. , Araldi, D. , & Levine, J. D. (2016). Marked sexual dimorphism in the role of the ryanodine receptor in a model of pain Chronification in the rat. Scientific Reports, 6(1), 1–12. 10.1038/srep31221 27499186 PMC4976309

[wsbm1570-bib-0080] Ferreira, J. , Beirith, A. , Mori, M. A. S. , Araújo, R. C. , Bader, M. , Pesquero, J. B. , & Calixto, J. B. (2005). Reduced nerve injury‐induced neuropathic pain in Kinin B1 receptor Knock‐out mice. Journal of Neuroscience, 25(9), 2405–2412. 10.1523/JNEUROSCI.2466-04.2005 15745967 PMC6726078

[wsbm1570-bib-0081] Ferreira, J. , Campos, M. M. , Pesquero, J. B. , Araújo, R. C. , Bader, M. , & Calixto, J. B. (2001). Evidence for the participation of kinins in Freund's adjuvant‐induced inflammatory and nociceptive responses in kinin B1 and B2 receptor knockout mice. Neuropharmacology, 41(8), 1006–1012. 10.1016/S0028-3908(01)00142-3 11747905

[wsbm1570-bib-0082] Ferreira, J. , Trichês, K. M. , Medeiros, R. , Cabrini, D. A. , Mori, M. A. S. , Pesquero, J. B. , Bader, M. , & Calixto, J. B. (2008). The role of kinin B1 receptors in the nociception produced by peripheral protein kinase C activation in mice. Neuropharmacology, 54(3), 597–604. 10.1016/J.NEUROPHARM.2007.11.008 18164734

[wsbm1570-bib-0083] Fischer, B. D. (2017). GABA A receptors as targets for the management of pain‐related disorders: Historical perspective and update. CNS & Neurological Disorders ‐ Drug Targets, 16(6), 658–663. 10.2174/1871527316666170207155149 28176641

[wsbm1570-bib-0084] Fishman, P. , Bar‐Yehuda, S. , Liang, B. T. , & Jacobson, K. A. (2012). Pharmacological and therapeutic effects of A3 adenosine receptor agonists. Drug Discovery Today, 17(7–8), 359–366. 10.1016/J.DRUDIS.2011.10.007 22033198 PMC3289754

[wsbm1570-bib-0085] Fitzcharles, M. A. , Cohen, S. P. , Clauw, D. J. , Littlejohn, G. , Usui, C. , & Häuser, W. (2021). Nociplastic pain: Towards an understanding of prevalent pain conditions. The Lancet, 397(10289), 2098–2110. 10.1016/S0140-6736(21)00392-5 34062144

[wsbm1570-bib-0086] Floris, M. , Olla, S. , Schlessinger, D. , & Cucca, F. (2018). Genetic‐driven druggable target identification and validation. Trends in Genetics, 34(7), 558. 10.1016/J.TIG.2018.04.004 29803319 PMC6088790

[wsbm1570-bib-0087] Flynn, R. , Chapman, K. , Iftinca, M. , Aboushousha, R. , Varela, D. , & Altier, C. (2014). Targeting the transient receptor potential Vanilloid type 1 (TRPV1) assembly domain attenuates inflammation‐induced hypersensitivity. Journal of Biological Chemistry, 289(24), 16675–16687. 10.1074/JBC.M114.558668 24808184 PMC4059113

[wsbm1570-bib-0088] Freidin, M. , & Kessler, J. A. (1991). Cytokine regulation of substance P expression in sympathetic neurons. Proceedings of the National Academy of Sciences of the United States of America, 88(8), 3200–3203. 10.1073/PNAS.88.8.3200 1707535 PMC51413

[wsbm1570-bib-0089] Fuchs, P. N. , Roza, C. , Sora, I. , Uhl, G. , & Raja, S. N. (1999). Characterization of mechanical withdrawal responses and effects of μ‐, δ‐ and κ‐opioid agonists in normal and μ‐opioid receptor knockout mice. Brain Research, 821(2), 480–486. 10.1016/S0006-8993(99)01060-4 10064835

[wsbm1570-bib-0090] Fundytus, M. E. , Yashpal, K. , Chabot, J.‐G. , Osborne, M. G. , Lefebvre, C. D. , Dray, A. , Henry, J. L. , & Coderre, T. J. (2001). Knockdown of spinal metabotropic glutamate receptor 1 (mGluR1) alleviates pain and restores opioid efficacy after nerve injury in rats. British Journal of Pharmacology, 132(1), 354–367. 10.1038/SJ.BJP.0703810 11156596 PMC1572554

[wsbm1570-bib-0091] Gangadharan, V. , & Kuner, R. (2013). Pain hypersensitivity mechanisms at a glance. Disease Models & Mechanisms, 6(4), 889. 10.1242/DMM.011502 23828645 PMC3701208

[wsbm1570-bib-0092] Gao, Y. J. , Xu, Z. Z. , Liu, Y. C. , Wen, Y. R. , Decosterd, I. , & Ji, R. R. (2010). The c‐Jun N‐terminal kinase 1 (JNK1) in spinal astrocytes is required for the maintenance of bilateral mechanical allodynia under a persistent inflammatory pain condition. Pain, 148(2), 309–319. 10.1016/J.PAIN.2009.11.017 20022176 PMC2814908

[wsbm1570-bib-0093] Gardell, L. R. , Ossipov, M. H. , Vanderah, T. W. , Lai, J. , & Porreca, F. (2002). Dynorphin‐independent spinal cannabinoid antinociception. Pain, 100(3), 243–248. 10.1016/S0304-3959(02)00173-2 12467995

[wsbm1570-bib-0094] Garza, A. , Weinstock, J. , & Robinson, P. (2008). Absence of the SP/SP receptor circuitry in the substance P‐precursor knockout mice or SP receptor, neurokinin (NK)1 knockout mice leads to an inhibited cytokine response in granulomas associated with murine Taenia crassiceps infection. The Journal of Parasitology, 94(6), 1253–1258. 10.1645/GE-1481.1 18576810 PMC2647574

[wsbm1570-bib-0095] Gaskin, D. J. , & Richard, P. (2012). The economic costs of pain in the United States. The Journal of Pain, 13(8), 715–724. 10.1016/J.JPAIN.2012.03.009 22607834

[wsbm1570-bib-0096] Gavva, N. R. , Treanor, J. J. S. , Garami, A. , Fang, L. , Surapaneni, S. , Akrami, A. , Alvarez, F. , Bak, A. , Darling, M. , Gore, A. , Jang, G. R. , Kesslak, J. P. , Ni, L. , Norman, M. H. , Palluconi, G. , Rose, M. J. , Salfi, M. , Tan, E. , Romanovsky, A. A. , … Davar, G. (2008). Pharmacological blockade of the vanilloid receptor TRPV1 elicits marked hyperthermia in humans. Pain, 136(1–2), 202–210. 10.1016/J.PAIN.2008.01.024 18337008

[wsbm1570-bib-0097] Gensel, J. C. , Donahue, R. R. , Bailey, W. M. , & Taylor, B. K. (2019). Sexual dimorphism of pain control: Analgesic effects of pioglitazone and azithromycin in chronic spinal cord injury. Journal of Neurotrauma, 36(15), 2372. 10.1089/NEU.2018.6207 30618345 PMC6648167

[wsbm1570-bib-0098] Gerber, G. , Zhong, J. , Youn, D. H. , & Randic, M. (2000). Group II and group III metabotropic glutamate receptor agonists depress synaptic transmission in the rat spinal cord dorsal horn. Neuroscience, 100(2), 393–406. 10.1016/S0306-4522(00)00269-4 11008177

[wsbm1570-bib-0099] Granan, L. P. (2017). We do not need a third mechanistic descriptor for chronic pain states! Not yet. Pain, 158(1), 179. 10.1097/J.PAIN.0000000000000735 27984528 PMC5175995

[wsbm1570-bib-0100] Guan, Z. , Kuhn, J. A. , Wang, X. , Colquitt, B. , Solorzano, C. , Vaman, S. , Guan, A. K. , Evans‐Reinsch, Z. , Braz, J. , Devor, M. , Abboud‐Werner, S. L. , Lanier, L. L. , Lomvardas, S. , & Basbaum, A. I. (2015). Injured sensory neuron‐derived CSF1 induces microglial proliferation and DAP12‐dependent pain. Nature Neuroscience, 19(1), 94–101. 10.1038/nn.4189 26642091 PMC4703328

[wsbm1570-bib-0101] Gui, W.‐S. , Wei, X. , Mai, C.‐L. , Murugan, M. , Wu, L.‐J. , Xin, W.‐J. , Zhou, L.‐J. , & Liu, X.‐G. (2016). Interleukin‐1β overproduction is a common cause for neuropathic pain, memory deficit, and depression following peripheral nerve injury in rodents. Molecular Pain, 12, 1744806916646784. 10.1177/1744806916646784 27175012 PMC4956151

[wsbm1570-bib-0102] Hackam, D. G. , & Redelmeier, D. A. (2006). Translation of research evidence from animals to humans. JAMA, 296(14), 1731–1732. 10.1001/JAMA.296.14.1731 17032985

[wsbm1570-bib-0103] Hai, T. , & Hartman, M. G. (2001). The molecular biology and nomenclature of the activating transcription factor/cAMP responsive element binding family of transcription factors: Activating transcription factor proteins and homeostasis. Gene, 273(1), 1–11. 10.1016/S0378-1119(01)00551-0 11483355

[wsbm1570-bib-0104] Hammond, S. M. , Bernstein, E. , Beach, D. , & Hannon, G. J. (2000). An RNA‐directed nuclease mediates post‐transcriptional gene silencing in drosophila cells. Nature, 404(6775), 293–296. 10.1038/35005107 10749213

[wsbm1570-bib-0105] Han, L. , Ma, C. , Liu, Q. , Weng, H.‐J. , Cui, Y. , Tang, Z. , Kim, Y. , Nie, H. , Qu, L. , Patel, K. N. , Li, Z. , McNeil, B. , He, S. , Guan, Y. , Xiao, B. , LaMotte, R. H. , & Dong, X. (2012). A subpopulation of nociceptors specifically linked to itch. Nature Neuroscience, 16(2), 174–182. 10.1038/nn.3289 23263443 PMC3557753

[wsbm1570-bib-0106] Hanack, C. , Moroni, M. , Lima, W. C. , Wende, H. , Kirchner, M. , Adelfinger, L. , Schrenk‐Siemens, K. , Tappe‐Theodor, A. , Wetzel, C. , Kuich, P. H. , Gassmann, M. , Roggenkamp, D. , Bettler, B. , Lewin, G. R. , Selbach, M. , & Siemens, J. (2015). GABA blocks pathological but not acute TRPV1 pain signals. Cell, 160(4), 759–770. 10.1016/J.CELL.2015.01.022 25679765

[wsbm1570-bib-0107] Hoffmann, T. , Sharon, O. , Wittmann, J. , Carr, R. W. , Vyshnevska, A. , de Col, R. , Nassar, M. A. , Reeh, P. W. , & Weidner, C. (2018). NaV1.7 and pain: Contribution of peripheral nerves. Pain, 159(3), 496–506. 10.1097/J.PAIN.0000000000001119 29194125

[wsbm1570-bib-0108] Honore, P. , Wade, C. L. , Zhong, C. , Harris, R. R. , Wu, C. , Ghayur, T. , Iwakura, Y. , Decker, M. W. , Faltynek, C. , Sullivan, J. , & Jarvis, M. F. (2006). Interleukin‐1αβ gene‐deficient mice show reduced nociceptive sensitivity in models of inflammatory and neuropathic pain but not post‐operative pain. Behavioural Brain Research, 167(2), 355–364. 10.1016/J.BBR.2005.09.024 16256210

[wsbm1570-bib-0109] Hu, H.‐J. , Alter, B. J. , Carrasquillo, Y. , Qiu, C.‐S. , & Gereau, R. W. (2007). Metabotropic glutamate receptor 5 modulates nociceptive plasticity via extracellular signal‐regulated kinase–Kv4.2 signaling in spinal cord dorsal horn neurons. Journal of Neuroscience, 27(48), 13181–13191. 10.1523/JNEUROSCI.0269-07.2007 18045912 PMC6673402

[wsbm1570-bib-0110] Hu, H.‐J. , Bhave, G. , & Gereau, R. W. (2002). Prostaglandin and protein kinase A‐dependent modulation of Vanilloid receptor function by metabotropic glutamate receptor 5: Potential mechanism for thermal hyperalgesia. Journal of Neuroscience, 22(17), 7444–7452. 10.1523/JNEUROSCI.22-17-07444.2002 12196566 PMC6757997

[wsbm1570-bib-0111] Hu, T. T. , Wang, R. R. , Tang, Y. Y. , Wu, Y. X. , Yu, J. , Hou, W. W. , Lou, G. D. , Zhou, Y. D. , Zhang, S. H. , & Chen, Z. (2018). TLR4 deficiency abrogated widespread tactile allodynia, but not widespread thermal hyperalgesia and trigeminal neuropathic pain after partial infraorbital nerve transection. Pain, 159(2), 273–283. 10.1097/J.PAIN.0000000000001100 29112008

[wsbm1570-bib-0112] Huang, E. J. , & Reichardt, L. F. (2001). Neurotrophins: Roles in neuronal development and function. Annual Review of Neuroscience, 24, 677. 10.1146/ANNUREV.NEURO.24.1.677 PMC2758233 11520916

[wsbm1570-bib-0113] Hunyady, Á. , Hajna, Z. , Gubányi, T. , Scheich, B. , Kemény, Á. , Gaszner, B. , Borbély, É. , & Helyes, Z. (2019). Hemokinin‐1 is an important mediator of pain in mouse models of neuropathic and inflammatory mechanisms. Brain Research Bulletin, 147, 165–173. 10.1016/J.BRAINRESBULL.2019.01.015 30664920

[wsbm1570-bib-0114] Hussey, M. J. , Clarke, G. D. , Ledent, C. , Hourani, S. M. O. , & Kitchen, I. (2007). Reduced response to the formalin test and lowered spinal NMDA glutamate receptor binding in adenosine A2A receptor knockout mice. Pain, 129(3), 287–294. 10.1016/J.PAIN.2006.10.014 17134834

[wsbm1570-bib-0115] Hylands‐White, N. , Duarte, R. V. , & Raphael, J. H. (2016). An overview of treatment approaches for chronic pain management. Rheumatology International, 37(1), 29–42. 10.1007/S00296-016-3481-8 27107994

[wsbm1570-bib-0116] Imai, S. , Ikegami, D. , Yamashita, A. , Shimizu, T. , Narita, M. , Niikura, K. , Furuya, M. , Kobayashi, Y. , Miyashita, K. , Okutsu, D. , Kato, A. , Nakamura, A. , Araki, A. , Omi, K. , Nakamura, M. , James Okano, H. , Okano, H. , Ando, T. , Takeshima, H. , … Narita, M. (2013). Epigenetic transcriptional activation of monocyte chemotactic protein 3 contributes to long‐lasting neuropathic pain. Brain, 136(3), 828–843. 10.1093/BRAIN/AWS330 23364351

[wsbm1570-bib-0117] Inoue, M. , Mishina, M. , & Ueda, H. (2000). Enhanced nociception by exogenous and endogenous substance P given into the spinal cord in mice lacking NR2A/ε1, an NMDA receptor subunit. British Journal of Pharmacology, 129(2), 239–241. 10.1038/SJ.BJP.0703056 10694228 PMC1571840

[wsbm1570-bib-0118] Inquimbert, P. , Moll, M. , Latremoliere, A. , Tong, C. K. , Whang, J. , Sheehan, G. F. , Smith, B. M. , Korb, E. , Athié, M. C. P. , Babaniyi, O. , Ghasemlou, N. , Yanagawa, Y. , Allis, C. D. , Hof, P. R. , & Scholz, J. (2018). NMDA receptor activation underlies the loss of spinal dorsal horn neurons and the transition to persistent pain after peripheral nerve injury. Cell Reports, 23(9), 2678–2689. 10.1016/J.CELREP.2018.04.107 29847798 PMC6276118

[wsbm1570-bib-0119] Ivanina, T. , Varon, D. , Peleg, S. , Rishal, I. , Porozov, Y. , Dessauer, C. W. , Keren‐Raifman, T. , & Dascal, N. (2004). Gαi1 and Gαi3 differentially interact with, and regulate, the G protein‐activated K+ channel. Journal of Biological Chemistry, 279(17), 17260–17268. 10.1074/JBC.M313425200 14963032

[wsbm1570-bib-0120] Jeong, S.‐W. , & Ikeda, S. R. (2000). Effect of G protein heterotrimer composition on coupling of neurotransmitter receptors to N‐type Ca2+ channel modulation in sympathetic neurons. Proceedings of the National Academy of Sciences of the United States of America, 97(2), 907–912. 10.1073/PNAS.97.2.907 10639178 PMC15429

[wsbm1570-bib-0121] Ji, R.‐R. , Baba, H. , Brenner, G. J. , & Woolf, C. J. (1999). Nociceptive‐specific activation of ERK in spinal neurons contributes to pain hypersensitivity. Nature Neuroscience, 2(12), 1114–1119. 10.1038/16040 10570489

[wsbm1570-bib-0122] Ji, R.‐R. , Befort, K. , Brenner, G. J. , & Woolf, C. J. (2002). ERK MAP kinase activation in superficial spinal cord neurons induces Prodynorphin and NK‐1 upregulation and contributes to persistent inflammatory pain hypersensitivity. Journal of Neuroscience, 22(2), 478–485. 10.1523/JNEUROSCI.22-02-00478.2002 11784793 PMC6758654

[wsbm1570-bib-0123] Jones, K. D. , Gelbart, T. , Whisenant, T. C. , Waalen, J. , Mondala, T. S. , Iklé, D. N. , Salomon, D. R. , Bennett, R. M. , & Kurian, S. M. (2016). Genome‐wide expression profiling in the peripheral blood of patients with fibromyalgia. Clinical and Experimental Rheumatology, 34(2 Suppl 96), 89. PMC4888802 27157394

[wsbm1570-bib-0124] Jones, T. H. (1994). Interleukin‐6 an endocrine cytokine. Clinical Endocrinology, 40(6), 703–713. 10.1111/J.1365-2265.1994.TB02502.X 8033360

[wsbm1570-bib-0125] Julius, D. (2013). TRP channels and pain. Annual Review of Cell and Developmental Biology, 29, 355–384. 10.1146/ANNUREV-CELLBIO-101011-155833 24099085

[wsbm1570-bib-0126] Kamato, D. , Thach, L. , Bernard, R. , Chan, V. , Zheng, W. , Kaur, H. , Brimble, M. , Osman, N. , & Little, P. J. (2015). Structure, function, pharmacology, and therapeutic potential of the G protein, Gα/q,11. Frontiers in Cardiovascular Medicine, 2, 14. 10.3389/FCVM.2015.00014/BIBTEX 26664886 PMC4671355

[wsbm1570-bib-0127] Karim, F. , Hu, H.‐J. , Adwanikar, H. , Kaplan, D. , & Gereau, R. W. (2006). Impaired inflammatory pain and thermal hyperalgesia in mice expressing neuron‐specific dominant negative mitogen activated protein kinase kinase (MEK). Molecular Pain, 2, 2. 10.1186/1744-8069-2-2 16412244 PMC1382249

[wsbm1570-bib-0128] Karim, F. , Wang, C. C. , & Gereau, R. W., 4th . (2001). Metabotropic glutamate receptor subtypes 1 and 5 are activators of extracellular signal‐regulated kinase signaling required for inflammatory pain in mice. The Journal of Neuroscience, 21(11), 3771–3779. 10.1523/JNEUROSCI.21-11-03771.2001 11356865 PMC6762705

[wsbm1570-bib-0129] Khoutorsky, A. , Bonin, R. P. , Sorge, R. E. , Gkogkas, C. G. , Pawlowski, S. A. , Jafarnejad, S. M. , Pitcher, M. H. , Alain, T. , Perez‐Sanchez, J. , Salter, E. W. , Martin, L. , Ribeiro‐Da‐Silva, A. , de Koninck, Y. , Cervero, F. , Mogil, J. S. , & Sonenberg, N. (2015). Translational control of nociception via 4E‐binding protein 1. eLife, 4(DECEMBER2015), 1–18. 10.7554/ELIFE.12002 PMC4695384 26678009

[wsbm1570-bib-0130] Kingery, W. S. , Guo, T. Z. , Davies, M. F. , Limbird, L. , & Maze, M. (2000). The α(2A) adrenoceptor and the sympathetic postganglionic neuron contribute to the development of neuropathic heat hyperalgesia in mice. Pain, 85(3), 345–358. 10.1016/S0304-3959(99)00286-9 10781908

[wsbm1570-bib-0131] Kingwell, K. (2019). Nav1.7 withholds its pain potential. Nature Reviews. Drug Discovery, 321–323. 10.1038/D41573-019-00065-0 31048807

[wsbm1570-bib-0132] Knowlton, W. M. , Bifolck‐Fisher, A. , Bautista, D. M. , & McKemy, D. D. (2010). TRPM8, but not TRPA1, is required for neural and behavioral responses to acute noxious cold temperatures and cold‐mimetics in vivo. Pain, 150(2), 340–350. 10.1016/J.PAIN.2010.05.021 20542379 PMC2897947

[wsbm1570-bib-0133] Koivisto, A.‐P. , Belvisi, M. G. , Gaudet, R. , & Szallasi, A. (2021). Advances in TRP channel drug discovery: From target validation to clinical studies. Nature Reviews Drug Discovery, 2021, 1–19. 10.1038/s41573-021-00268-4 PMC8442523 34526696

[wsbm1570-bib-0134] Kokotović, T. , Langeslag, M. , Lenartowicz, E. M. , Manion, J. , Fell, C. W. , Alehabib, E. , Tafakhori, A. , Darvish, H. , Bellefroid, E. J. , Neely, G. G. , Kress, M. , Penninger, J. M. , & Nagy, V. (2021). PRDM12 is transcriptionally active and required for nociceptor function throughout life. Frontiers in Molecular Neuroscience, 192, 1–21. 10.3389/FNMOL.2021.720973 PMC8502974 34646120

[wsbm1570-bib-0135] Kolber, B. J. , Montana, M. C. , Carrasquillo, Y. , Xu, J. , Heinemann, S. F. , Muglia, L. J. , & Gereau, R. W. (2010). Activation of metabotropic glutamate receptor 5 in the amygdala modulates pain‐like behavior. Journal of Neuroscience, 30(24), 8203–8213. 10.1523/JNEUROSCI.1216-10.2010 20554871 PMC2898903

[wsbm1570-bib-0136] König, M. , Zimmer, A. M. , Steiner, H. , Holmes, P. v. , Crawley, J. N. , Brownstein, M. J. , & Zimmer, A. (1996). Pain responses, anxiety and aggression in mice deficient in pre‐proenkephalin. Nature, 383(6600), 535–538. 10.1038/383535a0 8849726

[wsbm1570-bib-0137] Kosek, E. , Cohen, M. , Baron, R. , Gebhart, G. F. , Mico, J. A. , Rice, A. S. C. , Rief, W. , & Sluka, A. K. (2016). Do we need a third mechanistic descriptor for chronic pain states? Pain, 157(7), 1382–1386. 10.1097/J.PAIN.0000000000000507 26835783

[wsbm1570-bib-0138] Kuleshov, M. V. , Jones, M. R. , Rouillard, A. D. , Fernandez, N. F. , Duan, Q. , Wang, Z. , Koplev, S. , Jenkins, S. L. , Jagodnik, K. M. , Lachmann, A. , McDermott, M. G. , Monteiro, C. D. , Gundersen, G. W. , & Ma'ayan, A. (2016). Enrichr: A comprehensive gene set enrichment analysis web server 2016 update. Nucleic Acids Research, 44(W1), W90–W97. 10.1093/NAR/GKW377 27141961 PMC4987924

[wsbm1570-bib-0139] Kupari, J. , Usoskin, D. , Parisien, M. , Lou, D. , Hu, Y. , Fatt, M. , Lönnerberg, P. , Spångberg, M. , Eriksson, B. , Barkas, N. , Kharchenko, P. v. , Loré, K. , Khoury, S. , Diatchenko, L. , & Ernfors, P. (2021). Single cell transcriptomics of primate sensory neurons identifies cell types associated with chronic pain. Nature Communications, 12(1), 1–15. 10.1038/s41467-021-21725-z PMC7940623 33686078

[wsbm1570-bib-0140] Kwan, K. Y. , Allchorne, A. J. , Vollrath, M. A. , Christensen, A. P. , Zhang, D. S. , Woolf, C. J. , & Corey, D. P. (2006). TRPA1 contributes to cold, mechanical, and chemical nociception but is not essential for hair‐cell transduction. Neuron, 50(2), 277–289. 10.1016/J.NEURON.2006.03.042 16630838

[wsbm1570-bib-0141] LaCroix‐Fralish, M. L. , Ledoux, J. B. , & Mogil, J. S. (2007). The pain genes database: An interactive web browser of pain‐related transgenic knockout studies. Pain, 131(1–2), 3.e1–3.e4. 10.1016/J.PAIN.2007.04.041 17574758

[wsbm1570-bib-0142] Laird, J. M. A. , Olivar, T. , Roza, C. , de Felipe, C. , Hunt, S. P. , & Cervero, F. (2000). Deficits in visceral pain and hyperalgesia of mice with a disruption of the tachykinin NK1 receptor gene. Neuroscience, 98(2), 345–352. 10.1016/S0306-4522(00)00148-2 10854767

[wsbm1570-bib-0143] Laird, J. M. A. , Souslova, V. , Wood, J. N. , & Cervero, F. (2002). Deficits in visceral pain and referred hyperalgesia in Nav1.8 (SNS/PN3)‐null mice. Journal of Neuroscience, 22(19), 8352–8356. 10.1523/JNEUROSCI.22-19-08352.2002 12351708 PMC6757795

[wsbm1570-bib-0144] Landy, M. A. , Goyal, M. , Casey, K. M. , Liu, C. , & Lai, H. C. (2021). Loss of Prdm12 during development, but not in mature nociceptors, causes defects in pain sensation. Cell Reports, 34(13), 108913. 10.1016/J.CELREP.2021.108913 33789102 PMC8048104

[wsbm1570-bib-0145] Latremoliere, A. , & Woolf, C. J. (2009). Central sensitization: A generator of pain hypersensitivity by central neural plasticity. The Journal of Pain, 10(9), 895. 10.1016/J.JPAIN.2009.06.012 19712899 PMC2750819

[wsbm1570-bib-0146] le Merrer, J. , Becker, J. A. J. , Befort, K. , & Kieffer, B. L. (2009). Reward processing by the opioid system in the Brain. Physiological Reviews, 89(4), 1379. 10.1152/PHYSREV.00005.2009 19789384 PMC4482114

[wsbm1570-bib-0147] Leaney, J. L. , Milligan, G. , & Tinker, A. (2000). The G protein α subunit has a key role in determining the specificity of coupling to, but not the activation of, G protein‐gated inwardly rectifying K+ channels. Journal of Biological Chemistry, 275(2), 921–929. 10.1074/JBC.275.2.921 10625628

[wsbm1570-bib-0148] Ledent, C. , Vaugeois, J.‐M. , Schiffmann, S. N. , Pedrazzini, T. , el Yacoubi, M. , Vanderhaeghen, J.‐J. , Costentin, J. , Heath, J. K. , Vassart, G. , & Parmentier, M. (1997). Aggressiveness, hypoalgesia and high blood pressure in mice lacking the adenosine A2a receptor. Nature, 388(6643), 674–678. 10.1038/41771 9262401

[wsbm1570-bib-0149] Lee, M. O. , Lee, M. , Silverman, S. , Hansen, H. , Patel, V. , & Manchikanti, L. (2011). A comprehensive review of opioid‐induced hyperalgesia. Pain Physician, 14, 145–161. 21412369

[wsbm1570-bib-0150] Lemoine, D. , Jiang, R. , Taly, A. , Chataigneau, T. , Specht, A. , & Grutter, T. (2012). Ligand‐gated ion channels: New insights into neurological disorders and ligand recognition. Chemical Reviews, 112(12), 6285–6318. 10.1021/CR3000829/ASSET/IMAGES/CR3000829.SOCIAL.JPEG_V03 22988962

[wsbm1570-bib-0151] Leppä, E. , Linden, A.‐M. , Vekovischeva, O. Y. , Swinny, J. D. , Rantanen, V. , Toppila, E. , Höger, H. , Sieghart, W. , Wulff, P. , Wisden, W. , & Korpi, E. R. (2011). Removal of GABAA receptor γ2 subunits from Parvalbumin neurons causes wide‐ranging behavioral alterations. PLoS One, 6(9), e24159. 10.1371/JOURNAL.PONE.0024159 21912668 PMC3166293

[wsbm1570-bib-0152] Li, S. , Wong, A. H. C. , & Liu, F. (2014). Ligand‐gated ion channel interacting proteins and their role in neuroprotection. Frontiers in Cellular Neuroscience, 8(MAY), 125. 10.3389/FNCEL.2014.00125/BIBTEX 24847210 PMC4023026

[wsbm1570-bib-0153] Li, W. , & Neugebauer, V. (2004). Differential roles of mGluR1 and mGluR5 in brief and prolonged nociceptive processing in central amygdala neurons. Journal of Neurophysiology, 91(1), 13–24. 10.1152/JN.00485.2003 13679408

[wsbm1570-bib-0154] Liao, H.‐Y. , Hsieh, C.‐L. , Huang, C.‐P. , & Lin, Y.‐W. (2017). Electroacupuncture attenuates CFA‐induced inflammatory pain by suppressing Nav1.8 through S100B, TRPV1, opioid, and adenosine pathways in mice. Scientific Reports, 7(1), 1–13. 10.1038/srep42531 28211895 PMC5304170

[wsbm1570-bib-0155] Lin, C.‐C. J. , Chen, W.‐N. , Chen, C.‐J. , Lin, Y.‐W. , Zimmer, A. , & Chen, C.‐C. (2012). An antinociceptive role for substance P in acid‐induced chronic muscle pain. Proceedings of the National Academy of Sciences of the United States of America, 109(2), E76–E83. 10.1073/PNAS.1108903108 22084095 PMC3258641

[wsbm1570-bib-0156] Little, J. W. , Ford, A. , Symons‐Liguori, A. M. , Chen, Z. , Janes, K. , Doyle, T. , Xie, J. , Luongo, L. , Tosh, D. K. , Maione, S. , Bannister, K. , Dickenson, A. H. , Vanderah, T. W. , Porreca, F. , Jacobson, K. A. , & Salvemini, D. (2015). Endogenous adenosine A3 receptor activation selectively alleviates persistent pain states. Brain, 138(1), 28. 10.1093/BRAIN/AWU330 25414036 PMC4285194

[wsbm1570-bib-0157] Liu, Y. , Li, Y. , Liu, Q. , Wu, Z. , Cui, J. , Zhu, K. , Zhao, H. , Zhou, C. , & Shi, B. (2019). Role of GM‐CSF in a mouse model of experimental autoimmune prostatitis. American Journal of Physiology. Renal Physiology, 317, 23–29. 10.1152/ajprenal.00013.2018.-The 30943070

[wsbm1570-bib-0158] Lo, U. , Selvaraj, V. , Plane, J. M. , Chechneva, O. v. , Otsu, K. , & Deng, W. (2014). p38α (MAPK14) critically regulates the immunological response and the production of specific cytokines and chemokines in astrocytes. Scientific Reports, 4(1), 1–18. 10.1038/srep07405 PMC4264013 25502009

[wsbm1570-bib-0159] Lolignier, S. , Amsalem, M. , Maingret, F. , Padilla, F. , Gabriac, M. , Chapuy, E. , Eschalier, A. , Delmas, P. , & Busserolles, J. (2011). Nav1.9 channel contributes to mechanical and heat pain hypersensitivity induced by subacute and chronic inflammation. PLoS One, 6(8), e23083. 10.1371/JOURNAL.PONE.0023083 21857998 PMC3155549

[wsbm1570-bib-0160] Lolignier, S. , Bonnet, C. , Gaudioso, C. , Noël, J. , Ruel, J. , Amsalem, M. , Ferrier, J. , Rodat‐Despoix, L. , Bouvier, V. , Aissouni, Y. , Prival, L. , Chapuy, E. , Padilla, F. , Eschalier, A. , Delmas, P. , & Busserolles, J. (2015). The Nav1.9 channel is a key determinant of cold pain sensation and cold allodynia. Cell Reports, 11(7), 1067–1078. 10.1016/J.CELREP.2015.04.027 25959819

[wsbm1570-bib-0161] Loyd, D. R. , & Murphy, A. Z. (2014). The neuroanatomy of sexual dimorphism in opioid analgesia. Experimental Neurology, 0, 57. 10.1016/J.EXPNEUROL.2014.04.004 PMC4127352 24731947

[wsbm1570-bib-0162] Luo, X. , Chen, O. , Wang, Z. , Bang, S. , Ji, J. , Lee, S. H. , Huh, Y. , Furutani, K. , He, Q. , Tao, X. , Ko, M.‐C. , Bortsov, A. , Donnelly, C. R. , Chen, Y. , Nackley, A. , Berta, T. , & Ji, R.‐R. (2021). IL‐23/IL‐17A/TRPV1 axis produces mechanical pain via macrophage‐sensory neuron crosstalk in female mice. Neuron, 109(17), 2691–2706.e5. 10.1016/J.NEURON.2021.06.015 34473953 PMC8425601

[wsbm1570-bib-0163] Luo, Y. , Kusay, A. S. , Jiang, T. , Chebib, M. , & Balle, T. (2021). Delta‐containing GABAA receptors in pain management: Promising targets for novel analgesics. Neuropharmacology, 195, 108675. 10.1016/J.NEUROPHARM.2021.108675 34153311

[wsbm1570-bib-0164] Mailhot, B. , Christin, M. , Tessandier, N. , Sotoudeh, C. , Bretheau, F. , Turmel, R. , Pellerin, È. , Wang, F. , Bories, C. , Joly‐Beauparlant, C. , de Koninck, Y. , Droit, A. , Cicchetti, F. , Scherrer, G. , Boilard, E. , Sharif‐Naeini, R. , & Lacroix, S. (2020). Neuronal interleukin‐1 receptors mediate pain in chronic inflammatory diseases. Journal of Experimental Medicine, 217(9), 1–18. 10.1084/JEM.20191430 32573694 PMC7478735

[wsbm1570-bib-0165] Maione, S. , Oliva, P. , Marabese, I. , Palazzo, E. , Rossi, F. , Berrino, L. , Rossi, F. , & Filippelli, A. (2000). Periaqueductal gray matter metabotropic glutamate receptors modulate formalin‐induced nociception. Pain, 85(1–2), 183–189. 10.1016/S0304-3959(99)00269-9 10692617

[wsbm1570-bib-0166] Malcangio, M. (2018). GABAB receptors and pain. Neuropharmacology, 136, 102–105. 10.1016/J.NEUROPHARM.2017.05.012 28504122

[wsbm1570-bib-0167] Manassero, G. , Repetto, I. E. , Cobianchi, S. , Valsecchi, V. , Bonny, C. , Rossi, F. , & Vercelli, A. (2012). Role of JNK isoforms in the development of neuropathic pain following sciatic nerve transection in the mouse. Molecular Pain, 8, 39. 10.1186/1744-8069-8-39 22616849 PMC3436729

[wsbm1570-bib-0168] Mansikka, H. , Lähdesmäki, J. , Scheinin, M. , & Pertovaara, A. (2004). α2AAdrenoceptors contribute to feedback inhibition of capsaicin‐induced hyperalgesia. Anesthesiology, 101(1), 185–190. 10.1097/00000542-200407000-00029 15220790

[wsbm1570-bib-0169] Mansikka, H. , Sheth, R. N. , DeVries, C. , Lee, H. , Winchurch, R. , & Raja, S. N. (2000). Nerve injury‐induced mechanical but not thermal hyperalgesia is attenuated in Neurokinin‐1 receptor knockout mice. Experimental Neurology, 162(2), 343–349. 10.1006/EXNR.1999.7336 10739640

[wsbm1570-bib-0170] Marabese, I. , de Novellis, V. , Palazzo, E. , Scafuro, M. A. , Vita, D. , Rossi, F. , & Maione, S. (2007). Effects of (S)‐3,4‐DCPG, an mGlu8 receptor agonist, on inflammatory and neuropathic pain in mice. Neuropharmacology, 52(2), 253–262. 10.1016/J.NEUROPHARM.2006.04.006 17113112

[wsbm1570-bib-0171] Marone, I. M. , de Logu, F. , Nassini, R. , de Carvalho Goncalves, M. , Benemei, S. , Ferreira, J. , Jain, P. , Li Puma, S. , Bunnett, N. W. , Geppetti, P. , & Materazzi, S. (2018). TRPA1/NOX in the soma of trigeminal ganglion neurons mediates migraine‐related pain of glyceryl trinitrate in mice. Brain, 141(8), 2312–2328. 10.1093/BRAIN/AWY177 29985973 PMC6061846

[wsbm1570-bib-0172] Martin, M. , Matifas, A. , Maldonado, R. , & Kieffer, B. L. (2003). Acute antinociceptive responses in single and combinatorial opioid receptor knockout mice: Distinct mu, delta and kappa tones. European Journal of Neuroscience, 17(4), 701–708. 10.1046/J.1460-9568.2003.02482.X 12603260

[wsbm1570-bib-0173] Marvaldi, L. , Panayotis, N. , Alber, S. , Daga, S. Y. , Okladnikov, N. , Koppel, I. , di Pizio, A. , Song, D. A. , Tzur, Y. , Terenzio, M. , Rishal, I. , Gordon, D. , Rother, F. , Hartmann, E. , Bader, M. , & Fainzilber, M. (2020). Importin a3 regulates chronic pain pathways in peripheral sensory neurons. Science, 369(6505), 842–846. 10.1126/SCIENCE.AAZ5875 32792398

[wsbm1570-bib-0174] Matthes, H. W. D. , Maldonado, R. , Simonin, F. , Valverde, O. , Slowe, S. , Kitchen, I. , Befort, K. , Dierich, A. , le Meur, M. , Dollé, P. , Tzavara, E. , Hanoune, J. , Roques, B. P. , & Kieffer, B. L. (1996). Loss of morphine‐induced analgesia, reward effect and withdrawal symptoms in mice lacking the μ‐opioid‐receptor gene. Nature, 383(6603), 819–823. 10.1038/383819a0 8893006

[wsbm1570-bib-0175] McCormack, K. , Santos, S. , Chapman, M. L. , Krafte, D. S. , Marron, B. E. , West, C. W. , Krambis, M. J. , Antonio, B. M. , Zellmer, S. G. , Printzenhoff, D. , Padilla, K. M. , Lin, Z. , Wagoner, P. K. , Swain, N. A. , Stupple, P. A. , de Groot, M. , Butt, R. P. , & Castle, N. A. (2013). Voltage sensor interaction site for selective small molecule inhibitors of voltage‐gated sodium channels. Proceedings of the National Academy of Sciences, 110(29), E2724–E2732. 10.1073/PNAS.1220844110 PMC3718154 23818614

[wsbm1570-bib-0176] McCoy, E. S. , Taylor‐Blake, B. , Street, S. E. , Pribisko, A. L. , Zheng, J. , & Zylka, M. J. (2013). Peptidergic CGRPα primary sensory neurons encode heat and itch and tonically suppress sensitivity to cold. Neuron, 78(1), 138–151. 10.1016/J.NEURON.2013.01.030 23523592 PMC3628403

[wsbm1570-bib-0177] McLaughlin, J. P. , Marton‐Popovici, M. , & Chavkin, C. (2003). κ opioid receptor antagonism and Prodynorphin gene disruption block stress‐induced behavioral responses. Journal of Neuroscience, 23(13), 5674–5683. 10.1523/JNEUROSCI.23-13-05674.2003 12843270 PMC2104777

[wsbm1570-bib-0178] Megat, S. , Ray, P. R. , Moy, J. K. , Lou, T.‐F. , Barragán‐Iglesias, P. , Li, Y. , Pradhan, G. , Wanghzou, A. , Ahmad, A. , Burton, M. D. , North, R. Y. , Dougherty, P. M. , Khoutorsky, A. , Sonenberg, N. , Webster, K. R. , Dussor, G. , Campbell, Z. T. , & Price, T. J. (2019). Nociceptor translational profiling reveals the Ragulator‐rag GTPase complex as a critical generator of neuropathic pain. Journal of Neuroscience, 39(3), 393–411. 10.1523/JNEUROSCI.2661-18.2018 30459229 PMC6335757

[wsbm1570-bib-0179] Megat, S. , Ray, P. R. , Tavares‐Ferreira, D. , Moy, J. K. , Sankaranarayanan, I. , Wangzhou, A. , Lou, T. F. , Barragan‐Iglesias, P. , Campbell, Z. T. , Dussor, G. , & Price, T. J. (2019). Differences between dorsal root and trigeminal ganglion nociceptors in mice revealed by translational profiling. Journal of Neuroscience, 39(35), 6829–6847. 10.1523/JNEUROSCI.2663-18.2019 31253755 PMC6733558

[wsbm1570-bib-0180] Melemedjian, O. K. , Asiedu, M. N. , Tillu, D. V. , Peebles, K. A. , Yan, J. , Ertz, N. , Dussor, G. O. , & Price, T. J. (2010). IL‐6‐ and NGF‐induced rapid control of protein synthesis and nociceptive plasticity via convergent signaling to the eIF4F complex. Journal of Neuroscience, 30(45), 15113–15123. 10.1523/JNEUROSCI.3947-10.2010 21068317 PMC3056511

[wsbm1570-bib-0181] Meloto, C. B. , Benavides, R. , Lichtenwalter, R. N. , Wen, X. , Tugarinov, N. , Zorina‐Lichtenwalter, K. , Chabot‐Doré, A. J. , Piltonen, M. H. , Cattaneo, S. , Verma, V. , Klares, R. , Khoury, S. , Parisien, M. , & Diatchenko, L. (2018). Human pain genetics database: A resource dedicated to human pain genetics research. Pain, 159(4), 749–763. 10.1097/J.PAIN.0000000000001135 29300278

[wsbm1570-bib-0182] Millan, M. J. (2002). Descending control of pain. Progress in Neurobiology, 66(6), 355–474. 10.1016/S0301-0082(02)00009-6 12034378

[wsbm1570-bib-0183] Minett, M. S. , Pereira, V. , Sikandar, S. , Matsuyama, A. , Lolignier, S. , Kanellopoulos, A. H. , Mancini, F. , Iannetti, G. D. , Bogdanov, Y. D. , Santana‐Varela, S. , Millet, Q. , Baskozos, G. , MacAllister, R. , Cox, J. J. , Zhao, J. , & Wood, J. N. (2015). Endogenous opioids contribute to insensitivity to pain in humans and mice lacking sodium channel Nav1.7. Nature Communications, 6(1), 1–8. 10.1038/ncomms9967 PMC4686868 26634308

[wsbm1570-bib-0184] Moalem, G. , & Tracey, D. J. (2006). Immune and inflammatory mechanisms in neuropathic pain. Brain Research Reviews, 51(2), 240–264. 10.1016/J.BRAINRESREV.2005.11.004 16388853

[wsbm1570-bib-0185] Moy, J. K. , Khoutorsky, A. , Asiedu, M. N. , Black, B. J. , Kuhn, J. L. , Barragán‐Iglesias, P. , Megat, S. , Burton, M. D. , Burgos‐Vega, C. C. , Melemedjian, O. K. , Boitano, S. , Vagner, J. , Gkogkas, C. G. , Pancrazio, J. J. , Mogil, J. S. , Dussor, G. , Sonenberg, N. , & Price, T. J. (2017). The MNK–eIF4E signaling Axis contributes to injury‐induced nociceptive plasticity and the development of chronic pain. Journal of Neuroscience, 37(31), 7481–7499. 10.1523/JNEUROSCI.0220-17.2017 28674170 PMC5546114

[wsbm1570-bib-0186] Muddhrry, P. K. , Ghatki, M. A. , Spokks, R. A. , Jonhs, P. M. , Pierson, A. M. , Hamid, Q. A. , Kanse, S. , Amara, S. G. , Burrik, J. M. , Legon, S. , Polak, J. M. , & Bloom, S. R. (1988). Differential expression of α‐CGRP and β‐CGRP by primary sensory neurons and enteric autonomic neurons of the rat. Neuroscience, 25(1), 195–205. 10.1016/0306-4522(88)90018-8 2839796

[wsbm1570-bib-0187] Murakami, M. , Fleischmann, B. , de Felipe, C. , Freichel, M. , Trost, C. , Ludwig, A. , Wissenbach, U. , Schwegler, H. , Hofmann, F. , Hescheler, J. , Flockerzi, V. , & Cavalié, A. (2002). Pain perception in mice lacking the β3 subunit of voltage‐activated calcium channels. Journal of Biological Chemistry, 277(43), 40342–40351. 10.1074/JBC.M203425200 12161429

[wsbm1570-bib-0188] Muroi, Y. , Ru, F. , Kollarik, M. , Canning, B. J. , Hughes, S. A. , Walsh, S. , Sigg, M. , Carr, M. J. , & Undem, B. J. (2011). Selective silencing of NaV1.7 decreases excitability and conduction in vagal sensory neurons. The Journal of Physiology, 589(23), 5663–5676. 10.1113/JPHYSIOL.2011.215384 22005676 PMC3249041

[wsbm1570-bib-0189] Murphy, P. G. , Ramer, M. S. , Borthwick, L. , Gauldie, J. , Richardson, P. M. , & Bisby, M. A. (1999). Endogenous interleukin‐6 contributes to hypersensitivity to cutaneous stimuli and changes in neuropeptides associated with chronic nerve constriction in mice. European Journal of Neuroscience, 11(7), 2243–2253. 10.1046/J.1460-9568.1999.00641.X 10383613

[wsbm1570-bib-0190] Nagy, V. , Cole, T. , van Campenhout, C. , Khoung, T. M. , Leung, C. , Vermeiren, S. , Novatchkova, M. , Wenzel, D. , Cikes, D. , Polyansky, A. A. , Kozieradzki, I. , Meixner, A. , Bellefroid, E. J. , Neely, G. G. , & Penninger, J. M. (2015). The evolutionarily conserved transcription factor PRDM12 controls sensory neuron development and pain perception. Cell Cycle, 14(12), 1799–1808. 10.1080/15384101.2015.1036209 25891934 PMC4613559

[wsbm1570-bib-0191] Nassar, M. A. , Stirling, L. C. , Forlani, G. , Baker, M. D. , Matthews, E. A. , Dickenson, A. H. , & Wood, J. N. (2004). Nociceptor‐specific gene deletion reveals a major role for Na. Proceedings of the National Academy of Sciences of the United States of America, 101(34), 12706–12711. 15314237 10.1073/pnas.0404915101 PMC515119

[wsbm1570-bib-0192] Nassini, R. , Gees, M. , Harrison, S. , de Siena, G. , Materazzi, S. , Moretto, N. , Failli, P. , Preti, D. , Marchetti, N. , Cavazzini, A. , Mancini, F. , Pedretti, P. , Nilius, B. , Patacchini, R. , & Geppetti, P. (2011). Oxaliplatin elicits mechanical and cold allodynia in rodents via TRPA1 receptor stimulation. Pain, 152(7), 1621–1631. 10.1016/J.PAIN.2011.02.051 21481532

[wsbm1570-bib-0193] Navratilova, E. , Fillingim, R. B. , & Porreca, F. (2021). Sexual dimorphism in functional pain syndromes. Science Translational Medicine, 13(619), 1–5. 10.1126/SCITRANSLMED.ABJ7180 PMC12180430 34757805

[wsbm1570-bib-0194] Neely, G. G. , Hess, A. , Costigan, M. , Keene, A. C. , Goulas, S. , Langeslag, M. , Griffin, R. S. , Belfer, I. , Dai, F. , Smith, S. B. , Diatchenko, L. , Gupta, V. , Xia, C. P. , Amann, S. , Kreitz, S. , Heindl‐Erdmann, C. , Wolz, S. , Ly, C. V. , Arora, S. , … Penninger, J. M. (2010). A genome‐wide drosophila screen for heat nociception identifies α2δ3 as an evolutionarily conserved pain gene. Cell, 143(4), 628–638. 10.1016/J.CELL.2010.09.047 21074052 PMC3040441

[wsbm1570-bib-0195] Neves, S. R. , Ram, P. T. , & Iyengar, R. (2002). G protein pathways. Science, 296(5573), 1636–1639. 10.1126/SCIENCE.1071550 12040175

[wsbm1570-bib-0196] Nicol, L. S. C. , Thornton, P. , Hatcher, J. P. , Glover, C. P. , Webster, C. I. , Burrell, M. , Hammett, K. , Jones, C. A. , Sleeman, M. A. , Billinton, A. , & Chessell, I. (2018). Central inhibition of granulocyte‐macrophage colony‐stimulating factor is analgesic in experimental neuropathic pain. Pain, 159(3), 550–559. 10.1097/J.PAIN.0000000000001130 29351125 PMC5828377

[wsbm1570-bib-0197] Nissenbaum, J. , Devor, M. , Seltzer, Z. , Gebauer, M. , Michaelis, M. , Tal, M. , Dorfman, R. , Abitbul‐Yarkoni, M. , Lu, Y. , Elahipanah, T. , delCanho, S. , Minert, A. , Fried, K. , Persson, A.‐K. , Shpigler, H. , Shabo, E. , Yakir, B. , Pisanté, A. , & Darvasi, A. (2010). Susceptibility to chronic pain following nerve injury is genetically affected by CACNG2. Genome Research, 20(9), 1180–1190. 10.1101/GR.104976.110 20688780 PMC2928496

[wsbm1570-bib-0198] Norões, M. M. , Santos, L. G. , Gavioli, E. C. , de Paula Soares Rachetti, V. , Otuki, M. F. , de Almeida Cabrini, D. , da Silveira Prudente, A. , Oliveira, J. R. J. M. , de Carvalho Gonçalves, M. , Ferreira, J. , Preti, D. , de Logu, F. , Nassini, R. , & André, E. (2019). Role of TRPA1 receptors in skin inflammation induced by volatile chemical irritants in mice. European Journal of Pharmacology, 858, 172460. 10.1016/J.EJPHAR.2019.172460 31228448

[wsbm1570-bib-0199] Nozaki, C. , le Bourdonnec, B. , Reiss, D. , Windh, R. T. , Little, P. J. , Dolle, R. E. , Kieffer, B. L. , & Gavériaux‐Ruff, C. (2012). δ‐Opioid mechanisms for ADL5747 and ADL5859 effects in mice: Analgesia, locomotion, and receptor internalization. Journal of Pharmacology and Experimental Therapeutics, 342(3), 799–807. 10.1124/JPET.111.188987 22700431 PMC3422521

[wsbm1570-bib-0200] O'Brien, D. E. , Alter, B. J. , Satomoto, M. , Morgan, C. D. , Davidson, S. , Vogt, S. K. , Norman, M. E. , Gereau, G. B. , Demaro, J. A. , Landreth, G. E. , Golden, J. P. , & Gereau, R. W. (2015). ERK2 alone drives inflammatory pain but cooperates with ERK1 in sensory neuron survival. Journal of Neuroscience, 35(25), 9491–9507. 10.1523/JNEUROSCI.4404-14.2015 26109671 PMC4478259

[wsbm1570-bib-0201] Okuda, T. , van Deursen, J. , Hiebert, S. W. , Grosveld, G. , & Downing, J. R. (1996). AML1, the target of multiple chromosomal translocations in human leukemia, is essential for Normal fetal liver hematopoiesis. Cell, 84(2), 321–330. 10.1016/S0092-8674(00)80986-1 8565077

[wsbm1570-bib-0202] Otsubo, Y. , Satoh, Y. , Kodama, M. , Araki, Y. , Satomoto, M. , Sakamoto, E. , Pagès, G. , Pouysségur, J. , Endo, S. , & Kazama, T. (2012). Mechanical allodynia but not thermal hyperalgesia is impaired in mice deficient for ERK2 in the central nervous system. Pain, 153(11), 2241–2252. 10.1016/J.PAIN.2012.07.020 22902213

[wsbm1570-bib-0203] Ozaktay, A. C. , Kallakuri, S. , Takebayashi, T. , Cavanaugh, J. M. , Asik, I. , DeLeo, J. A. , & Weinstein, J. N. (2006). Effects of interleukin‐1 beta, interleukin‐6, and tumor necrosis factor on sensitivity of dorsal root ganglion and peripheral receptive fields in rats. European Spine Journal, 15(10), 1529–1537. 10.1007/S00586-005-0058-8 16474945

[wsbm1570-bib-0204] Özdoǧan, Ü. K. , Lähdesmäki, J. , & Scheinin, M. (2006). The analgesic efficacy of partial opioid agonists is increased in mice with targeted inactivation of the α2A‐adrenoceptor gene. European Journal of Pharmacology, 529(1–3), 105–113. 10.1016/J.EJPHAR.2005.10.029 16325800

[wsbm1570-bib-0205] Pakos‐Zebrucka, K. , Koryga, I. , Mnich, K. , Ljujic, M. , Samali, A. , & Gorman, A. M. (2016). The integrated stress response. EMBO Reports, 17(10), 1374. 10.15252/EMBR.201642195 27629041 PMC5048378

[wsbm1570-bib-0206] Palazzo, E. , Fu, Y. , Ji, G. , Maione, S. , & Neugebauer, V. (2008). Group III mGluR7 and mGluR8 in the amygdala differentially modulate nocifensive and affective pain behaviors. Neuropharmacology, 55(4), 537–545. 10.1016/J.NEUROPHARM.2008.05.007 18533199 PMC2601632

[wsbm1570-bib-0207] Palma, C. , & Manzini, S. (1998). Substance P induces secretion of immunomodulatory cytokines by human astrocytoma cells. Journal of Neuroimmunology, 81(1–2), 127–137. 10.1016/S0165-5728(97)00167-7 9521614

[wsbm1570-bib-0208] Pan, H.‐L. , Wu, Z.‐Z. , Zhou, H.‐Y. , Chen, S.‐R. , Zhang, H.‐M. , & Li, D.‐P. (2008). Modulation of pain transmission by G protein‐coupled receptors. Pharmacology & Therapeutics, 117(1), 141. 10.1016/J.PHARMTHERA.2007.09.003 17959251 PMC2965406

[wsbm1570-bib-0209] Park, J. F. , & Luo, Z. D. (2010). Calcium channel functions in pain processing. Channels, 4(6), 510. 10.4161/CHAN.4.6.12869 21150297 PMC3052250

[wsbm1570-bib-0210] Patel, R. , Bauer, C. S. , Nieto‐Rostro, M. , Margas, W. , Ferron, L. , Chaggar, K. , Crews, K. , Ramirez, J. D. , Bennett, D. L. H. , Schwartz, A. , Dickenson, A. H. , & Dolphin, A. C. (2013). α2δ‐1 gene deletion affects somatosensory neuron function and delays mechanical hypersensitivity in response to peripheral nerve damage. Journal of Neuroscience, 33(42), 16412–16426. 10.1523/JNEUROSCI.1026-13.2013 24133248 PMC3797367

[wsbm1570-bib-0211] Peirs, C. , & Seal, R. P. (2016). Neural circuits for pain: Recent advances and current views. Science (New York, NY), 354(6312), 578–584. 10.1126/SCIENCE.AAF8933 PMC11327866 27811268

[wsbm1570-bib-0212] Peirs, C. , Williams, S.‐P. G. , Zhao, X. , Walsh, C. E. , Gedeon, J. Y. , Cagle, N. E. , Goldring, A. C. , Hioki, H. , Liu, Z. , Marell, P. S. , & Seal, R. P. (2015). Dorsal horn circuits for persistent mechanical pain. Neuron, 87(4), 797. 10.1016/J.NEURON.2015.07.029 26291162 PMC4562334

[wsbm1570-bib-0213] Pereira, V. , Millet, Q. , Aramburu, J. , Lopez‐Rodriguez, C. , Gaveriaux‐Ruff, C. , & Wood, J. N. (2018). Analgesia linked to Nav1.7 loss of function requires micro‐ and δ‐opioid receptors. Wellcome Open Research, 3, 101. doi:10.12688/wellcomeopenres.14687.1 30271888 PMC6134336

[wsbm1570-bib-0214] Pesquero, J. B. , Araujo, R. C. , Heppenstall, P. A. , Stucky, C. L. , Silva, J. A. , Walther, T. , Oliveira, S. M. , Pesquero, J. L. , Paiva, A. C. M. , Calixto, J. B. , Lewin, G. R. , & Bader, M. (2000). Hypoalgesia and altered inflammatory responses in mice lacking kinin B1 receptors. Proceedings of the National Academy of Sciences, 97(14), 8140–8145. 10.1073/PNAS.120035997 PMC16683 10859349

[wsbm1570-bib-0215] Petrenko, A. B. , Yamakura, T. , Baba, H. , & Shimoji, K. (2003). The role of N‐methyl‐D‐aspartate (NMDA) receptors in pain: A review. Anesthesia and Analgesia, 97(4), 1108–1116. 10.1213/01.ANE.0000081061.12235.55 14500166

[wsbm1570-bib-0216] Pinho‐Ribeiro, F. A. , Verri, W. A. , & Chiu, I. M. (2017). Nociceptor sensory neuron–immune interactions in pain and inflammation. Trends in Immunology, 38(1), 5–19. 10.1016/J.IT.2016.10.001 27793571 PMC5205568

[wsbm1570-bib-0217] Plenge, R. M. , Scolnick, E. M. , & Altshuler, D. (2013). Validating therapeutic targets through human genetics. Nature Reviews. Drug Discovery, 12(8), 581–594. 10.1038/NRD4051 23868113

[wsbm1570-bib-0218] Prescott, S. A. , & Ratté, S. (2017). Somatosensation and pain. Conn's Translational Neuroscience, 1, 517–539. 10.1016/B978-0-12-802381-5.00037-3

[wsbm1570-bib-0219] Priest, B. T. , Murphy, B. A. , Lindia, J. A. , Diaz, C. , Abbadie, C. , Ritter, A. M. , Liberator, P. , Iyer, L. M. , Kash, S. F. , Kohler, M. G. , Kaczorowski, G. J. , MacIntyre, D. E. , & Martin, W. J. (2005). Contribution of the tetrodotoxin‐resistant voltage‐gated sodium channel NaV1.9 to sensory transmission and nociceptive behavior. Proceedings of the National Academy of Sciences of the United States of America, 102(26), 9382–9387. 10.1073/PNAS.0501549102 15964986 PMC1166597

[wsbm1570-bib-0220] Puma, S. L. , Landini, L. , Macedo, S. J. , Seravalli, V. , Marone, I. M. , Coppi, E. , Patacchini, R. , Geppetti, P. , Materazzi, S. , Nassini, R. , & de Logu, F. (2019). TRPA1 mediates the antinociceptive properties of the constituent of *Crocus sativus* L., safranal. Journal of Cellular and Molecular Medicine, 23(3), 1976–1986. 10.1111/JCMM.14099 30636360 PMC6378183

[wsbm1570-bib-0221] R Core Team . (2017). R: A language and environment for statistical computing. R Foundation for Statistical Computing.

[wsbm1570-bib-0222] R. Eid, S. (2011). Therapeutic targeting of TRP channels—The TR(i)P to pain relief. Current Topics in Medicinal Chemistry, 11(17), 2118–2130. 10.2174/156802611796904898 21671881

[wsbm1570-bib-0223] Raithel, S. J. , Sapio, M. R. , LaPaglia, D. M. , Iadarola, M. J. , & Mannes, A. J. (2018). Transcriptional changes in dorsal spinal cord persist after surgical incision despite preemptive analgesia with peripheral resiniferatoxin. Anesthesiology, 128(3), 620–635. 10.1097/ALN.0000000000002006 29271803 PMC11175836

[wsbm1570-bib-0224] Ramachandran, R. , Wang, Z. , Saavedra, C. , DiNardo, A. , Corr, M. , Powell, S. B. , & Yaksh, T. L. (2019). Role of Toll‐like receptor 4 signaling in mast cell‐mediated migraine pain pathway, Molecular Pain, 15, 1744806919867842. 10.1177/1744806919867842 31342858 PMC6688145

[wsbm1570-bib-0225] Ramer, M. S. , Murphy, P. G. , Richardson, P. M. , & Bisby, M. A. (1998). Spinal nerve lesion‐induced mechanoallodynia and adrenergic sprouting in sensory ganglia are attenuated in interleukin‐6 knockout mice. Pain, 78(2), 115–121. 10.1016/S0304-3959(98)00121-3 9839821

[wsbm1570-bib-0226] Rameshwar, P. , Gascon, P. , & Ganea, D. (1992). Immunoregulatory effects of neuropeptides. Stimulation of interleukin‐2 production by substance P. Journal of Neuroimmunology, 37(1–2), 65–74. 10.1016/0165-5728(92)90156-F 1372331

[wsbm1570-bib-0227] Ray, P. , Torck, A. , Quigley, L. , Wangzhou, A. , Neiman, M. , Rao, C. , Lam, T. , Kim, J.‐Y. , Kim, T. H. , Zhang, M. Q. , Dussor, G. , & Price, T. J. (2018). Comparative transcriptome profiling of the human and mouse dorsal root ganglia: An RNA‐seq‐based resource for pain and sensory neuroscience research. Pain, 159(7), 1325. 10.1097/J.PAIN.0000000000001217 29561359 PMC6008200

[wsbm1570-bib-0228] Ren, W. , & Neugebauer, V. (2010). Pain‐related increase of excitatory transmission and decrease of inhibitory transmission in the central nucleus of the amygdala are mediated by mGluR1. Molecular Pain, 6(1), 1–14. 10.1186/1744-8069-6-93 21162731 PMC3016348

[wsbm1570-bib-0229] Ren, W. , Palazzo, E. , Maione, S. , & Neugebauer, V. (2011). Differential effects of mGluR7 and mGluR8 activation on pain‐related synaptic activity in the amygdala. Neuropharmacology, 61(8), 1334–1344. 10.1016/J.NEUROPHARM.2011.08.006 21854791 PMC3189345

[wsbm1570-bib-0230] Replogle, J. M. , Norman, T. M. , Xu, A. , Hussmann, J. A. , Chen, J. , Cogan, J. Z. , Meer, E. J. , Terry, J. M. , Riordan, D. P. , Srinivas, N. , Fiddes, I. T. , Arthur, J. G. , Alvarado, L. J. , Pfeiffer, K. A. , Mikkelsen, T. S. , Weissman, J. S. , & Adamson, B. (2020). Combinatorial single‐cell CRISPR screens by direct guide RNA capture and targeted sequencing. Nature Biotechnology, 38(8), 954–961. 10.1038/s41587-020-0470-y PMC7416462 32231336

[wsbm1570-bib-0231] Robinson, P. K. (2015). Enzymes: Principles and biotechnological applications. Essays in Biochemistry, 59, 1. 10.1042/BSE0590001 26504249 PMC4692135

[wsbm1570-bib-0232] RStudio Team . (2020). RStudio: Integrated development environment for R. R Foundation for Statistical Computing.

[wsbm1570-bib-0233] Rupniak, N. M. J. , Boyce, S. , Webb, J. K. , Williams, A. R. , Carlson, E. J. , Hill, R. G. , Borkowski, J. A. , & Hess, J. F. (1997). Effects of the bradykinin B1 receptor antagonist des‐ Arg9[Leu8]bradykinin and genetic disruption of the B2 receptor on nociception in rats and mice. Pain, 71(1), 89–97. 10.1016/S0304-3959(97)03343-5 9200178

[wsbm1570-bib-0234] Russo, A. F. (2017). Overview of neuropeptides: Awakening the senses? Headache: The Journal of Head and Face Pain, 57, 37–46. 10.1111/HEAD.13084 PMC5424629 28485842

[wsbm1570-bib-0235] Sadja, R. , Alagem, N. , & Reuveny, E. (2003). Gating of GIRK channels: Details of an intricate, membrane‐delimited signaling complex. Neuron, 39(1), 9–12. 10.1016/S0896-6273(03)00402-1 12848928

[wsbm1570-bib-0236] Sadler, K. E. , Moehring, F. , Shiers, S. I. , Laskowski, L. J. , Mikesell, A. R. , Plautz, Z. R. , Brezinski, A. N. , Mecca, C. M. , Dussor, G. , Price, T. J. , McCorvy, J. D. , & Stucky, C. L. (2021). Transient receptor potential canonical 5 mediates inflammatory mechanical and spontaneous pain in mice. Science Translational Medicine, 13(595), 1–17. 10.1126/SCITRANSLMED.ABD7702 PMC8923002 34039739

[wsbm1570-bib-0237] Saegusa, H. , Kurihara, T. , Zong, S. , Kazuno, A. , Matsuda, Y. , Nonaka, T. , Han, W. , Toriyama, H. , & Tanabe, T. (2001). Suppression of inflammatory and neuropathic pain symptoms in mice lacking the N‐type Ca^2+^ channel. The EMBO Journal, 20(10), 2349–2356. 10.1093/EMBOJ/20.10.2349 11350923 PMC125247

[wsbm1570-bib-0238] Sahbaie, P. , Shi, X. , Guo, T. Z. , Qiao, Y. , Yeomans, D. C. , Kingery, W. S. , & Clark, J. D. (2009). Role of substance P signaling in enhanced nociceptive sensitization and local cytokine production after incision. Pain, 145(3), 341–349. 10.1016/J.PAIN.2009.06.037 19660865 PMC2746201

[wsbm1570-bib-0239] Sapio, M. R. , Iadarola, M. J. , LaPaglia, D. M. , Lehky, T. , Thurm, A. E. , Danley, K. M. , Fuhr, S. R. , Lee, M. D. , Huey, A. E. , Sharp, S. J. , Tsao, J. W. , Yanovski, J. A. , Mannes, A. J. , & Han, J. C. (2019). Haploinsufficiency of the brain‐derived neurotrophic factor gene is associated with reduced pain sensitivity. Pain, 160(5), 1070–1081. 10.1097/J.PAIN.0000000000001485 30855519 PMC6476691

[wsbm1570-bib-0240] Sarkar, S. , Aziz, Q. , Woolf, C. J. , Hobson, A. R. , & Thompson, D. G. (2000). Contribution of central sensitisation to the development of noncardiac chest pain. The Lancet, 356(9236), 1154–1159. 10.1016/S0140-6736(00)02758-6 11030295

[wsbm1570-bib-0241] Sassone‐Corsi, P. (2012). The cyclic AMP pathway. Cold Spring Harbor Perspectives in Biology, 4(12), 1–4. 10.1101/CSHPERSPECT.A011148 PMC3504441 23209152

[wsbm1570-bib-0242] Schepers, R. J. , Mahoney, J. L. , Gehrke, B. J. , & Shippenberg, T. S. (2008). Endogenous kappa‐opioid receptor systems inhibit hyperalgesia associated with localized peripheral inflammation. Pain, 138(2), 423–439. 10.1016/J.PAIN.2008.01.023 18355964 PMC2553515

[wsbm1570-bib-0243] Scholz, J. (2014). Mechanisms of chronic pain. Molecular Pain, 10(Suppl 1), O15. 10.1186/1744-8069-10-S1-O15

[wsbm1570-bib-0244] Seino, D. , Tokunaga, A. , Tachibana, T. , Yoshiya, S. , Dai, Y. , Obata, K. , Yamanaka, H. , Kobayashi, K. , & Noguchi, K. (2006). The role of ERK signaling and the P2X receptor on mechanical pain evoked by movement of inflamed knee joint. Pain, 123(1–2), 193–203. 10.1016/J.PAIN.2006.02.032 16616417

[wsbm1570-bib-0245] Shaye, H. , Stauch, B. , Gati, C. , & Cherezov, V. (2021). Molecular mechanisms of metabotropic GABAB receptor function. Science Advances, 7(22), 3362–3390. 10.1126/SCIADV.ABG3362 PMC8163086 34049877

[wsbm1570-bib-0246] Shields, S. D. , Deng, L. , Reese, R. M. , Dourado, M. , Tao, J. , Foreman, O. , Chang, J. H. , & Hackos, D. H. (2018). Insensitivity to pain upon adult‐onset deletion of Nav1.7 or its blockade with selective inhibitors. Journal of Neuroscience, 38(47), 10180–10201. 10.1523/JNEUROSCI.1049-18.2018 30301756 PMC6596201

[wsbm1570-bib-0247] Shukla, T. N. , Song, J. , & Campbell, Z. T. (2020). Molecular entrapment by RNA: An emerging tool for disrupting protein–RNA interactions in vivo. RNA Biology, 17(4), 417–424. 10.1080/15476286.2020.1717059 31957541 PMC7237136

[wsbm1570-bib-0248] Sigworth, F. J. (1994). Voltage gating of ion channels. Quarterly Reviews of Biophysics, 27(1), 1–40. 10.1017/S0033583500002894 7520590

[wsbm1570-bib-0249] Silva, C. R. , Oliveira, S. M. , Hoffmeister, C. , Funck, V. , Guerra, G. P. , Trevisan, G. , Tonello, R. , Rossato, M. F. , Pesquero, J. B. , Bader, M. , Oliveira, M. S. , McDougall, J. J. , & Ferreira, J. (2016). The role of kinin B1 receptor and the effect of angiotensin I‐converting enzyme inhibition on acute gout attacks in rodents. Annals of the Rheumatic Diseases, 75(1), 260–268. 10.1136/ANNRHEUMDIS-2014-205739 25344431

[wsbm1570-bib-0250] Simonin, F. , Valverde, O. , Smadja, C. , Slowe, S. , Kitchen, I. , Dierich, A. , le Meur, M. , Roques, B. P. , Maldonado, R. , & Kieffer, B. L. (1998). Disruption of the κ‐opioid receptor gene in mice enhances sensitivity to chemical visceral pain, impairs pharmacological actions of the selective κ‐agonist U‐50,488H and attenuates morphine withdrawal. The EMBO Journal, 17(4), 886–897. 10.1093/EMBOJ/17.4.886 9463367 PMC1170438

[wsbm1570-bib-0251] Smeyne, R. J. , Klein, R. , Schnapp, A. , Long, L. K. , Bryant, S. , Lewin, A. , Lira, S. A. , & Barbacid, M. (1994). Severe sensory and sympathetic neuropathies in mice carrying a disrupted Trk/NGF receptor gene. Nature, 368(6468), 246–249. 10.1038/368246a0 8145823

[wsbm1570-bib-0252] Song, X.‐S. , Cao, J.‐L. , Xu, Y.‐B. , He, J.‐H. , Zhang, L.‐C. , & Zeng, Y.‐M. (2005). Activation of ERK/CREB pathway in spinal cord contributes to chronic constrictive injury‐induced neuropathic pain in rats 1. Acta Pharmacologica Sinica, 26(7), 789–798. 10.1111/j.1745-7254.2005.00123.x 15960884

[wsbm1570-bib-0253] Sora, I. , Takahashi, N. , Funada, M. , Ujike, H. , Revay, R. S. , Donovan, D. M. , Miner, L. L. , & Uhl, G. R. (1997). Opiate receptor knockout mice define μ receptor roles in endogenous nociceptive responses and morphine‐induced analgesia. Proceedings of the National Academy of Sciences of the United States of America, 94(4), 1544–1549. 10.1073/PNAS.94.4.1544 9037090 PMC19828

[wsbm1570-bib-0254] Sorkin, L. S. , Boyle, D. L. , Hammaker, D. , Herman, D. S. , Vail, E. , & Firestein, G. S. (2009). MKK3, an upstream activator of p38, contributes to formalin phase 2 and late allodynia in mice. Neuroscience, 162(2), 462–471. 10.1016/J.NEUROSCIENCE.2009.05.008 19427893 PMC2802055

[wsbm1570-bib-0255] South, S. M. , Kohno, T. , Kaspar, B. K. , Hegarty, D. , Vissel, B. , Drake, C. T. , Ohata, M. , Jenab, S. , Sailer, A. W. , Malkmus, S. , Masuyama, T. , Horner, P. , Bogulavsky, J. , Gage, F. H. , Yaksh, T. L. , Woolf, C. J. , Heinemann, S. F. , & Inturrisi, C. E. (2003). A conditional deletion of the NR1 subunit of the NMDA receptor in adult spinal cord dorsal horn reduces NMDA currents and injury‐induced pain. Journal of Neuroscience, 23(12), 5031–5040. 10.1523/JNEUROSCI.23-12-05031.2003 12832526 PMC6741202

[wsbm1570-bib-0256] Spencer, N. J. , Magnúsdóttir, E. I. , Jakobsson, J. E. T. , Kestell, G. , Chen, B. N. , Morris, D. , Brookes, S. J. , & Lagerström, M. C. (2018). CGRPα within the Trpv1‐Cre population contributes to visceral nociception. American Journal of Physiology. Gastrointestinal and Liver Physiology, 314(2), G188–G200. doi:10.1152/Ajpgi.00188.2017. 10.1152/Ajpgi.00188.2017 28971837

[wsbm1570-bib-0257] Sreenivasan, L. , Wang, H. , Yap, S. Q. , Leclair, P. , Tam, A. , & Lim, C. J. (2020). Autocrine IL‐6/STAT3 signaling aids development of acquired drug resistance in group 3 medulloblastoma. Cell Death & Disease, 11(12), 1–15. 10.1038/s41419-020-03241-y 33279931 PMC7719195

[wsbm1570-bib-0258] Stefanoska, K. , Bertz, J. , Volkerling, A. M. , van der Hoven, J. , Ittner, L. M. , & Ittner, A. (2018). Neuronal MAP kinase p38α inhibits c‐Jun N‐terminal kinase to modulate anxiety‐related behaviour. Scientific Reports, 8(1), 1–12. 10.1038/s41598-018-32592-y 30250211 PMC6155170

[wsbm1570-bib-0259] Stirling, L. C. , Forlani, G. , Baker, M. D. , Wood, J. N. , Matthews, E. A. , Dickenson, A. H. , & Nassar, M. A. (2005). Nociceptor‐specific gene deletion using heterozygous Na V1.8‐Cre recombinase mice. Pain, 113(1–2), 27–36. 10.1016/J.PAIN.2004.08.015 15621361

[wsbm1570-bib-0260] Stone, L. S. , MacMillan, L. B. , Kitto, K. F. , Limbird, L. E. , & Wilcox, G. L. (1997). The α2a adrenergic receptor subtype mediates spinal analgesia evoked by α2 agonists and is necessary for spinal adrenergic–opioid synergy. Journal of Neuroscience, 17(18), 7157–7165. 10.1523/JNEUROSCI.17-18-07157.1997 9278550 PMC6573259

[wsbm1570-bib-0261] Szabó, Á. , Helyes, Z. , Sándor, K. , Bite, A. , Pintér, E. , Németh, J. , Bánvölgyi, Á. , Bölcskei, K. , Elekes, K. , & Szolcsányi, J. (2005). Role of transient receptor potential Vanilloid 1 receptors in adjuvant‐induced chronic arthritis: In vivo study using gene‐deficient mice. Journal of Pharmacology and Experimental Therapeutics, 314(1), 111–119. 10.1124/JPET.104.082487 15831443

[wsbm1570-bib-0262] Treede, R. D. , Meyer, R. A. , Raja, S. N. , & Campbell, J. N. (1992). Peripheral and central mechanisms of cutaneous hyperalgesia. Progress in Neurobiology, 38(4), 397–421. 10.1016/0301-0082(92)90027-C 1574584

[wsbm1570-bib-0263] Trevisan, G. , Hoffmeister, C. , Rossato, M. F. , Oliveira, S. M. , Silva, M. A. , Ineu, R. P. , Guerra, G. P. , Materazzi, S. , Fusi, C. , Nassini, R. , Geppetti, P. , & Ferreira, J. (2013). Transient receptor potential Ankyrin 1 receptor stimulation by hydrogen peroxide is critical to trigger pain during monosodium urate–induced inflammation in rodents. Arthritis and Rheumatism, 65(11), 2984–2995. 10.1002/ART.38112 23918657

[wsbm1570-bib-0264] Trevisan, G. , Hoffmeister, C. , Rossato, M. F. , Oliveira, S. M. , Silva, M. A. , Silva, C. R. , Fusi, C. , Tonello, R. , Minocci, D. , Guerra, G. P. , Materazzi, S. , Nassini, R. , Geppetti, P. , & Ferreira, J. (2014). TRPA1 receptor stimulation by hydrogen peroxide is critical to trigger hyperalgesia and inflammation in a model of acute gout. Free Radical Biology and Medicine, 72, 200–209. 10.1016/J.FREERADBIOMED.2014.04.021 24780252

[wsbm1570-bib-0265] Tsantoulas, C. , Denk, F. , Signore, M. , Nassar, M. A. , Futai, K. , & McMahon, S. B. (2018). Mice lacking Kcns1 in peripheral neurons show increased basal and neuropathic pain sensitivity. Pain, 159(8), 1641–1651. 10.1097/J.PAIN.0000000000001255 29697531 PMC6053330

[wsbm1570-bib-0266] Tyler, W. J. , Perrett, S. P. , & Pozzo‐Miller, L. D. (2002). The role of neurotrophins in neurotransmitter release. The Neuroscientist, 8(6), 524–531. 10.1177/1073858402238511 12467374 PMC2810653

[wsbm1570-bib-0267] Usoskin, D. , Furlan, A. , Islam, S. , Abdo, H. , Lönnerberg, P. , Lou, D. , Hjerling‐Leffler, J. , Haeggström, J. , Kharchenko, O. , Kharchenko, P. V. , Linnarsson, S. , & Ernfors, P. (2014). Unbiased classification of sensory neuron types by large‐scale single‐cell RNA sequencing. Nature Neuroscience, 18(1), 145–153. 10.1038/nn.3881 25420068

[wsbm1570-bib-0268] Uttam, S. , Wong, C. , Amorim, I. S. , Jafarnejad, S. M. , Tansley, S. N. , Yang, J. , Prager‐Khoutorsky, M. , Mogil, J. S. , Gkogkas, C. G. , & Khoutorsky, A. (2018). Translational profiling of dorsal root ganglia and spinal cord in a mouse model of neuropathic pain. Neurobiology of Pain, 4, 35. 10.1016/J.YNPAI.2018.04.001 30906902 PMC6428075

[wsbm1570-bib-0269] Vasudevan, D. , Neuman, S. D. , Yang, A. , Lough, L. , Brown, B. , Bashirullah, A. , Cardozo, T. , & Ryoo, H. D. (2020). Translational induction of ATF4 during integrated stress response requires noncanonical initiation factors eIF2D and DENR. Nature Communications, 11(1), 1–11. 10.1038/s41467-020-18453-1 PMC7495428 32938929

[wsbm1570-bib-0270] Vergnolle, N. , Bunnett, N. W. , Sharkey, K. A. , Brussee, V. , Compton, S. J. , Grady, E. F. , Cirino, G. , Gerard, N. , Basbaum, A. I. , Andrade‐Gordon, P. , Hollenberg, M. D. , & Wallace, J. L. (2001). Proteinase‐activated receptor‐2 and hyperalgesia: A novel pain pathway. Nature Medicine, 7(7), 821–826. 10.1038/89945 11433347

[wsbm1570-bib-0271] Wainger, B. J. , Buttermore, E. D. , Oliveira, J. T. , Mellin, C. , Lee, S. , Saber, W. A. , Wang, A. , Ichida, J. K. , Chiu, I. M. , Barrett, L. , Huebner, E. A. , Bilgin, C. , Tsujimoto, N. , Brenneis, C. , Kapur, K. , Rubin, L. L. , Eggan, K. , & Woolf, C. J. (2015). Modeling pain in vitro using nociceptor neurons reprogrammed from fibroblasts. Nature Neuroscience, 18(1), 17. 10.1038/NN.3886 25420066 PMC4429606

[wsbm1570-bib-0272] Walker, K. , Reeve, A. , Bowes, M. , Winter, J. , Wotherspoon, G. , Davis, A. , Schmid, P. , Gasparini, F. , Kuhn, R. , & Urban, L. (2001). mGlu5 receptors and nociceptive function II. mGlu5 receptors functionally expressed on peripheral sensory neurones mediate inflammatory hyperalgesia. Neuropharmacology, 40(1), 10–19. 10.1016/S0028-3908(00)00114-3 11077066

[wsbm1570-bib-0273] Wang, Q. , Stacy, T. , Binder, M. , Marin‐Padilla, M. , Sharpe, A. H. , & Speck, N. A. (1996). Disruption of the Cbfa2 gene causes necrosis and hemorrhaging in the central nervous system and blocks definitive hematopoiesis. Proceedings of the National Academy of Sciences, 93(8), 3444–3449. 10.1073/PNAS.93.8.3444 PMC39628 8622955

[wsbm1570-bib-0274] Wang, Z. , Gardell, L. R. , Ossipov, M. H. , Vanderah, T. W. , Brennan, M. B. , Hochgeschwender, U. , Hruby, V. J. , Malan, T. P. , Lai, J. , & Porreca, F. (2001). Pronociceptive actions of Dynorphin maintain chronic neuropathic pain. Journal of Neuroscience, 21(5), 1779–1786. 10.1523/JNEUROSCI.21-05-01779.2001 11222667 PMC6762963

[wsbm1570-bib-0275] Wardill, H. R. , Gibson, R. J. , van Sebille, Y. Z. A. , Secombe, K. R. , Coller, J. K. , White, I. A. , Manavis, J. , Hutchinson, M. R. , Staikopoulos, V. , Logan, R. M. , & Bowen, J. M. (2016). Irinotecan‐induced gastrointestinal dysfunction and pain are mediated by common TLR4‐dependent mechanisms. Molecular Cancer Therapeutics, 15(6), 1376–1386. 10.1158/1535-7163.MCT-15-0990 27197307

[wsbm1570-bib-0276] Wei, F. , Wang, G.‐D. , Kerchner, G. A. , Kim, S. J. , Xu, H.‐M. , Chen, Z.‐F. , & Zhuo, M. (2001). Genetic enhancement of inflammatory pain by forebrain NR2B overexpression. Nature Neuroscience, 4(2), 164–169. 10.1038/83993 11175877

[wsbm1570-bib-0277] Weibel, R. , Reiss, D. , Karchewski, L. , Gardon, O. , Matifas, A. , Filliol, D. , Becker, J. A. J. , Wood, J. N. , Kieffer, B. L. , & Gaveriaux‐Ruff, C. (2013). Mu opioid receptors on primary afferent Nav1.8 neurons contribute to opiate‐induced analgesia: Insight from conditional knockout mice. PLoS One, 8(9), e74706. 10.1371/JOURNAL.PONE.0074706 24069332 PMC3771900

[wsbm1570-bib-0278] Weyerbacher, A. R. , Xu, Q. , Tamasdan, C. , Shin, S. J. , & Inturrisi, C. E. (2010). N‐methyl‐d‐aspartate receptor (NMDAR) independent maintenance of inflammatory pain. Pain, 148(2), 237–246. 10.1016/J.PAIN.2009.11.003 20005044 PMC2831745

[wsbm1570-bib-0279] Woolf, C. J. (1983). Evidence for a central component of post‐injury pain hypersensitivity. Nature, 306(5944), 686–688. 10.1038/306686a0 6656869

[wsbm1570-bib-0280] Woolf, C. J. (2020). Capturing novel non‐opioid pain targets. Biological Psychiatry, 87(1), 74–81. 10.1016/J.BIOPSYCH.2019.06.017 31399256 PMC6898770

[wsbm1570-bib-0281] Woolf, C. J. , & Ma, Q. (2007). Nociceptors—Noxious stimulus detectors. Neuron, 55(3), 353–364. 10.1016/J.NEURON.2007.07.016 17678850

[wsbm1570-bib-0282] Woolf, C. J. , & Thompson, S. W. N. (1991). The induction and maintenance of central sensitization is dependent on N‐methyl‐d‐aspartic acid receptor activation; implications for the treatment of post‐injury pain hypersensitivity states. Pain, 44(3), 293–299. 10.1016/0304-3959(91)90100-C 1828878

[wsbm1570-bib-0283] Wu, D. , Katz, A. , & Simon, M. I. (1993). Activation of phospholipase C β2 by the α and βγ subunits of trimeric GTP‐binding protein. Proceedings of the National Academy of Sciences of the United States of America, 90(11), 5297–5301. 10.1073/PNAS.90.11.5297 8389480 PMC46703

[wsbm1570-bib-0284] Wu, W. P. , Hao, J. X. , Halldner‐Henriksson, L. , Xu, X. J. , Jacobson, M. A. , Wiesenfeld‐Hallin, Z. , & Fredholm, B. B. (2002). Decreased inflammatory pain due to reduced carrageenan‐induced inflammation in mice lacking adenosine A3 receptors. Neuroscience, 114(3), 523–527. 10.1016/S0306-4522(02)00273-7 12220556

[wsbm1570-bib-0285] Xie, M.‐X. , Cao, X.‐Y. , Zeng, W.‐A. , Lai, R.‐C. , Guo, L. , Wang, J.‐C. , Xiao, Y.‐B. , Zhang, X. , Chen, D. , Liu, X.‐G. , & Zhang, X.‐L. (2021). ATF4 selectively regulates heat nociception and contributes to kinesin‐mediated TRPM3 trafficking. Nature Communications, 12(1), 1–18. 10.1038/s41467-021-21731-1 PMC7930092 33658516

[wsbm1570-bib-0286] Xie, Z. , Bailey, A. , Kuleshov, M. V. , Clarke, D. J. B. , Evangelista, J. E. , Jenkins, S. L. , Lachmann, A. , Wojciechowicz, M. L. , Kropiwnicki, E. , Jagodnik, K. M. , Jeon, M. , & Ma'ayan, A. (2021). Gene set knowledge discovery with Enrichr. Current Protocols, 1(3), e90. 10.1002/CPZ1.90 33780170 PMC8152575

[wsbm1570-bib-0287] Xu, X. J. , Hao, J. X. , Andell‐Jonsson, S. , Poli, V. , Bartfai, T. , & Wiesenfeld‐Hallin, Z. (1997). Nociceptive responses in INTERLEUKIN‐6‐deficient mice to peripheral inflammation and peripheral nerve section. Cytokine, 9(12), 1028–1033. 10.1006/CYTO.1997.0243 9417815

[wsbm1570-bib-0288] Yam, M. F. , Loh, Y. C. , Tan, C. S. , Adam, S. K. , Manan, N. A. , & Basir, R. (2018). General pathways of pain sensation and the major neurotransmitters involved in pain regulation. International Journal of Molecular Sciences, 19(8), 1–23. 10.3390/IJMS19082164 PMC6121522 30042373

[wsbm1570-bib-0289] Yang, Y. , Wang, Y. , Li, S. , Xu, Z. , Li, H. , Ma, L. , Fan, J. , Bu, D. , Liu, B. , Fan, Z. , Wu, G. , Jin, J. , Ding, B. , Zhu, X. , & Shen, Y. (2004). Mutations in SCN9A, encoding a sodium channel alpha subunit, in patients with primary erythermalgia. Journal of Medical Genetics, 41(3), 171–174. 10.1136/jmg.2003.012153 14985375 PMC1735695

[wsbm1570-bib-0290] Young, M. R. , Blackburn‐Munro, G. , Dickinson, T. , Johnson, M. J. , Anderson, H. , Nakalembe, I. , & Fleetwood‐Walker, S. M. (1998). Antisense ablation of type I metabotropic glutamate receptor mGluR1 inhibits spinal nociceptive transmission. The Journal of Neuroscience, 18(23), 10180. 10.1523/JNEUROSCI.18-23-10180.1998 9822771 PMC6793317

[wsbm1570-bib-0291] Yu, X. , Liu, H. , Hamel, K. A. , Morvan, M. G. , Yu, S. , Leff, J. , Guan, Z. , Braz, J. M. , & Basbaum, A. I. (2020). Dorsal root ganglion macrophages contribute to both the initiation and persistence of neuropathic pain. Nature Communications, 11(1), 1–12. 10.1038/s41467-019-13839-2 PMC6959328 31937758

[wsbm1570-bib-0292] Zhang, F. , Gigout, S. , Liu, Y. , Wang, Y. , Hao, H. , Buckley, N. J. , Zhang, H. , Wood, I. C. , & Gamper, N. (2019). Repressor element 1‐silencing transcription factor drives the development of chronic pain states. Pain, 160(10), 2398–2408. 10.1097/J.PAIN.0000000000001633 31206463 PMC6756259

[wsbm1570-bib-0293] Zhang, J.‐M. , & An, J. (2007). Cytokines, inflammation and pain. International Anesthesiology Clinics, 45(2), 27. 10.1097/AIA.0B013E318034194E 17426506 PMC2785020

[wsbm1570-bib-0294] Zhang, L. , Hoff, A. O. , Wimalawansa, S. J. , Cote, G. J. , Gagel, R. F. , & Westlund, K. N. (2001). Arthritic calcitonin/α calcitonin gene‐related peptide knockout mice have reduced nociceptive hypersensitivity. Pain, 89(2–3), 265–273. 10.1016/S0304-3959(00)00378-X 11166483

[wsbm1570-bib-0295] Zhao, J. , Lee, M.‐C. , Momin, A. , Cendan, C.‐M. , Shepherd, S. T. , Baker, M. D. , Asante, C. , Bee, L. , Bethry, A. , Perkins, J. R. , Nassar, M. A. , Abrahamsen, B. , Dickenson, A. , Cobb, B. S. , Merkenschlager, M. , & Wood, J. N. (2010). Small RNAs control Sodium Channel expression, nociceptor excitability, and pain thresholds. Journal of Neuroscience, 30(32), 10860–10871. 10.1523/JNEUROSCI.1980-10.2010 20702715 PMC6634685

[wsbm1570-bib-0296] Zhao, L. , Huang, J. , Fan, Y. , Li, J. , You, T. , He, S. , Xiao, G. , & Chen, D. (2019). Exploration of CRISPR/Cas9‐based gene editing as therapy for osteoarthritis. Annals of the Rheumatic Diseases, 78(5), 676–682. 10.1136/ANNRHEUMDIS-2018-214724 30842121 PMC6621547

[wsbm1570-bib-0297] Zhao, X. , Tang, Z. , Zhang, H. , Atianjoh, F. E. , Zhao, J.‐Y. , Liang, L. , Wang, W. , Guan, X. , Kao, S.‐C. , Tiwari, V. , Gao, Y.‐J. , Hoffman, P. N. , Cui, H. , Li, M. , Dong, X. , & Tao, Y.‐X. (2013). A long noncoding RNA contributes to neuropathic pain by silencing Kcna2 in primary afferent neurons. Nature Neuroscience, 16(8), 1024–1031. 10.1038/nn.3438 23792947 PMC3742386

[wsbm1570-bib-0298] Zhong, J. , Dietzel, I. D. , Wahle, P. , Kopf, M. , & Heumann, R. (1999). Sensory impairments and delayed regeneration of sensory axons in Interleukin‐6‐deficient mice. Journal of Neuroscience, 19(11), 4305–4313. 10.1523/JNEUROSCI.19-11-04305.1999 10341234 PMC6782624

[wsbm1570-bib-0299] Zhuang, Z.‐Y. , Wen, Y.‐R. , Zhang, D.‐R. , Borsello, T. , Bonny, C. , Strichartz, G. R. , Decosterd, I. , & Ji, R.‐R. (2006). A peptide c‐Jun N‐terminal kinase (JNK) inhibitor blocks mechanical allodynia after spinal nerve ligation: Respective roles of JNK activation in primary sensory neurons and spinal astrocytes for neuropathic pain development and maintenance. Journal of Neuroscience, 26(13), 3551–3560. 10.1523/JNEUROSCI.5290-05.2006 16571763 PMC6673862

[wsbm1570-bib-0300] Zimmer, A. , Valjent, E. , König, M. , Zimmer, A. M. , Robledo, P. , Hahn, H. , Valverde, O. , & Maldonado, R. (2001). Absence of Δ‐9‐tetrahydrocannabinol dysphoric effects in Dynorphin‐deficient mice. Journal of Neuroscience, 21(23), 9499–9505. 10.1523/JNEUROSCI.21-23-09499.2001 11717384 PMC6763924

[wsbm1570-bib-0301] Zimmer, A. , Zimmer, A. M. , Baffi, J. , Usdin, T. , Reynolds, K. , König, M. , Palkovits, M. , & Mezey, É. (1998). Hypoalgesia in mice with a targeted deletion of the tachykinin 1 gene. Proceedings of the National Academy of Sciences, 95(5), 2630–2635. 10.1073/PNAS.95.5.2630 PMC19441 9482938

[wsbm1570-bib-0302] Zimmermann, K. , Leffler, A. , Babes, A. , Cendan, C. M. , Carr, R. W. , Kobayashi, J. , Nau, C. , Wood, J. N. , & Reeh, P. W. (2007). Sensory neuron sodium channel Nav1.8 is essential for pain at low temperatures. Nature, 447(7146), 856–859. 10.1038/nature05880 17568746

